# Effect of Prehabilitation Before Total Knee Replacement for Knee Osteoarthritis on Functional Outcomes: A Randomized Clinical Trial

**DOI:** 10.1001/jamanetworkopen.2022.1462

**Published:** 2022-03-09

**Authors:** Christelle Nguyen, Isabelle Boutron, Alexandra Roren, Philippe Anract, Johann Beaudreuil, David Biau, Stéphane Boisgard, Camille Daste, Isabelle Durand-Zaleski, Bénédicte Eschalier, Charlotte Gil, Marie-Martine Lefèvre-Colau, Rémy Nizard, Élodie Perrodeau, Hasina Rabetrano, Pascal Richette, Katherine Sanchez, Jordan Zalc, Emmanuel Coudeyre, François Rannou

**Affiliations:** 1 Université de Paris, Faculté de Santé, Unités de Formation et de Recherche de Médecine, Paris, France; 2 Service de Rééducation et de Réadaptation de l’Appareil Locomoteur et des Pathologies du Rachis, Assistance Publique–Hôpitaux de Paris (AP-HP) Centre–Université de Paris, Hôpital Cochin, Paris, France; 3 INSERM UMRS-1124, Toxicité Environnementale, Cibles Thérapeutiques, Signalisation Cellulaire et Biomarqueurs (T3S), Paris, France; 4 AP-HP Centre-Université de Paris, Hôpital Hôtel-Dieu, Centre d’Épidémiologie Clinique, Paris, France; 5 METHODS Team, INSERM UMRS-1153, Centre de Recherche Épidémiologie et Statistique, Paris, France; 6 ECaMO Team, INSERM UMRS-1153, Centre de Recherche Épidémiologie et Statistique, Paris, France; 7 Institut Fédératif de Recherche sur le Handicap, Paris, France; 8 Service de Chirurgie Orthopédique, AP-HP Centre-Université de Paris, Hôpital Cochin, Paris, France; 9 Service de Médecine Physique et de Réadaptation, AP-HP Nord-Université de Paris, Hôpital Lariboisière, Paris, France; 10 Service de Chirurgie Orthopédique, Centre Hospitalo-Universitaire (CHU) de Clermont-Ferrand, Université Clermont Auvergne, Clermont-Ferrand, France; 11 Service de Médecine Physique et de Réadaptation, CHU de Clermont-Ferrand, Université Clermont Auvergne, Institut National de la Recherche Agronomique, Unité de Nutrition Humaine, Clermont-Ferrand, France; 12 Service de Chirurgie Orthopédique, AP-HP Nord-Université de Paris, Hôpital Lariboisière, Paris, France; 13 L’unité de Recherche Clinique en Économie de la Santé, Hôpital Hôtel-Dieu, Paris, France; 14 Service de Rhumatologie, AP-HP Nord-Université de Paris, Hôpital Lariboisière, Paris, France

## Abstract

**Importance:**

Multidisciplinary prehabilitation before total knee replacement (TKR) for osteoarthritis may improve outcomes in the postoperative period.

**Objective:**

To compare multidisciplinary prehabilitation with usual care before TKR for osteoarthritis in terms of functional independence and activity limitations after surgery.

**Design, Setting, and Participants:**

This prospective, open-label randomized clinical trial recruited participants 50 to 85 years of age with knee osteoarthritis according to the American College of Rheumatology criteria for whom a TKR was scheduled at 3 French tertiary care centers. Recruitment started on October 4, 2012, with follow-up completed on November 29, 2017. Statistical analyses were conducted from March 29, 2018, to March 6, 2019.

**Interventions:**

Four supervised sessions of multidisciplinary rehabilitation and education (2 sessions per week, at least 2 months before TKR, delivered to groups of 4-6 participants at each investigating center; session duration was 90 minutes and included 30 minutes of education followed by 60 minutes of exercise therapy) or usual care (information booklet and standard advice by the orthopedic surgeon) before TKR.

**Main Outcomes and Measures:**

The short-term primary end point was the proportion of participants achieving functional independence a mean (SD) of 4 (1) days after surgery defined as level 3 on the 4 functional tests. The midterm primary end point was activity limitations within 6 months after TKR assessed by the area under the receiver operating characteristic curve of the self-administered Western Ontario Questionnaire and McMaster Universities Osteoarthritis Index function subscale.

**Results:**

A total of 262 patients (mean [SD] age, 68.6 [8.0] years; 178 women [68%]) were randomized (131 to each group). A mean (SD) of 4 (1) days after surgery, 34 of 101 (34%) in the experimental group vs 26 of 95 (27%) in the control group achieved functional independence (risk ratio, 1.4; 97.5% CI, 0.9-2.1; *P* = .15). At 6 months, the mean (SD) area under the curve for the Western Ontario Questionnaire and McMaster Universities Osteoarthritis Index function subscale was 38.1 (16.5) mm^2^ in the experimental group vs 40.6 (17.8) mm^2^ in the control group (absolute difference, −2.8 mm^2^; 97.5% CI, −7.8 to 2.3; *P* = .31 after multiple imputation). No differences were found in secondary outcomes.

**Conclusions and Relevance:**

This randomized clinical trial found no evidence that multidisciplinary prehabilitation before TKR for osteoarthritis improves short-term functional independence or reduces midterm activity limitations after surgery.

**Trial registration:**

ClinicalTrials.gov Identifier: NCT01671917

## Key Points

**Question:**

What is the effectiveness of multidisciplinary prehabilitation compared with usual care before total knee replacement for knee osteoarthritis in terms of functional independence and activity limitations after surgery?

**Findings:**

In this randomized clinical trial of 262 participants, multidisciplinary prehabilitation before total knee replacement for knee osteoarthritis did not improve short-term functional independence or reduce midterm activity limitations after surgery.

**Meaning:**

Trial findings question the usefulness of prehabilitation before total knee replacement to improve functional outcomes.

## Teaser

This randomized clinical trial of older patients with knee osteoarthritis evaluates the effect of prehabilitation on functional independence and activity limitations after total knee replacement surgery.

## Introduction

Osteoarthritis is the most common osteoarticular disease and a leading cause of years lived with disability in the world.^1^ Osteoarthritis affects all anatomical structures of the joint and leads to progressive joint destruction. In the lower limbs, the knee joint is the most frequent osteoarthritis location. Conservative therapies that target symptoms (eg, pain, stiffness, and activity limitations) are recommended as first- and second-line treatments for knee osteoarthritis.^2,3,4,5^ When conservative therapies fail, total knee replacement (TKR) may be offered.

Physical and functional status before TKR is associated with recovery after surgery in people with knee osteoarthritis.^6^ Evidence suggests that physiotherapy, exercise therapy, occupational therapy, and education performed before TKR (ie, prehabilitation) as stand-alone interventions or in multidisciplinary rehabilitation education programs may slightly improve physical and functional outcomes in the perioperative period and reduce the length of hospital stay.^7,8,9,10,11,12,13,14,15,16,17^ However, large clinical trials assessing the short-term and midterm effects of this type of program are lacking. In a systematic review^12^ of 11 randomized clinical trials that compared physiotherapy-based programs to control groups on outcomes after TKR, 5 trials were rated as low to moderate quality (ie, Physiotherapy Evidence Database score <6 of 10), mostly because of small sample sizes, which ranged from 20^18^ to 131^19^ participants. In the current study, we aimed to compare multidisciplinary prehabilitation with usual care before TKR for osteoarthritis in terms of functional independence and activity limitations after surgery.

## Methods

### Design

This prospective, multicenter, 2-parallel-arm, open-label randomized clinical trial recruited participants starting on October 4, 2012, with follow-up completed on November 29, 2017. Statistical analyses were conducted from March 29, 2018, to March 6, 2019. We selected our primary and secondary effectiveness outcomes in accordance with the 2019 OMERACT-OARSI (Outcome Measures in Rheumatology–Osteoarthritis Research Society International) core domain set for measurement in clinical trials of hip and/or knee osteoarthritis^20^ and the 2015 OARSI clinical trials recommendations.^21^ Oral and written informed consent was obtained from all study participants. The protocol of the study was reviewed and approved by the CPP Île-de-France III on September 4, 2012. The trial protocol can be found in Supplement 1. All amendments to the protocol were approved by the CPP Île-de-France III institutional review board and are reported (eAppendix 1 in Supplement 2). Because of poor accrual and lack of further funding, enrollment was ended before the target sample size was reached. No changes in inclusion criteria or outcomes occurred after trial commencement. All primary and secondary prespecified effectiveness outcomes are reported (eAppendix 1 in Supplement 2). This study followed the Consolidated Standards of Reporting Trials (CONSORT) reporting guideline,^22,23^ and the interventions are described in accordance with the template for intervention description and replication checklist (eAppendix 1 in Supplement 2).^24^

### Randomization and Masking

An independent statistician from the Centre d’Épidémiologie Clinique provided a computer-generated randomization list with permuted, variable-size blocks (2, 4, and 6). The allocation ratio of assignments was 1:1. Randomization was stratified by center. Randomization and allocation concealment were performed by the investigator who identified the patient and involved use of a secured dedicated software (CleanWeb, Telemedicine Technologies). Statisticians and outcome assessors were blinded to the allocated group. Because of the nonpharmacological nature of the intervention, participants and health care practitioners could not be blinded.

### Participants

Participants were recruited from the departments of physical medicine and rehabilitation and orthopedics of 3 French tertiary care centers (Cochin, Lariboisière, and Clermont-Ferrand hospitals). Inclusion criteria were (1) age of 50 to 85 years; (2) knee osteoarthritis according to the American College of Rheumatology criteria, for which TKR was scheduled; (3) written informed consent to participate; and (4) affiliation with a health insurance program. Exclusion criteria were (1) institutionalization; (2) history of homolateral TKR; (3) history of chronic inflammatory joint disease; (4) cognitive or behavioral disorder; (5) insufficient proficiency in the French language; and (6) scheduled TKR for an indication other than knee osteoarthritis. We did not collect data on clinical physical examination (eg, range of motion and knee varus or valgus angulation), Kellgren and Lawrence scoring, proportion of cruciate-retaining prostheses, posterior-stabilized prostheses, or patellar replacement. Data on race and ethnicity were also not collected because they were not relevant to the research question.

### Interventions

#### Control Group

Participants allocated to the control group had usual care before TKR, including receipt of a previously validated information booklet^25,26^ (eAppendix 2 in Supplement 2) and standard advice given by their orthopedic surgeon. Standard advice was at the discretion of the orthopedic surgeon at each center and was not recorded. Pharmacological and nonpharmacological cointerventions were allowed in the control group and were recorded in the electronic case report form.

#### Experimental Group

Participants allocated to the experimental group received the information booklet^25,26^ (eAppendix 2 in Supplement 2), standard advice given by their orthopedic surgeon, and 4 supervised outpatient sessions of multidisciplinary rehabilitation and education (2 sessions per week) at least 2 months before TKR. We chose 2 months because previous studies^27,28^ suggested that the effects of education and rehabilitation on function are detectable from 6 weeks. The development of the experimental intervention followed the recommendations of the Medical Research Council for the development of complex nondrug interventions.^29^ Briefly, a group of experts with experience in the management of musculoskeletal disorders, including physiatrists, rheumatologists, orthopedic surgeons, physiotherapists, occupational therapists, a dietician, a psychologist, an instructor in physical activity, and a specialist in educational sciences, designed the intervention to be minimally burdensome and easily transposable to a community-based setting. To standardize the intervention, health care practitioners of each investigating center had a half-day face-to-face training with one of the investigators (K.S.) and were provided with standardized slideshows to use during supervised sessions (eAppendixes 3-8 in Supplement 2). For the first included participants, the investigators qualitatively verified the intervention fidelity across the 3 participating centers.

The experimental intervention was delivered to groups of 4 to 6 participants at each investigating center by trained health care practitioners. The session duration was 90 minutes and included 30 minutes of education (eAppendixes 3-7 in Supplement 2) followed by 60 minutes of exercise therapy (eAppendix 8 in Supplement 2). Educational material included standardized slideshows, a DVD of exercises, and group discussions. The educational program included a first session about the positive effects of exercise therapy before TKR (eAppendix 3 in Supplement 2) conducted by a physiotherapist or an instructor in physical activity, a second session about work rehabilitation and social support (eAppendix 4 in Supplement 2) and diet and weight management (eAppendix 5 in Supplement 2) conducted by a social worker and a dietician, a third session about the management of stress and anxiety in the perioperative period (eAppendix 6 in Supplement 2) conducted by a psychologist, and a fourth session about the return home (eAppendix 7 in Supplement 2) conducted by an occupational therapist. The exercise therapy program consisted of 4 sessions supervised by a physiotherapist that included muscle strengthening, lower-limb stretching, endurance training, proprioception exercises, and walking and balance exercises; training to perform the 4 functional tests described by Zavadak et al^30^; and a home-based program (eAppendix 8 in Supplement 2). We did not implement specific measures to enhance participants’ adherence to the supervised sessions or home-based program and did not perform specific monitoring of the home-based program. Pharmacological and nonpharmacological cointerventions were allowed in the experimental group and were recorded in the electronic case report form.

### Outcomes

#### Primary Outcomes

The Medical Research Council recommends considering multiple assessment criteria for evaluating complex interventions.^29^ Therefore, we prespecified 2 primary end points. The short-term primary end point was the proportion of participants achieving functional independence a mean (SD) of 4 (1) days after surgery, assessed by a blinded physiotherapist as the ability to perform the 4 functional tests described by Zavadak et al^30^ at level 3: transfer from lying to sitting, transfer from sitting to standing, walking 30 m, and going up and down a flight of stairs. These tests can be performed with 4 levels of independence: level 0, not possible; level 1, possible with someone’s physical help; level 2, possible with someone’s verbal help; and level 3, possible without help.^30,31,32,33,34,35,36^ At all levels, the use of walking aids was allowed. The physiotherapist who assessed outcomes did not participate in the experimental intervention and was instructed to refrain from asking questions about the preoperative period. The midterm primary end point was activity limitations within 6 months after TKR, assessed by the area under the receiver operating characteristic curve (AUC) of the self-administered Western Ontario Questionnaire and McMaster Universities Osteoarthritis Index (WOMAC) function subscale (with 0 indicating no limitations and 100 indicating maximal limitations).^37^

#### Secondary Outcomes

Secondary end points were collected at 6 and 12 months after TKR and included (1) mean knee pain during the last 48 hours, assessed by a self-administered numeric rating scale (with 0 indicating no pain and 100 indicating maximal pain); (2) mean activity limitations assessed by the self-administered WOMAC function subscale; (3) mean health-related quality of life (HRQoL) assessed by the physical component score (with 9.95 indicating worst HRQoL and 70.02 indicating best HRQoL) and mental component score (with 5.89 indicating worst HRQoL and 71.97 indicating best HRQoL)^38^ and by the EuroQol 5-Dimension scale^39,40^; (4) mean number of steps in the previous week measured by using a pedometer; and (5) the cost-utility ratio assessed by comparing total costs between the experimental and control groups.

#### Safety Outcomes

Safety outcomes were recorded at each follow-up visit by using the open-ended question, “Did you have any adverse events?” Serious adverse events were reviewed by ^2^ blinded investigators (C.D. and C.G.). Events were classified into 6 categories: (1) hospitalization for usual care after TKR, (2) hospitalization for another reason, (3) hospitalization related to the intervention received, (4) usual care after TKR without hospitalization, (5) usual care for another reason without hospitalization, and (6) usual care related to the intervention received without hospitalization.

### Cost-effectiveness Analysis

Information on the consumption of resources by patients in each group was collected in the case report form and valued from the point of view of the health care system with available hospital accounting data. The resources collected included the time spent before TKR by the surgeons in the control group and by the teams in charge of education and rehabilitation in the experimental group, the duration of hospitalization, and the length of stay in the rehabilitation department. In principle, no structural costs were associated with the intervention because it used existing capacities. Professionals’ time was valued by gross salary and hospital admissions by the diagnosis-related group cost adjusted for actual length of stay in the acute care and rehabilitation wards. The same method was used for adverse events that led to an admission. Costs were calculated per patient and compared by randomization group based on intention to treat, using bootstrap hypothesis testing to avoid relying on normality assumptions.

### Statistical Analysis

We hypothesized that 20% of participants would achieve functional independence a mean (SD) of 4 (1) days after surgery in the control group. A sample of 130 participants per group was required to demonstrate a risk ratio of 2.0 with a power of 90% and an α risk set at 2.5% in bilateral formulation to account for the 2 primary end points. A sample of 130 participants per arm was needed to demonstrate an effect size of 0.45 in comparing the mean AUC for the WOMAC function subscale with a power of 90% and an α risk set at 2.5%. With an estimated 10% of participants unavailable for follow-up, 144 patients per group were needed. Rounding this number to the upper 10 units, we sought to include 300 participants (150 participants per group).

Primary effectiveness analysis was conducted as intention to treat: all randomized patients were analyzed for the primary outcome in their group of randomization. The proportion of patients who achieved functional independence by a mean (SD) of 4 (1) days after surgery in each group and their ratio were estimated with an adjusted robust Poisson model. The adjustment variables were the level of expertise of the surgeon (junior or senior) and the center. Adjustment variables were chosen a priori; randomization was stratified on center and expertise of surgeon because it was expected that it would have an effect on the global success of the intervention. The adjusted difference in proportions was estimated with a linear model.

The AUC of the WOMAC function subscale between inclusion and 6 months was calculated by the trapezium method. For each patient, the total AUC is divided by the actual follow-up time to consider the differences in time between visits. We therefore obtain an estimate of the mean WOMAC function subscale score between inclusion and the 6-month visit. The AUC means were compared with an adjusted linear model, with the randomization arm, center, and level of expertise of the surgeon as explanatory variables and the AUC of the WOMAC function subscale as the response variable. The mean difference is derived from this model.

To handle missing data for the 2 coprimary end points, multiple imputations by chained equations was used, with separate imputation in each arm. Baseline characteristics (sex, age, level of education, professional status, weight, height, arterial hypertension, diabetes, arthritis, traumatic history, surgical history, gonarthrosis, destination at discharge from acute care, knee pain, mean number of steps, WOMAC score, Short Form Medical Outcomes (SF-12) mental score, and SF-12 physical score), center, and coprimary outcomes were used in the prediction model. Imputation was performed by predictive mean matching for continuous data, logistic regression for binary variables, and polytomous regression for other categorical variables. The last type of variables is not part of the primary outcome but is necessary for the imputation algorithm.

We used 10 iterations of the multiple imputation by chained equations (MICE) procedure,^41^ and 48 imputations were used following the recommendation to use as many imputations as the percentage of observations with missing data.^42^ Convergence of the multiple imputation algorithm and the results of the imputation were checked. Imputations were performed with the R MICE package.^43^ Analyses were then performed on each of the imputed data sets and pooled according to Rubin’s rules. The tests on the judgment criteria were declared significant at the 2.5% threshold to account for the dual primary end points. The results of the adjusted models were compared with the unadjusted model.

All secondary outcomes (WOMAC, pain, SF-12 mental and physical scores, number of steps, and satisfaction) were analyzed with constrained longitudinal data analysis.^44^ Linear mixed models were used to estimate the adjusted mean differences at 6 and 12 months with the quantitative criteria at each time as a response variable with a constraint of a null difference for the baseline values; randomization group, visit, interaction group by visit, center, and level of expertise as explanatory variables (fixed effects); and patient (intercept + slope [time from inclusion]) as a random effect. This technique of analysis is compatible with the principle of intention-to-treat analysis provided that all patients have at least 1 value supplied by the judgment criterion. The use of multiple imputation does not improve the results for mixed models for longitudinal data, and the use of mixed models for imputed data can sometimes lead to unstable results^45^; therefore, no imputation was performed for those outcomes. Descriptive analysis was conducted for safety end points.

For all analyses, a 2-sided *P* < .05 was considered statistically significant. All analyses were performed by an independent and blinded statistician (E.P.) with R software, version 3.4.4 (R Foundation for Statistical Computing).

## Results

### Participants

A total of 262 patients were randomized (mean [SD] age, 68.6 [8.0] years; 178 women [68%] and 84 men [32%]): 131 in the experimental group (multidisciplinary rehabilitation education program) and 131 in the control group (usual care). The mean (SD) pain duration was 9.9 (8.7) years. The mean (SD) time between randomization and surgery was 2.3 (1.7) months in the experimental group and 2.2 (1.1) months in the control group (Table 1). In the experimental group, 43 of 131 patients (33%) did not attend any supervised sessions (Figure).

**Table 1.** Demographic and Clinical Characteristics of Participants^a^

Characteristic	Rehabilitation education (n = 131)	Usual care (n = 131)	Total (N = 262)
Age, mean (SD), y	68.2 (7.3)	68.9 (8.7)	68.6 (8.0)
Sex			
Female	91 (69)	87 (66)	178 (68)
Male	40 (31)	44 (34)	84 (32)
BMI, mean (SD)^b^	29.5 (4.9)	29.4 (5.5)	29.4 (5.2)
Higher educational level^c^	51 (39)	46 (35)	97 (37)
Traumatic history^b^			
Meniscal lesion	11 (8)	20 (15)	31 (12)
Cruciate ligament injury			
Anterior	13 (10)	16 (12)	29 (11)
Posterior	2 (2)	2 (2)	4 (2)
Proximal tibial fracture	1 (1)	2 (2)	3 (1)
Surgical history^b^			
Meniscal surgery	17 (13)	29 (22)	46 (18)
Tibial osteotomy	5 (4)	12 (9)	17 (7)
Ligamentoplasty	7 (5)	8 (6)	15 (6)
Previous treatments^c^			
Intra-articular injection of hyaluronic acid	91 (70)	91 (70)	182 (70)
Intra-articular injection of corticosteroids	83 (64)	71 (56)	154 (60)
Knee brace	70 (54)	60 (46)	130 (50)
Walking aids	70 (54)	60 (46)	130 (50)
Ongoing treatments^c^			
Analgesics			
Nonopioid	54 (42)	55 (42)	109 (42)
Weak opioid^d^	42 (32)	49 (38)	91 (35)
Strong opioid^d^	0	2 (2)	2 (1)
Nonsteroidal anti-inflammatory drugs	48 (37)	46 (35)	94 (36)
Physical therapy	24 (19)	20 (15)	44 (17)
Foot orthoses	24 (19)	17 (13)	41 (16)
Weight management	24 (19)	17 (13)	41 (16)
Clinical characteristics			
Right knee operated^b^	68 (52)	72 (55)	140 (54)
Pain duration, mean (SD), y^c^	8.6 (7)	10.5 (8)	9.5 (8)
Pain intensity (NRS scores, 0-100), mean (SD)^c^^,^^e^	53.0 (23.7)	55.7 (21)	54.3 (22)
No. of steps a day during the week after inclusion, mean (SD)^f^	3797 (2097)	3949 (3077)	3876 (2649)
WOMAC function subscore (range, 0-100), mean (SD)^g^^,^^h^	47.9 (18.2)	46.8 (18.0)	47.3 (18.1)
SF-12, mean (SD)^i^^,^^j^			
Physical component score (range, 9.95-70.02)	32.7 (6.9)	32.9 (6.7)	32.8 (6.8)
Mental component score (range, 5.89-7.97)	43.1 (10.9)	42.6 (10.3)	42.8 (10.6)
RAPT total score (range, 0-12), mean (SD)^k^	8.6 (2.1)	8.6 (2.2)	8.6 (2.1)
Time between randomization and surgery, mean (SD), mo	2.3 (1.7)	2.2 (1.1)	2.3 (1.4)
Time between the last session of prehabilitation and surgery, mean (SD), mo	1.7 (1.4)	NA	NA

Abbreviations: BMI, body mass index (calculated as weight in kilograms divided by height in meters squared); NA, not applicable; NRS, numeric rating scale (with 0 indicating no limitations and 100 indicating maximal limitations); RAPT, Risk Assessment and Predictor Tool (with 0 indicating highest risk and 12 indicating lowest risk); SF-12, 12-item Short-Form Medical Outcomes Study (with 9.95 indicating worst health-related quality of life and 70.02 indicating best health-related quality of life for the physical component score and 5.89 indicating worst health-related quality of life and 71.97 indicating best health-related quality of life for the mental component score); WOMAC, Western Ontario and McMaster Universities Osteoarthritis Index.

^a^ Data are presented as number (percentage) of patients unless otherwise indicated.

^b^ Data missing for 1 patient in the usual care group.

^c^ Data missing for 2 patients (1 in the rehabilitation education group and 1 in the usual care group).

^d^ Weak opioids include codeine, dihydrocodeine, and tramadol. Strong opioids include morphine, diamorphine, fentanyl, buprenorphine, oxymorphone, oxycodone, and hydromorphone.

^e^ Higher scores indicate greater pain.

^f^ Data missing for 90 patients (49 in the rehabilitation education group and 41 in the usual care group).

^g^ Higher scores indicate more limitations.

^h^ Data missing for 11 patients (7 in the rehabilitation education group and 4 in the usual care group).

^i^ Higher scores indicate better health.

^j^ Data missing for 3 patients (2 in the rehabilitation education group and 1 in the usual care group).

^k^ Destination at discharge from acute care predicted by score (<6 indicating extended inpatient rehabilitation; 6-9, additional intervention to discharge directly home [eg, rehabilitation in the home]; and 9, directly home).

**Figure.**
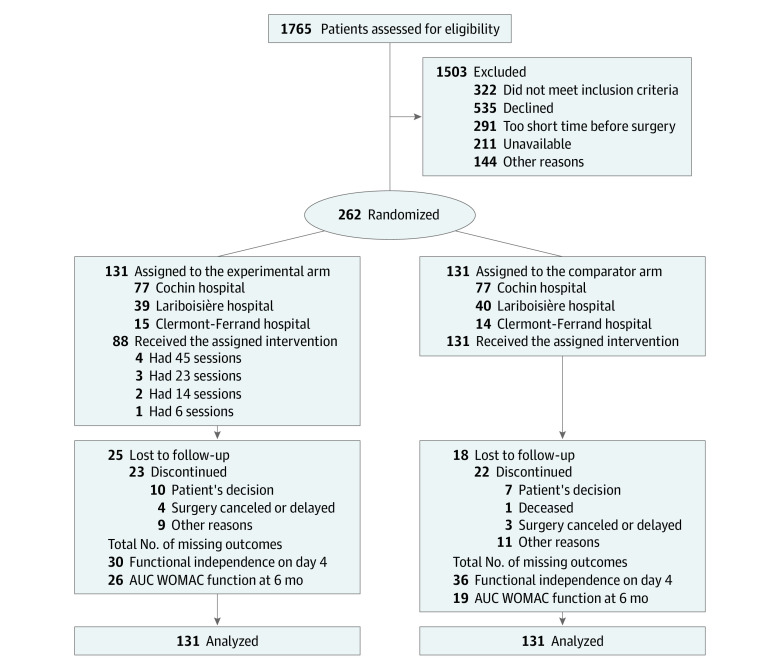
Study Flow Diagram AUC indicates area under the curve; WOMAC, Western Ontario Questionnaire and McMaster Universities Osteoarthritis Index.

### Primary Outcomes

A mean (SD) of 4 (1) days after surgery, 34 of 101 patients (34%) in the experimental group vs 26 of 95 patients (27%) in the control group achieved functional independence (relative risk, 1.4; 97.5% CI, 0.9-2.1; *P* = .15; absolute difference in risk, 8.9; 97.5% CI, −5.0 to 22.7 after multiple imputation). At 6 months, the mean (SD) AUC of the WOMAC function subscale was 38.1 (16.5) mm^2^ in the experimental group vs 40.6 (17.8) mm^2^ in the control group (absolute difference, −2.8 mm^2^; 97.5% CI, −7.8 to 2.3; *P* = .31 after multiple imputation) (Table 2).

**Table 2.** Functional Independence on a Mean (SD) of 4 (1) Days After Surgery and Activity Limitations Within 6 Months After Total Knee Arthroplasty

Outcome	Rehabilitation education (n = 131)	Usual care (n = 131)	Absolute difference (97.5% CI)	Risk ratio (97.5% CI)	*P* value
**Functional independence, No./total No. (%)^a^**					
Imputed data^b^	46/131 (35)	34/131 (26)	8.9 (−5.0 to 22.7)	1.3 (0.9 to 2.1)	.15
Complete cases^c^	34/101 (34)	26/95 (27)	7.9 (−6.6 to 22.3)	1.3 (0.8 to 2.1)	
Without adjustment	46/131 (35)	34/131 (26)	8.8 (−5.2 to 22.7)	1.3 (0.8 to 2.1)	
**Activity limitations (AUC of the WOMAC function subscale), mean (SD) mm^2^**					
Imputed data^b^	38.2 (16.3)	41.3 (18.3)	−2.8 (−7.8 to 2.3)	NA	.31
Complete cases^c^	38.1 (16.5)	40.6 (17.8)	−2.2 (−7.3 to 3.0)	NA	
Without adjustment	38.2 (16.3)	41.3 (18.3)	−3.1 (−8.2 to 2.0)	NA	

Abbreviations: AUC, area under the receiver operating characteristic curve; NA, not applicable; WOMAC, Western Ontario and McMaster Universities Osteoarthritis Index.

^a^ Participants were considered independent if they were able to perform the 4 functional tests described by Zavadak et al^30^ at level 3: transfer from lying to sitting, transfer from sitting to standing, walking 30 m, and going up and down a flight of stairs. These tests can be performed with 4 levels of independence (level 0, not possible; level 1, possible with someone’s physical help; level 2, possible with someone’s verbal help; and level 3, possible without help). At all levels, the use of walking aids was allowed.

^b^ Adjusted for the center and the level of expertise of the surgeon.

^c^ The complete cases correspond to an analysis removing the patients with missing data (105 in the rehabilitation education group and 112 in the usual care group).

### Secondary Outcomes

The 2 groups did not differ in any of the secondary end points. We found no evidence of reduced pain or activity limitations, improved health-related quality of life, or increased number of steps in the previous week in the experimental group compared with the control group at 6 and 12 months after surgery (Table 3).

**Table 3.** Outcomes at 6 and 12 Months After Surgery

Outcome	Mean (SD)		Adjusted mean differences (95% CI)^a^	*P* value
	Rehabilitation education (n = 131)	Usual care (n = 131)		
**6 mo after surgery**				
Pain intensity (NRS scores, 0-100)	24.5 (21.4)	25.7 (23.2)	0.5 (−5.5 to 6.5)	.86
WOMAC function score (range, 0-100)	47.1 (19.7)	49.7 (18.1)	−3.2 (−8.8 to 2.3)	.26
SF-12				
Physical component score (range, 9.95-70.02)	41.1 (7.8)	38.7 (9.1)	2.0 (−0.2 to 4.1)	.08
Mental component score (range, 5.89-71.97)	45.1 (11.2)	45.8 (11.3)	−1.1 (−3.8 to 1.7)	.44
No. of steps in the previous week	3981 (2061)	3712 (2161)	−94 (−1030 to 841)	.84
Satisfaction with care (NRS scores, 0-100)	68.8 (25.9)	73.0 (26.1)	−4.3 (−12.8 to 4.1)	.31
EQ-5D score (range, 0-3)	0.6 (0.3)	0.6 (0.3)	0.0 (−0.1 to 0.1)	.87
**12 mo after surgery**				
Pain intensity (NRS scores, 0-100)	25.9 (26.5)	23.1 (21.8)	3.9 (−3.5 to 11.2)	.30
WOMAC function score (range, 0-100)	44.9 (20.2)	48.3 (19.4)	−3.2 (−9.6 to 3.2)	.33
SF-12				
Physical component score (range, 9.95-70.02)	41.7 (8.7)	40.7 (9.2)	0.8 (−1.8 to 3.4)	.54
Mental component score (range, 5.89-71.97)	46.8 (11.3)	47.1 (11.2)	−0.2 (−3.2 to 2.8)	.90
No. of steps in the previous week	4969 (3481)	4600 (3757)	328 (−771 to 1427)	.56
Satisfaction with care (NRS score, 0-100)	72.1 (29.9)	73.7 (28.8)	−2.2 (−10.9 to 6.4)	.62
EQ-5D score (range, 0-3)	0.7 (0.3)	0.6 (0.3)	0.1 (−0.1 to 0.0)	.70
Total costs, €	15 573 (7247)	15 987 (6519)	−414 (−1739 to 2158)	.64

Abbreviations: EQ-5D, European Quality of Life–5 Dimensions; NRS, numeric rating scale (with 0 indicating no limitations and 100 indicating maximal limitations); SF-12, 12-item Short-Form Medical Outcomes Study (with 9.95 indicating worst health-related quality of life and 70.02 indicating best health-related quality of life for the physical component score and 5.89 indicating worst health-related quality of life and 71.97 indicating best health-related quality of life for the mental component score); WOMAC, Western Ontario and McMaster Universities Osteoarthritis Index.

^a^ Difference between rehabilitation education and usual care.

### Cost-effectiveness Outcome

Total mean (SD) costs were €15 573 (€7247) in the experimental group vs €15 987 (€6519) in the control group. The mean (SD) European Quality of Life–5 Dimensions (EQ-5D) score was 0.6 (0.3) in the experimental group and 0.6 (0.3) in the control group at 6 months after surgery and 0.7 (0.3) vs 0.6 (0.3) at 12 months after surgery (Table 3). Cointerventions received at 3, 6, and 12 months are reported in eAppendix 9 in Supplement 2, and detailed resource use and costs are reported in eAppendix 10 in Supplement 2.

### Adverse Events

A total of 48 of 131 patients (37%) in the experimental group vs 47 of 131 patients (36%) in the control group had at least 1 minor adverse event. A total of 18 of 131 patients (14%) in the experimental group vs 12 of 131 patients (9%) in the control group had at least 1 serious adverse event (Table 4).

**Table 4.** Serious and Minor Adverse Events

Adverse event	Rehabilitation exercise (n = 131)	Usual care (n = 131)	Total (N = 262)
Serious adverse events			
Total No. of serious adverse events	24	17	41
Hospitalization for usual care after total knee replacement	7	6	13
Hospitalization for another reason	14	10	24
Hospitalization related to the intervention received	3	1	4
Minor adverse events			
Total No. of minor adverse events	78	72	150
Cancer	1	1	2
Cardiovascular symptoms	2	1	3
Complex regional pain syndrome	1	0	1
Depression	4	3	7
Dizziness	1	1	2
Fall	3	5	8
Fatigue	2	0	2
Fracture	0	1	1
Hematologic symptoms	2	3	5
Hematoma	1	1	2
Knee pain and/or stiffness	17	19	36
Other musculoskeletal pain	18	10	28
Infection	1	1	2
Systemic inflammatory symptoms	1	0	1
Metabolic symptoms	0	2	2
Neurologic symptoms	3	9	12
Respiratory symptoms	8	1	9
Skin symptoms	3	1	4
Thrombophlebitis	5	4	9
Unspecified	1	6	7
Urodigestive symptoms	2	3	5
Venous insufficiency	2	0	2

## Discussion

This randomized clinical trial found no evidence that multidisciplinary prehabilitation before TKR for osteoarthritis improved short-term functional independence or reduced midterm activity limitations after surgery. The uptake of the experimental intervention was low.

Regarding functional independence, we found no evidence of the benefit of our program, contrary to 2 previously published randomized clinical trials^46,47^ of 122 and 133 participants, respectively, that showed positive effects of multidisciplinary approaches before TKR to reduce the mean length of hospital stay in populations similar to ours. Several reasons could explain our results. In our experimental group, only one-third of the patients attended all supervised sessions. The low adherence to supervised sessions may have led to a dilution of the specific effects of our treatment. Our population had severe osteoarthritis and high activity limitation levels, which could have resulted in a high perceived burden of treatment.^48^ Consistently, in a population of 133 patients with knee osteoarthritis and complex needs, Crowe et al^46^ reported low adherence (48%) to a short multidisciplinary rehabilitation education program. Our findings, consistent with those of Crowe et al,^46^ suggest that in a severely disabled population even short outpatient supervised rehabilitation programs may be burdensome. Prehabilitation should probably include more targeted modalities than the ones delivered in the current study. For example, a preoperative, simplified, home-based rehabilitation education program could represent a more acceptable solution in this population.^11^ Finally, our intervention was delivered 2 months before TKR, so the specific effects of the supervised sessions may not have lasted until postoperative assessments.

Regarding activity limitations after TKR, a systematic review^12^ of 5 randomized clinical trials that compared the efficacy of heterogeneous physiotherapy-based programs before TKR, varying from 3 to 8 weeks and including 6 to 30 sessions, did not show evidence of reduced activity limitations from 6 weeks to 12 months after TKR by using the WOMAC score. We found no evidence that our intervention improved any of the secondary end points (ie, pain, quality of life, level of physical activity, and costs). Even though these outcomes belong to the core outcome set for knee osteoarthritis, our intervention may not have been specifically designed to target these dimensions. The large treatment effects of TKR could also have masked the effects of our program. Furthermore, one cannot exclude a positive effect of the information booklet and standard advice given by the orthopedic surgeon in the control group.

### Limitations

This study has some limitations. The lack of blinding of participants could have led to detection biases. Our population was recruited from tertiary care centers, which limits the external validity of our study. Adherence to the home-based program may have influenced outcomes but was not monitored. Our study may be underpowered because of missing outcomes and low adherence. A qualitative study could help to understand the reasons for the low adherence and to tailor strategies to enhance adherence for further implementation of the intervention in clinical practice (eg, home-based program, facilitated transportation to the rehabilitation center, and coaching measures adapted to our population). Finally, data that could have been useful to interpret our results, including results of clinical physical examination, Kellgren and Lawrence scoring, proportion of cruciate-retaining prostheses, posterior-stabilized prostheses, or patellar replacement, are lacking.

## Conclusions

This randomized clinical trial found no evidence that a multidisciplinary rehabilitation education program before TKR improves functional independence or reduces activity limitations in people with knee osteoarthritis after surgery. However, the interpretation of the results of this trial is limited by the low uptake of the experimental intervention.

## Notes

Supplement 1.
Trial Protocol and Statistical Analysis Plan

Supplement 2. **eAppendix 1.** Substantial Changes to Methods After Trial Commencement and the Reasons for the Changes **eAppendix 2.** Original Version (French) of the Booklet “*Vous allez bénéficier d’une prothèse de genou*” **eAppendix 3.** English Translation of the Slideshow Used for the Educational Program on the Positive Effects of Exercise Therapy Before Total Knee Replacement Conducted by a Physiotherapist or an Instructor in Physical Activity **eAppendix 4.** English Translation of the Slideshow Used for the Educational Program on Work Rehabilitation and Social Support Conducted by a Social Worker **eAppendix 5.** English Translation of the Slideshow Used for the Educational Program on Diet and Weight Management Conducted by a Dietician **eAppendix 6.** English Translation of the Slideshow Used for the Educational Program on the Management of Stress and Anxiety in the Perioperative Period Conducted by a Psychologist **eAppendix 7.** English Translation of the Slideshow Used for the Educational Program on the Return Home Conducted by an Occupational Therapist **eAppendix 8.** Supervised Exercise Therapy Sessions **eAppendix 9.** Non-pharmacological and Pharmacological Co-interventions **eAppendix 10.** Twelve-Month Costs in the Intervention and Control Groups; Costs Are in Euros

Supplement 3.
Data Sharing Statement
