# Supplementary material for: Effect of Prehabilitation Before Total Knee Replacement for Knee Osteoarthritis on Functional Outcomes: A Randomized Clinical Trial
Source: JAMA Netw Open. 2022 Mar 9;5(3):e221462. doi: 10.1001/jamanetworkopen.2022.1462 (PMC8908069; doi:10.1001/jamanetworkopen.2022.1462)
Supplement: Supplement 1. — Trial Protocol and Statistical Analysis Plan [file jamanetwopen-e221462-s001.pdf]

ASSISTANCE PUBLIQUE HÔPITAUX DE PARIS

EVALUATION OF AN EDUCATIONAL PROGRAM ASSOCIATED WITH EXERCISES (EDEX) BEFORE TOTAL KNEE ARTHROPLASTY

# - EDEX -

VERSION no. 1.0 dated 23/04/2012

**Sponsor:**	Assistance Publique des Hôpitaux de Paris<br>Regional Delegation of Clinical Research (DRCD)<br>Carré Historique – Hôpital Saint louis<br>1, avenue Claude Vellefaux<br>75010 Paris<br>Project Leader: Karine Goude-Ory<br>Tel.: (+33) 01 44 84 17 22 / Fax: (+33) 01 44 84 17 01
**Principal Investigator:**	Prof. Serge Poiraudeau<br>Department of Physical Medicine and Rehabilitation of the Musculoskeletal System and Spinal Disorders<br>Hôpital Cochin,<br>27 rue du Faubourg St Jacques<br>75679 PARIS Cedex 14<br>Tel.: (+33) 01 58 41 25 49 / Fax: (+33) 01 58 41 25 45<br>Email: serge.poiraudeau@aphp.fr
**Methodology and statistical analysis:**	Dr. Isabelle Boutron, Prof. Philippe Ravaud,<br>Clinical Epidemiology Centre<br>Hôpital Hôtel Dieu<br>1, place du Parvis Notre Dame 75004 Paris<br>Tel.: (+33) 01 40 25 79 43
**Health economics evaluation:**	Prof. Isabelle Durand-Zaleski<br>URCEco Ile de France<br>GH Albert Chenevier-Henri Mondor<br>51 avenue du Maréchal de Lattre de Tassigny<br>94010 Créteil
**Clinical Research Unit:**	CRU Cochin - Necker<br>Site Tarnier<br>89 rue d'Assas<br>75006 PARIS<br>Tel.: (+33) 01 58 41 28 84 / Fax: (+33) 01 58 41 11 83

Biomedical research PROTOCOL SIGNATURE page
for the PRINCIPAL investigator and the SPONSOR's representative

Biomedical research study no. 100122 *code:* ***EDEX AOM 10042***

ANSM registration number: **2012-A00208-35**

Title: *"Evaluation of an Educational Program Associated With Exercises (EDEX) Before Total Knee Arthroplasty"*

Version no. 1.1 dated 23/04/2012

Principal Investigator:

Prof. Serge Poiraudeau

Department of Physical Medicine and Rehabilitation of the Musculoskeletal System and Spinal Disorders
Hôpital Cochin,
27 rue du Faubourg St Jacques
75679 PARIS Cedex 14

Date:

Signature:

Sponsor:

Assistance Publique – Hôpitaux de Paris
Regional Delegation of Clinical Research
Hôpital Saint Louis
75010 PARIS

Date: ……………/………/………..

Signature:

NB: This version corresponds to the text from the protocol and annexes sent to the Ethics Committee (CPP) for its opinion and to the competent authority for authorisation.

TABLE OF CONTENTS

Summary 5

I - INTRODUCTION 7

I.1 - INFORMATION IN THE LITERATURE AND JUSTIFICATION OF THE RESEARCH 7
I.2 - EXPECTED RESULTS AND PROSPECTS 9
I.3 - STUDY FEASIBILITY 9
I.4 - EXPECTED BENEFITS AND RISKS 9

II - STUDY OBJECTIVE 10

II.1 - PRIMARY OBJECTIVE 10
II.2 - SECONDARY OBJECTIVES 10

III - METHODOLOGY 10

III.1 - STUDY TYPE 10
III.2 - STUDY DURATION 11
III.3 - EXPERIMENTAL DESIGN 11
III.4 - PATIENT ENROLMENT AND RANDOMISATION 12

IV - STUDY POPULATION 12

IV.1 - INCLUSION CRITERIA 12
IV.2 - EXCLUSION CRITERIA 13
IV.3 - REQUIRED NUMBER OF SUBJECTS 13
IV.4 - DIAGNOSIS STANDARDISATION 13
IV.5 - RECRUITMENT METHOD 13

V - STUDY TREATMENT 13

V.1 - TREATMENT OUTLINE 13
V.2 - ASSOCIATED TREATMENTS 16
V.2.1 - Authorised treatments 16
V.2.2 - Prohibited treatments 16
V.3 - PROVISIONS PUT IN PLACE TO COMPENSATE FOR THE LACK OF BLINDING 16
V.4 - PATIENT CARD 16

VI - ENDPOINTS 16

VI.1 - PRIMARY ENDPOINTS 16
VI.2 - SECONDARY ENDPOINTS 17

VII - PATIENT STUDY PLAN 18

General outline of the study 18
VII.1 - VISIT V0 - ENROLMENT VISIT 19
VII.2 - INTERVENTION PROTOCOL 20
VII.3 - FOLLOW-UP VISITS AFTER THE INTERVENTION 21
VII.3.1 - Preoperative visit (V1) 21
VII.3.2 - Visits during the stay at the surgical department (V2) 22
VII.3.3 - Contact by post or email 6 weeks after surgery 22
VII.3.4 - Contact 3 months after surgery (V2) 22
VII.3.5 - Contact by post or email 4 and 5 months after surgery 22
VII.3.6 - Contact by post or email 6 months after surgery .... ***Erreur ! Signet non défini.***
VII.3.7 - Contact 12 months after surgery (V4) .... ***Erreur ! Signet non défini.***
VII.4 - EXITING THE TRIAL AND EARLY WITHDRAWAL 23
VII.5 - PROCEDURES FOR LIMITING MISSING DATA 23
VII.6 - SPECIFIC CHARACTERISTICS OF THE STUDY IN RELATION TO THE PATIENT'S USUAL CARE 23
VII.7 - DESCRIPTION OF THE RULES FOR PARTIAL OR FULL TERMINATION OF THE STUDY 24

VIII - QUALITY CONTROL AND ASSURANCE 24

VIII.1 - MONITORING PROCEDURES 24
VIII.2 - TRANSCRIPTION OF INFORMATION INTO THE CASE REPORT FORM 25

IX - STATISTICAL DATA MANAGEMENT 26

IX.1 - RESPONSIBILITY FOR THE DATA ANALYSIS AND SOFTWARE USED 27
IX.2 - JUSTIFICATION OF THE REQUIRED NUMBER OF SUBJECTS 27

IX.3 - ANALYSIS STRATEGY FOR COLLECTED DATA 27
IX.4 - JUSTIFICATION OF STATISTICAL TESTS 27

X - SAFETY ASSESSMENT 30

X.1 - DEFINITIONS 30
X.2 - DESCRIPTION OF THE SAFETY ASSESSMENT PARAMETERS .... **ERREUR ! SIGNET NON DEFINI.**
X.3 - SERIOUS ADVERSE EVENT MANAGEMENT PROCEDURE .... **ERREUR ! SIGNET NON DEFINI.**
X.4 - STUDY-SPECIFIC COMMITTEES 31
X.4.1 - Steering committee 31
X.4.2 - Independent monitoring committee 32

XI - RIGHT TO ACCESS THE INFORMATION AND SOURCE DOCUMENTS 32

XII - LEGAL AND ETHICS ASPECTS 32

XII.1 - REQUEST FOR AUTHORISATION FROM THE ANSM 33
XII.2 - REQUEST FOR OPINION FROM THE ETHICS COMMITTEE 33
XII.3 - AMENDMENTS 33
XII.4 - CNIL DECLARATION 33
XII.5 - INFORMATION SHEET AND INFORMED CONSENT FORM 34
XII.6 - FINAL STUDY REPORT 34

XIII - DATA PROCESSING AND STORAGE OF STUDY-RELATED DOCUMENTS AND DATA 34

XIV - INSURANCE AND SCIENTIFIC COMMITMENT 35

XIV.1 - INSURANCE 35
XIV.2 - PRINCIPAL INVESTIGATOR'S COMMITMENT 35

XV - RULES REGARDING PUBLICATION 35

XVI - LIST OF ANNEXES 37

Annex 1 – Literature references 38
Annex 2 – Investigators and Associate Teams 42
Annex 3 – WOMAC Questionnaire Functional Scale 43
Annex 4 – MOS SF-12 Quality of life questionnaire 45
Annex 5 – Quality of life questionnaire: EQ-5D-3L 47
Annex 6 – RAPT (Risk Assessment and Prediction Tool) 49
Annex 12 50
Annex 13 51

SUMMARY

**Title**	Evaluation of an Educational Program Associated With Exercises (EDEX) Before Total Knee Arthroplasty
**Principal Investigator**	Prof. Serge Poiraudeau (Cochin Hospital)
**Research sites**	- Cochin Hospital<br>- Lariboisière Hospital<br>- Clermont-Ferrand University Hospital
**Study duration**	36 months (21-month enrolment period)
**Patient follow-up**	Between 14 and 15 months, depending on the time between the enrolment visit and the intervention for the educational programme combined with an exercise programme
**Study aim**	<u>Primary objective</u>:<br>To assess the effectiveness of a standardised education and exercise programme proposed before a total knee replacement for knee osteoarthritis, in terms of:<br>- ability, at discharge from the surgical department at D4, plus or minus 1 day, to carry out lying-sitting and sitting-standing transfers, walk 30 metres and go up and down one flight of stairs;<br>- speed of functional recovery within the first 6 months (area under the curve of the function subscale of the WOMAC index).<br><br><u>Secondary objectives:</u><br>- to assess the effectiveness of the intervention in terms of pain, function (personalised WOMAC), quality of life, number of steps and treatment satisfaction at 6 and 12 months;<br>- to estimate and compare the cost of the strategies and, where appropriate, carry out a cost-effectiveness analysis.
**Methodology**	A multi-centre, randomised, controlled study
**Number of sites**	3 sites
**Number of patients**	**300 patients will be enrolled:**<br>150 patients in the experimental arm<br>150 patients in the control arm (usual information and advice given by the surgeon)
**Selection criteria**	<u>Inclusion criteria:</u><br>- Men or women aged from 50 to 85 years;<br>- Patients with knee osteoarthritis according to the ACR criteria for which a total knee arthroplasty has been scheduled by the surgeon;<br>- Medical examination carried out in advance, the results of which will be communicated to the patient;<br>- Patients having provided their written informed consent to take part in the study;<br>- Patients affiliated with a social security scheme (beneficiary or entitled party).<br><br><u>Exclusion criteria:</u><br>- Institutionalised patients;<br>- Patients having already undergone ipsilateral total knee arthroplasty;<br>- Patients with chronic inflammatory arthritis;<br>- Cognitive or behavioural disorders making the assessment impossible;<br>- Difficulties in understanding and communicating in French;<br>- TKA for an indication other than knee osteoarthritis.
**Early withdrawal criteria**	- Failure to meet the eligibility criteria<br>- The onset during the study of a condition listed in the exclusion criteria

**Study treatment**	4 group sessions consisting of a therapeutic education session and a physical exercise programme
**Study outline**	- Enrolment visit (V0);<br>- Intervention (education programme combined with an exercise programme);<br>- Preoperative visit;<br>- Surgery department discharge visit (medical record information);<br>- Monthly follow-up by post or email in terms of function (and personalised) subscale of the WOMAC index and pain in the past 48 hours from 6 weeks to 5 months after surgery.<br>Postoperative self-questionnaires at 6 weeks, 6 months and 12 months.
**Primary and secondary endpoints**	Primary endpoints<br>- Ability at discharge: Assessed at D4, plus or minus 1 day. The endpoints to be assessed will be the ability to carry out lying-sitting and sitting-standing transfers, walk 30 metres and go up and down one flight of stairs.<br>- Speed of postoperative functional recovery at 6 months (area under the curve of the function subscale of the WOMAC index).<br><br>Secondary endpoints<br>- Pain assessment (numeric scale), function (personalised WOMAC index), quality of life (MOS SF-12, EQ-5D), number of steps and treatment satisfaction;<br>- Cost comparison between the two strategies from the Health Insurance perspective and from the care system perspective, as a cost-effectiveness ratio.
**Statistical analysis**	An intention-to-treat analysis will be carried out with the data.<br>The statistical analysis will be carried out at the Prof. P. Ravaud Clinical Ethics Centre - Hôtel Dieu hospital, under the responsibility of Dr I. Boutron and G. Baron. SAS software will be used.

I - INTRODUCTION

I.1 - INFORMATION IN THE LITERATURE AND JUSTIFICATION OF THE RESEARCH

Knee osteoarthritis leads to deficiencies in muscle strength, mobility and balance, and a deterioration in cardiopulmonary function, which contribute to changes in patients' functional capacities, affecting gait in particular [Viton, 1999]. It is exacerbated by excess weight and a sedentary lifestyle, and specific education measures are able to reduce these two risk factors [Ravaud 2008]. It is the main cause for a total knee arthroplasty (TKA) being performed. In the United States, 400,000 knee arthroplasties were performed in 2005 and the expected increase in knee osteoarthritis surgery is in the order of 700% by 2030 [Kurtz 2007]. In Canada, from 2004-2005, the performance of TKAs has more than doubled (representing an increase of 124.8%) since 1994-1995 [Canadian Institute for Health Information, 2006]. Patients' functional status and pain level are frequently improved after undergoing TKA, and the pre-TKA physical and functional status (WOMAC) is predictive of postoperative recovery following arthroplasty [Fortin 1999].

The methods of postoperative management are changing. Developments in surgical techniques tend to reduce the length of stay at surgical departments and encourage the direct return home after an elective orthopaedic intervention. Recommendations by the French National Health Authority (HAS) [HAS 2006] and the transition to activity-based pricing (T2A) contribute to shortening the length of hospital admissions to short stays and changing the conditions for admission to a Physical Medicine and Rehabilitation (PMR) facility. Rehabilitation carried out during the preoperative period could enable patients to be better prepared for the intervention, have better functional results and become independent more quickly following the surgery, thereby reducing the length of stay and facilitating the methods of returning home (whether directly or after a stay at a PMR facility). The type of programme required to meet these objectives is still to be defined.

A literature review assessed the benefit of preoperative physiotherapy without multidisciplinary rehabilitation treatment before total knee arthroplasty (TKA) and total hip arthroplasty (THA) [Ackerman 2004]. This review included five randomised, controlled studies: three on the knee, one of which for unicompartmental knee arthroplasty [Weidenheim 1993], and two studies on the hip, from the same patient cohort [Gilbey 2003; Wang 2002]. It concluded the preoperative physiotherapy to be ineffective on the deficiencies and functional incapacity for TKA. However, the studies analysed have significant methodological limitations and most involve a small number of patients, which limits any conclusions being drawn from this analysis.

Educational preoperative interventions have also been assessed. A Cochrane Library review [McDonald 2004] assessed the impact of preoperative education before hip and knee

arthroplasty. This review included nine studies, four of which had length of postoperative stay and functional incapacity as endpoints. Only one study [Crowe 2003] has shown a positive effect in terms of reducing the length of stay without having an impact on functional capacity. This study involved an occupational therapy intervention and proposed personalised management adapted to each patient. This study suggests that the combination of education and exercises could be more effective than a single isolated education measure.

A larger number of studies conducted on osteoarthritis have shown rehabilitation to be beneficial in terms of functional capacities [Fransen 2003]. These studies have allowed for recommendations to be drafted for the practice, recommending exercises for treating knee osteoarthritis. However, the main limitation is that they focus only on medical treatment of lower-limb osteoarthritis before the arthroplasty stage [Hochberg, 1995: 1535, Hochberg 1995: 1541, Pendleton 2000, Roddy 2005, Zhang 2005, Zhang 2006].

The Philadelphia Panel [Philadelphia Panel 2001] and the French Society of Physical Medicine and Rehabilitation, in association with the French Society of Rheumatology and the French Society of Orthopaedic Surgery and Traumatology [Rannou 2007] have taken a pragmatic, multidisciplinary approach combining the systematic analysis of the literature and taking into account practices by differentiating between the pre- and postoperative phase of knee arthroplasty [Coudeyre 2007, Fedmer 2001, Genêt 2007, Thoumie 2007, Barrois 2007, Coudeyre 2007, Lefevre-Colau 2007, Paysant 2007, Froehlig 2008]. The review carried out by the Philadelphia Panel in 2001 [Philadelphia Panel 2001] focussed only on rehabilitation before total knee arthroplasty. It included only one single study [D'Lima 1996] and was unable to reach a conclusion regarding the benefit of the reinforcement exercises. A systematic literature review combined with a practice analysis regarding the benefit of rehabilitation before TKA or THA by a French multidisciplinary panel of experts [Coudeyre 2007] included 10 articles, 3 of which were on TKA and 1 on TKA and THA. The authors concluded that carrying out preoperative rehabilitation before TKA is highly likely to be beneficial, particularly for reducing the length of stay at the surgical department and improving the methods for returning home, and recommended that this rehabilitation should include, as a minimum, exercises combined with educational management. This management would be particularly desirable for very fragile patients with altered functional capacities, comorbidities and/or social issues. The authors highlight that therapeutic trials of good methodological quality which differentiate between TKA and THA are required to support these conclusions.

I.2 - EXPECTED RESULTS AND PROSPECTS

The study will be conducted in patients between the ages of 50 and 85 years with knee osteoarthritis for which total knee arthroplasty has been scheduled by the surgeon. Two patient groups will be studied: one of the two groups will undergo four group sessions including therapeutic education and physical exercises, a knee arthroplasty guide and an exercise CD at least 2 months before the intervention; the other group (control group) will receive the usual information and advice given by the surgeon and according to each centre, and a knee arthroplasty guide.

If this programme is confirmed to be effective, it should lead to a reduction in the length of hospital stay and rate of transfers to rehabilitation care facilities, achieving quicker and more complete functional recovery, improving the patient's quality of life and satisfaction, and reducing treatment costs.

I.3 - STUDY FEASIBILITY

The feasibility of the study is attested by:

- The experience of the clinical teams in the pre- and postoperative management and treatment (particularly treatments including education and physiotherapy) of TKA, with the institutional recognition of the orthopaedic centres ensuring sufficient recruitment;
- Standardisation of the intervention;
- The existence of a cross-functional, complementary steering committee: methodological, logistical and clinical, with an adviser per department, including the principal investigator.

I.4 - EXPECTED BENEFITS AND RISKS

Carrying out a multidisciplinary programme combining education and exercises should lead to quicker and more complete functional recovery, reduce the length of hospital stay and the rates of transfers to rehabilitation care facilities, improving the patient's quality of life and satisfaction, and reducing treatment costs.

The risks are minimal, and are related to the adverse events which may occur during the study.

We have no known or reported factors regarding the risks related to the education programme. The main risks related to the exercise programme are the increase in exercise-related pain and exercise-related injuries to muscles (cramps, elongation), ligaments (sprain) and joints (effusion). If any events occur, the patients will be assessed by the doctor in charge of the study for their treatment. These are minor, non-progressive injuries which are quick to heal, as the exercise intensity will be adapted to the patient's pathology and age, under the supervision of a physiotherapist.

II - STUDY OBJECTIVE

II.1 - PRIMARY OBJECTIVE

The primary objective of this study is to assess the effectiveness of the programme on:

- ability, at discharge from the surgical department at D4, plus or minus 1 day, to carry out lying-sitting and sitting-standing transfers, walk 30 metres and go up and down one flight of stairs;
- speed of postoperative functional recovery within the first 6 months (area under the curve of the function subscale of the WOMAC index).

II.2 - SECONDARY OBJECTIVES

The secondary objectives in these patients with knee osteoarthritis for whom a TKA is scheduled are as follows:

1) Clinical assessment at 6 months and 12 months:

a. participation (personalized WOMAC);
b. pain (visual numeric scale 0-100);
c. quality of life (SF-12);
d. capacities - number of steps (pedometer);
e. treatment satisfaction.

2) Health economics evaluation for the intervention. The costs of the two strategies will be compared at 6 and 12 months by taking into account the perspectives of the payer (Health Insurance) and the healthcare system. In addition, patients' quality of life will be estimated at the enrolment visit, and 6 months and 12 months after the procedure, using the EuroQoL questionnaire (EQ-5D) [Brooks 1996, Fransen 1999] (Annex 5). In the event that the most expensive strategy is the one that provides the best medical outcome and the best quality of life, we will carry out a cost-effectiveness analysis.

Implementing and evaluating complex intervention strategies such as combining educational and supervised exercise programmes is complicated and raises a number of specific methodological issues. Recently published recommendations [Craig 2008] highlight the need to take into account multiple endpoints. Indeed, complex interventions have multiple objectives (improving function, patient information and changing patient behaviour in terms of physical activity and weight-loss).

III - METHODOLOGY

III.1 - STUDY TYPE

This is a randomised, controlled, multi-centre, prospective study.

III.2 - STUDY DURATION

The planned enrolment period is 21 months. The study is therefore expected to last 36 months.

The patients taking part in the study will be monitored for between 14 and 15 months depending on the time elapsed between the enrolment visit and the intervention for the multidisciplinary programme.

The patients will not be able to take part in a biomedical research study with therapeutic assessment likely to change the primary and secondary endpoints of the EDEX study for a duration of 15 months.

Following the study, there is no exclusion period during which participation in another study is prohibited.

III.3 - EXPERIMENTAL DESIGN

We are going to implement a randomised, controlled, multi-centre trial comparing:

1) usual treatment provided by the surgeon;
2) usual treatment provided by the surgeon combined with standardised and personalised multidisciplinary treatment.

The randomised, controlled trial is considered the gold standard for therapeutic assessment.

The study plan, its implementation, analysis and final write up of the results will be carried out in accordance with the CONSORT statement recommendations [Altman *et al.* 2001] and their extensions for non-pharmacological trials [Boutron, Ravaud *et al*. 2008], and the recommendations of the Medical Research Council for developing and evaluating complex interventions [Craig *et al*. 2008].

The patients randomised into the control group will not have any specific treatment apart from the standard treatment proposed by the surgeon and the messages from a knee arthroplasty guide. This choice allows patients to be in the situation which most accurately represents the current clinical practice and takes into account the lack of a standard treatment having proven to be effective which will be used according to consensus for all the sites.

This choice leads to some methodological issues, as blinding of patients, surgeons and the medical and paramedical team responsible for patient management will not be possible. As a result, there will be a risk of bias in performance and assessment. To take into account these risks, we will:

a) Systematically collect the co-interventions prescribed in each group (analgesic treatments, physiotherapy prescription, number of consultations with the surgeon) to assess the risk of contamination and performance bias;

b) Organise an independent assessment by a clinical study technician.

The patients will be informed that the aim of this study will be to compare two treatments for patients in the preoperative period, which are different and possibly effective. To this end, we will explain that they can have either routine care provided by their surgeons, or multidisciplinary care consisting of 4 exercise and therapeutic education sessions while awaiting the surgical intervention, in addition to the routine care.

III.4 - PATIENT ENROLMENT AND RANDOMISATION

Randomisation characteristics

Randomisation will be stratified by site. The randomisation list will be prepared with variable block sizes. The list and block size will not be communicated to the investigators. The secrecy of the assignment will be maintained using an electronic randomisation system.

Enrolment and randomisation procedure

Taking into account the lack of availability of clinicians, and surgeons in particular, patient enrolment in clinical trials is difficult. To compensate for this difficulty, patient screening will be carried out by a clinical study technician.

The clinical study technician will systematically identify all patients who have been scheduled to undergo knee arthroplasty. As agreed with the surgeon in charge of the patient, the clinical study technician will contact the potentially eligible patient and will propose that he or she participate in the EDEX study. If the patient is interested in this study, a consultation will be scheduled, which must take place on the same day as the pre-anaesthesia consultation. During this consultation, the rehabilitation physician or the rheumatologist, assisted by the clinical study technician, will confirm the eligibility criteria, will give the patient the relevant information and, after obtaining the patient’s consent, the patient will be randomised. Patients randomised into the intervention group will be contacted by the clinical study technician to arrange for the intervention to be carried out.

IV - STUDY POPULATION

IV.1 - INCLUSION CRITERIA

- Men or women aged from 50 to 85 years;
- Patients with knee osteoarthritis according to the ACR criteria for which total knee arthroplasty has been scheduled by the surgeon;
- Medical examination carried out in advance, the results of which will be communicated to the patient;
- Patients having provided their written informed consent to take part in the study;
- Patients affiliated with a social security scheme (beneficiary or entitled party).

IV.2 - EXCLUSION CRITERIA

- Institutionalised patients;
- Patients having already undergone ipsilateral total knee arthroplasty;
- Patients with chronic inflammatory arthritis;
- Cognitive or behavioural disorders making the assessment impossible;
- Difficulties in understanding and communicating in French;
- TKA for an indication other than knee osteoarthritis.

IV.3 - REQUIRED NUMBER OF SUBJECTS

A total of 150 patients are planned to be enrolled in the experimental group (multidisciplinary programme) and 150 patients in the control group, i.e. 300 patients in total.

IV.4 - DIAGNOSIS STANDARDISATION

Knee osteoarthritis according to the ACR criteria for which total knee arthroplasty has been scheduled by the surgeon.

IV.5 - RECRUITMENT METHOD

Patients will be recruited from the Orthopaedics, Rheumatology and PMR departments at three centres specialising in the surgical treatment of knee osteoarthritis (Cochin Hospital, Lariboisière Hospital and Clermont-Ferrand University Hospital). Patient recruitment will be done through the usual correspondents, via orthopaedics consultations at the three hospitals. The plan is for the orthopaedists to send potential patients' information to the 3 recruiting sites.
A written information sheet will also be distributed by the sites to free and hospital rheumatologists in the Ile-de-France and Auvergne regions.

Recruitment potential within the sites

280 patients were admitted to the Orthopaedics Department at Cochin Hospital over the past 12 months to undergo a TKA (70% knee osteoarthritis). Over 200 knee arthroplasties are performed each year at the Clermont-Ferrand (90% knee osteoarthritis) and Lariboisière (70% knee osteoarthritis) centres. If we give each of these sites a minimum enrolment hypothesis of 30% of patients in the study, the annual enrolment possibilities may be estimated to be around 160 patients per year.

V - STUDY TREATMENT

V.1 - TREATMENT OUTLINE

As part of this randomised, parallel-group study, 300 patients will be divided into 2 arms (150 patients per arm):

<u>EXPERIMENTAL ARM:</u>

The sessions take place in groups (4 to 6 patients) and include education, performance and teaching of a personalised exercise programme. These sessions begin at least 2 months before the knee arthroplasty is performed.

Supervised session schedule: 2 supervised sessions lasting 1 and a half hours per week over 2 weeks.

Content: Programme preparation and planning are carried out according to the Medical Research Council Guidance recommendations for developing and evaluating complex interventions [Craig *et al.* 2008]. The intervention is developed and defined by a group of experts including orthopaedists, physical medicine and rehabilitation physicians, rheumatologists, physiotherapists, dieticians, psychologists and education science specialists. The group defines the main components of the intervention, its objectives, potential barriers, how to overcome them and how to optimise patient adherence to the messages received.

Therapeutic education sessions (Annex 7): in order to optimise the effect of the messages received, we tested the hypothesis already suggested in other situations (Munjanja SP 1996, Ravaud 2008) that an intervention proposing fewer, but more targeted, messages is more effective than a standardised education programme. To this end, 4 education classes are proposed with very different, simple messages.

In order to improve patient adherence, the first session is dedicated to physical activity before undergoing a TKA and walking after this surgery. The other 3 sessions are based on a single component (orientation at discharge, dietary advice and environment planning). This enables the message to be simplified, better understood by patients and easier to remember. In order to standardise the intervention, training sessions are organised for each of the educational sessions, and group support is provided.

It is made up of four 30-minute sessions before the exercise sessions:

<u>1st session: physical exercise before the arthroplasty.</u>

The role of physical exercise on health and participation, which exercises to do while awaiting surgery, when and how to do them when you return home.

We will confirm that the pedometer is used properly (Annex 10). For active patients, the aim will be to maintain the level of activity. For sedentary patients, the aim will be to gradually increase the number of steps measured using a pedometer by 20% before the surgery. This session is led by a physiotherapist or a sports instructor.

At the end of this session, the physiotherapist will give the patients the self-exercise programme to be done at home.

2nd session:

1st part: Presentation of the criteria for discharge and transfer to the rehabilitation care facilities centre (RAPT table, Annex 6). During this session, we will discuss the patient's opinion regarding their postoperative orientation and how confident they are about returning home.

This session is led by a social worker.

2nd part: Diet

Individual analysis of dietary habits, group reflection on issues such as how to manage weight in the future, how to prevent excessive weight gain after the intervention, possible sources of help, specialist consultations, the psychological consequences of weight gain and management, and the obstacles preventing weight loss. This session will be tailored according to how overweight the patient is, which will be determined according to body mass index (BMI) and their willingness to lose weight. Patients willing to lose weight will be given advice regarding the circuits and the possible treatment methods for achieving this target. Overweight patients who are not very willing to lose this weight will be given advice on how to maintain their current weight.

This session is led by a dietician.

3rd session: preoperative anxiety management.

How to recognise surgery-related anxiety, how to manage it and when to see a doctor. This session is supervised by a psychologist.

4th session: temporary household planning.

When and how to plan your environment How to optimise transfers Presentations of technical aids and advice on how to use them. Individual adaptations of environment and technical aids to facilitate the return home will be discussed based on the description of the patient's environment and lifestyle.

This session is organised by an occupational therapist.

Support materials for these sessions will be in the form of slideshows, pre-TKA exercise CDs and crutches; group discussion and questions. Patients will leave with a knee arthroplasty guide and a self-exercise programme.

Exercise sessions (Annex 8): 4 one-hour sessions after the therapeutic education sessions.

CONTROL ARM:

Usual information and advice given by the orthopaedics department at each centre and knee arthroplasty guide. Patients will also receive a pedometer to measure the number of steps and will learn how to use this on the day of the enrolment visit (Annex 10).

V.2 - ASSOCIATED TREATMENTS

All treatment administered to the patient during the trial must be reported in the case report form.

V.2.1 - AUTHORISED TREATMENTS

The prescription of medicinal products, particularly analgesics and NSAIDs, will be left to the investigator's discretion. Any treatment taken or likely to be taken by the patient during the study must be reported in the self-assessment diary, which will be given to the patient at the enrolment visit.

V.2.2 - PROHIBITED TREATMENTS

There will be no prohibited treatments in the study.

V.3 - PROVISIONS PUT IN PLACE TO COMPENSATE FOR THE LACK OF BLINDING

As the type of treatment proposed does not allow for blinding of the patient or the therapist, various measures will be taken to compensate for the lack of blinding:

- Evaluator blinded to treatments;
- Systematically collect the co-interventions prescribed in each group (analgesic treatments, physiotherapy prescription, number of consultations with the surgeon) to assess the risk of contamination and performance bias.

V.4 - PATIENT CARD

In application of the Good Manufacturing Practice regulations dated 26 May 2006, a patient card will be issued.

VI - ENDPOINTS

VI.1 - PRIMARY ENDPOINTS

The first primary endpoint will be the ability at discharge from the surgical department at D4, plus or minus 1 day. We will assess the four tests described by Zavadak KH *et al*, from D1 to D5 post-surgery:

- transferring from the lying position to the seated position;
- transferring from the seated position to the standing position;
- walking 30 metres;
- going up and down one flight of stairs.

These tests will be performed at four levels of independence:

- level 0: the test cannot be performed

- level 1: the test is performed with physical assistance from a third party
- level 2: the test is performed with verbal assistance from a third party
- level 3: the test is performed without any assistance from a third party

These four tests for measuring the level of dependence in the early postoperative period are the most widely used [Dowsey 1999, Ganz 2003, Kwoh 1997, Munin 1995, Munin 1998, Walker 2001, Zavadak 1995]. The use of sticks or a walker is allowed at all levels. Only intervention from a physical person, whether verbal or mechanical, is considered to be assistance. A patient will be considered to be independent if he or she performs the four tests at level 3. The level of dependency will be assessed every day from D1 to D5 during the patient's hospital stay, by an independent clinical study technician, who is specifically trained and is unaware of the assigned intervention (blinded).

The second primary endpoint will be the assessment of functional recovery 6 months after surgery using the area under the curve of the function subscale of the WOMAC index.

VI.2 - SECONDARY ENDPOINTS

Efficacy criteria

At 6 months and 12 months:

- Knee pain intensity in the past 48 hours (numeric scale),
- Function by the personalized WOMAC scale
- Quality of life (MOS SF-12)
- Number of steps (pedometer).
- Medico-economic analysis: cost estimates and cost-utility analysis
- Treatment satisfaction

-

VII - PATIENT STUDY PLAN

GENERAL OUTLINE OF THE STUDY

Orthopaedists send potential patients' information to the Rehabilitation or Rheumatology departments

↓

V0: Enrolment visit (Day of the pre-anaesthetic consultation)
Collection of consent to take part + Randomisation + Pedometer

↓

Control arm:
Usual information and advice given by the surgeon + knee arthroplasty guide

Experimental arm:
Usual information and advice given by the surgeon
+
4 supervised group sessions
2 months before surgery
(90 minutes, twice/week x 2 weeks):
1/2 hour of education
1 hour of physical exercises + knee arthroplasty guide
+ self-exercise programme + exercise DVD

↓

V1: Preoperative assessment (D0-1 or 2)

↓

D0: Total knee arthroplasty performed

↓

V2: Daily assessment of discharge ability from D1 to D5 during the stay at the surgical department

Follow-up at Week 6, Month 3 (V3), Month 4, Month 5, Month 6 and Month 12 (V4)

	**Enrolment visit**	**Preoperative visit (V1)**	**Surgical discharge visit (V2)**	**Contact at 6 weeks**	**Contact at M3 (V3) (d)**	**Contacts at M4, M5**	**Contact at M6**	**Contact at M12 (V4)**
Information and consent	+							
Randomisation	+							
Weight, height, BMI	+							
WOMAC	+	+		+	+	+	+	+
Numeric pain scale	+	+		+	+	+	+	+
Discharge ability			D1 to D5					
Actual length of stay			+					
Rate of transfer to rehabilitation care facilities			+					
Length of stay at rehabilitation care facilities				+	+		+(a)	
MOS SF 12	+	+			+		+	+
EQ-5D	+						+	+
RAPT	+							
Adaptation to environment		+			+			
Number of steps	+(b)	+(c)			+(c)		+(c)	+(c)
Treatment satisfaction							+	+
Co-interventions	+	+			+		+	+
Adverse Events		+	+		+		+	+

(a) If the length of stay is longer than 6 weeks

(b) To be performed the week after the enrolment visit (7 days)

(c) To be performed the week before the enrolment visit (7 days)

VII.1 - VISIT V0 - ENROLMENT VISIT

The patients selected within the surgical department at Clermont-Ferrand University Hospital and Lariboisière and Cochin hospitals will be contacted by the clinical study technician and, if they are potentially interested in the study, they will be referred to the PMR departments at Cochin and Clermont-Ferrand and the Lariboisière rheumatology department for their enrolment visit and their study follow-up.

At the enrolment visit, the rehabilitation physician or the rheumatologist, with the help of the clinical study technician, will systematically confirm the inclusion and exclusion criteria. The study methods will be clearly explained to the patient. The patient will be given an information sheet and informed consent form. If the patient agrees to take part in the study, he or she will sign the informed consent form.

The following information will be collected in the electronic case report form:

- Demographic parameters (age, sex);
- Socio-professional parameters (level of studies, profession and professional status (working, on sick leave, on disability, retired));
- Medical examination (weight, height and BMI);
- Inclusion and exclusion criteria;
- Information and consent;
- Demonstration of how to use the pedometer to measure the number of steps taken in the week after the enrolment visit;
- Co-interventions (particularly analgesics and/or NSAIDs, physiotherapy).

Patients will fill in a self-assessment card during the visit, including assessment of the following:

- knee pain over the past 48 hours, assessed using a visual numeric scale from 0 to 100;
- preference regarding hospital discharge destination and RAPT scale (Annex 6);
- function according to the WOMAC scale and the personalised section;
- quality of life (MOS SF-12, Annex 4 and EQ-5D, Annex 5).

The time elapsed between the enrolment visit and the start of the multidisciplinary programme will be no more than one month.

Randomisation: patients will be randomised using CleanWeb software.

After randomisation, the patients in the two arms will receive a copy of the Knee Arthroplasty Guide (Annex 11).

The patients randomised into the “standard-treatment” control arm will receive standard medical care.

VII.2 - INTERVENTION PROTOCOL

Patients will be seen at the day hospital (third-category rehabilitation care facilities) on four occasions over two weeks, at least two months before the intervention.

Intervention: The multidisciplinary programmes will be carried out at the Rehabilitation Department at Cochin Hospital and Clermont-Ferrand Hospital and at the Lariboisière Rheumatology Department. The programme is prepared according to the Medical Research Council Guidance recommendations for developing and evaluating complex interventions (Craig *et al.* 2008). This requires expert consensus, preparation of an information booklet for patients, and training for trainers. All investigators have vast experience in treating patients with TKA. The intervention is never carried out by surgeons, which prevents the risk of contamination.

Patients in the intervention group will receive four 1.5-hour supervised group sessions (4 to 6 patients) consisting of 30 minutes of education and 1 hour of physical exercise at least two months before surgery with two sessions per week for two consecutive weeks.

Transfers to the four multidisciplinary treatment sessions will be covered by the study.

Patients in the control group will receive the usual information and advice given by the orthopaedics department at each centre in line with their usual practice and a knee arthroplasty guide (Annex 10), as well as a pedometer to measure the number of steps (Annex 9).

The multidisciplinary programme is standardised and personalised (Annex 7). For the educational part, the four sessions will be divided up as follows:

- The first session led by a physiotherapist deals with the role of physical activity and exercises before undergoing the arthroplasty and walking after the procedure. It will be checked that the pedometer is used properly. For active patients, the aim will be to maintain the level of activity. For sedentary patients, the aim will be to gradually increase the number of steps measured using the pedometer by 20% before the surgery.
- The second session will be led by a social worker and a dietician. The social worker deals with the topic of "information regarding postoperative orientation" according to the patient's characteristics (RAPT table, Annex 6). During this session, the patient will discuss their opinion regarding their postoperative orientation and how confident they are about returning home. The second session deals with the role of excess weight and how to control weight. This session will be tailored to the patient's level of excess weight, which will be determined according to body mass index (BMI) and willingness to lose weight. Patients willing to lose weight will be given advice regarding circuit training and the possible treatments for achieving this target. Overweight patients who are not very willing to lose this weight will be given advice on how to maintain their current weight.

- The third session led by a psychologist deals with issues such as: how to recognise the symptoms of surgery-related anxiety, how to manage anxiety and when to seek professional help.

The fourth session will be led by an occupational therapist. The rules on adapting the environment, identifying accessibility barriers and the main possible technical support tools will be presented to patients. Individual adaptations of environment and technical aids to facilitate the return home will be discussed based on the description of the patient's environment and lifestyle.

For the exercise part (Annex 8), the sessions include muscle-reinforcement exercises for the lower limbs, endurance exercises (aerobic activity), proprioceptive and balance exercises, comprehensive walking exercises and teaching of a personalised exercise programme (Annex 9) to be carried out no more than once per day for 20 to 30 minutes separately from the aerobic exercise (brisk walking at 60% of the theoretical maximum heart rate) to be carried out no more than 3 times per week for 1 hour. The programme will be adjusted according to the patients' preferences and their motivation to carry out the exercise. The maximum programme (30 minutes of exercises per day, brisk walking 3 times per week) will be proposed to the most motivated and most active patients. The minimum recommended programme will consist of three exercise sessions and one period of brisk walking per week. The amount of exercises carried out (observance) will be noted down by the patient in a weekly diary while awaiting the arthroplasty.

Standardisation among the three sites will be double checked before the start of the study.

VII.3 - FOLLOW-UP VISITS AFTER THE INTERVENTION

VII.3.1 - PREOPERATIVE VISIT (V1)

At the preoperative visit, which will take place one or two days before the surgery, the following information will be collected in the electronic case report form:

- Professional status;
- Number of steps measured by the patient in the week (7 days) before this visit;
- Co-interventions since the enrolment visit;
- Adverse events.

Patients will fill in a self-assessment card including assessment of the following:

- knee pain over the past 48 hours (numeric scale);
- function according to the WOMAC scale and the personalised section;
- quality of life (MOS SF-12);

- adaptation of environment.

VII.3.2 - Visits during the stay at the surgical department (V2)

Discharge ability, one of the main endpoints, will be assessed daily by an independent clinical study technician from postoperative D1 until D5 (see section VI-1 of the protocol).

The evaluating physician or clinical study technician will take specific clinical information from the medical file the day before the patient is discharged from the Orthopaedics Department (actual length of stay and adverse events).

After discharge from the Surgical Department, patients will be monitored at consultations, consisting of one visit at 6 weeks, 6 months and 12 months. These two visits will take place as part of the usual follow-up consultations with the surgeon. Follow-up by post or email, according to the patient's preference, will take place at 3, 4 and 5 months. Contacts between the 2 arms for the follow-up visits will be avoided.

VII.3.3 - Visit at 6 weeks after surgery (V3)

The information below will be collected:

- Professional status;
- Number of steps measured by the patient in the week before this visit;
- Length of stay at rehabilitation care facilities;
- Adverse events.

Patients will fill in a self-assessment card including assessment of the following:

- knee pain over the past 48 hours (numeric scale);
- function according to the WOMAC scale and the personalised section;
- quality of life (MOS SF-12);
- adaptation of environment;
- co-interventions since the preoperative visit.

VII.3.4 - Contacts by mail at 3, 4 and 5 months after surgery

During the monthly contacts, the WOMAC functional index and personalised section, as well as pain (numeric scale) over the past 48 hours, will be collected from the patient.

VII.3.5 - Visits at 6 and 12 months after surgery (V4 and V5)

The information below will be collected:

- Professional status;
- Number of steps measured by the patient in the week before this visit;

Patients will fill in a self-assessment card including assessment of the following:

- knee pain over the past 48 hours (numeric scale);
- function according to the WOMAC scale and the personalised section;
- quality of life (MOS SF-12 and EQ-5D);
- Length of stay at rehabilitation care facilities (if > 6 weeks);
- treatment satisfaction;
- co-interventions since the previous visit.

VII.4 - EXITING THE TRIAL AND EARLY WITHDRAWAL

Patients may leave the study at any time and for any reason, if they decide to do so. This will have no consequences on the quality of any subsequent care that the patient will receive. Patients may also be withdrawn from the trial at the investigator’s discretion. All cases of patients withdrawing from the study must be documented.

In the event of early withdrawal, the investigator must fill in the case report form up to the date of early withdrawal. The reason for the early withdrawal must be specified. Patients withdrawing from the trial cannot be enrolled again in the study. The patient’s treatment will continue in the context of routine care.

VII.5 - PROCEDURES FOR LIMITING MISSING DATA

In order to limit missing data, all patients not attending the 6 weeks, 6 months and 12 months follow-up consultations (or those who cannot be reached) will be contacted by telephone or by email. The main aim of these reminders is to reschedule a follow-up consultation. In the event of the patient categorically refusing to attend these consultations, as much information as possible will have to be collected by telephone.

The same approach will be taken for patients who cannot be contacted by post or by email at 3, 4 and 5 months.

VII.6 - SPECIFIC CHARACTERISTICS OF THE STUDY IN RELATION TO THE PATIENT’S USUAL CARE

The specific characteristics of the study are listed in the table below in relation to what is done as part of the patient’s care:

Description of the procedures	CARE	STUDY
Advice given by the surgeon at the orthopaedic consultation	X	
Total knee arthroplasty guide	X	
4 exercise sessions		X
4 therapeutic education sessions		X

Preoperative assessment		X
Daily assessment of discharge ability from D1 to D5		X
Self-exercise programme and CD		X
Follow-up visits at 6 weeks, 6 and 12 months after surgery	X	
Follow-up questionnaires at 3, 4 and 5 months (by post, email or telephone)		X

VII.7 - DESCRIPTION OF THE RULES FOR PARTIAL OR FULL TERMINATION OF THE STUDY

An extraordinary meeting can be convened at the sponsor's request, in the event that any serious adverse events or results which would affect the study occur. The following will also be considered to be reasons to terminate the study:

- insufficient recruitment;
  a. if, one year after the official start date of the study, no enrolments have been made, the DRCD will make the decision to terminate the study early.
  b. if, after 30% of the time planned for enrolling subjects into the study, set from the date of the first enrolment, fewer than 15% of the subjects have been enrolled, a cause analysis must be carried out and corrective actions must be taken (re-motivation of sites, inclusion of additional sites, change in the criteria regarding the population to be enrolled, etc.).
  c. finally, if, after 50% of the provisional time for enrolments as defined above, fewer than 30% of the patients have been enrolled, the DRCD, in agreement with the Steering Committee, can make a decision regarding the definitive termination of enrolments, or even the early termination of the study, depending on the situation.
- the occurrence of excessive SAEs

VIII - QUALITY CONTROL AND ASSURANCE

The study will be conducted in accordance with the sponsor's standard operating procedures. The performance of the study at the research sites and the treatment of subjects will be done in accordance with the Declaration of Helsinki and the Good Clinical Practice guidelines in force.

VIII.1 - MONITORING PROCEDURES

The study is classified as risk level A.
The CRAs representing the sponsor will carry out visits to the research sites according to the follow-up schedule for patients in the protocol, the enrolments at the various research sites and the level of risk assigned to the study.

- Start-up visit at each site: before enrolment, for implementation of the protocol and familiarisation with the various parties involved in the biomedical research study.
- At the next visits, the case report forms will be reviewed as the study progresses by the CRAs. The principal investigator at each site, as well as the other investigators who enrol or undertake follow-up with individuals participating in the study, agree to receive visits from the CRAs at regular intervals. In accordance with the Good Clinical Practice guidelines, the following items will be reviewed at the site visits:

  - Compliance with the protocol and procedures set out for the study;
  - Verification of the patient informed consent forms;
  - Examination of the source documents and comparison with the data reported in the case report form in terms of accuracy, missing data and consistency of the data according to the regulations set out by the procedures of the DRCD.

- Closure visit: collection of case report forms, biomedical research documents, archiving.

VIII.2 - TRANSCRIPTION OF INFORMATION INTO THE CASE REPORT FORM

The study data will be collected using the CleanWeb electronic case report form, within the framework of a public contract between the AP-HP and TELEMEDICINE TECHNOLOGIES S.A., notified on 17/11/2003 (reference no. 033845) and renewed on 26/11/2006 (reference no. 063844). The data will be centralised in a server located at the Operational Services Department (DSO) of AP-HP, 67 boulevard Bessières – 75017 PARIS.

An initial version of the eCRF may be put online and tested after sending the specific study specifications by fax to the company TELEMEDICINE. Once the Coordinator, the Project Leader, the Clinical Research Unit, the Data Manager and the Statistician have agreed on the final version of the eCRF, and following the release of the credits, and submission of the purchase order to the company TELEMEDICINE by the DRCD, the eCRF will come into operation.

In accordance with the Good Clinical Practice guidelines, the case report form in which the study data are transcribed must correspond to at least the following standard presentation:

- At the start of the form, the following are normally included: the title of the study, the patient's study reference, possibly the contact information of the individual taking part in the study (first initials of their surname and first name), randomisation number (where applicable), and inclusion and exclusion criteria in the form of a check-list, which allows subject selection to be validated with respect to the study population. At the end of the study, when the study database has been "frozen", the eCRFs of each patient will be printed and signed by the investigator. The references of the study and of the person taking part in

the study will then appear in the form of a slip on each page to allow data to be identified in all cases.

- The visit and/or sampling dates of the data transcribed must be reported in this eCRF as well as the point in the study to which they correspond.
- Results must contain the units of measurement, and even the laboratory standards in the event that these vary with the technique used.
- The following items must be included at the end of the eCRF:
  - Concomitant treatments;
  - Non-serious adverse events (AEs);
  - End of study/Early termination;
  - Outside planning, an SAE module.

All the information required by the protocol must be provided in the case report form and an explanation must be provided by the investigator for any missing information.

Information must be transferred to the case report forms as soon as it becomes available, whether clinical or paraclinical information.

Incorrect information detected in the case report forms will be replaced in the form by a registered investigator, who will log in to the software with his or her access information (username and password). These codes are strictly personal and confidential, and under no circumstances may be passed on to third parties. They help ensure data confidentiality and authenticate the interventions. Access information is associated with an electronic signature system which validates the data entered by the investigator. Each signature is stamped with the date and time and recorded in the study audit trail. Signed information cannot be changed. However, the investigator may delete his or her signature if he or she wishes to correct any information. Deleting a signature is also subject to stamping with the date and time.

Subject anonymity will be guaranteed by mentioning no more than the number in the study, the initials of the surname and first name of the individual taking part in the study in all documents required for the study, or by erasing any personal information using a suitable method (e.g., correction fluid) from the source documents to be included in the study documentation.

The electronic data file will be declared to the CNIL in accordance with the appropriate procedure for the case.

IX - STATISTICAL DATA MANAGEMENT

IX.1 - RESPONSIBILITY FOR THE DATA ANALYSIS AND SOFTWARE USED

The statistical analysis will be carried out independently by a blinded statistician from the Epidemiology, Biostatistics and Clinical Research Department of the Cochin Hôtel-Dieu Broca Hospital Group, using Statistical Analysis System version 9.1, under the responsibility of Dr. Isabelle Boutron and Prof. Philippe Ravaud. A statistical analysis plan will be prepared and validated prior to the freezing of the database and the unblinding.
The health economics evaluation will be carried out by Prof. Isabelle Durand-Zaleski.

IX.2 - JUSTIFICATION OF THE REQUIRED NUMBER OF SUBJECTS

The first primary endpoint is the percentage of patients able to be discharged from the surgical department on D4, plus or minus 1 day. The expected percentage in the control arm is 20%. The sample size of 130 patients per arm will allow for an odds ratio of 2.15 (logrank test) with a power of 90% for a p-value of 2.5% in bilateral formulation in order to take into account the double endpoint. The second primary endpoint is the WOMAC function area under the curve analysis at 6 months. The total number of 260 subjects enrolled (130 per group) will allow to demonstrate an effect size of 0.45 for comparison of the average areas under the curve with a power equal to 90% (p-value equal to 2.5%, Mann-Whitney test). Taking into account approximately 10% losses-to-follow-up before the procedure, the number of patients to be enrolled into each study arm will be approximately 150, giving a total of 300.

IX.3 - ANALYSIS STRATEGY FOR COLLECTED DATA

An intention-to-treat analysis will be carried out. All randomised patients will be analysed and each patient will be analysed in the group to which they have been randomised, regardless of the technique received.

IX.4 - JUSTIFICATION OF STATISTICAL TESTS

1. Descriptive statistics

The descriptive statistics will be presented in the form of absolute and relative frequencies for the qualitative variables and means with standard deviation and range for the quantitative variables.

2. Primary endpoint analysis

a) The percentage of patients able to be discharged from the surgical department on D4, plus or minus 1 day, will be compared between the 2 groups using the Chi-squared test. Furthermore, analyses adjusted for possible confounding factors (such as patient weight) will also be carried out using a logistic regression model. In addition, an operator effect will be added secondarily to the model to take into account the correlation between the time periods observed in patients

operated on by the same surgeon in the event that there is an operator effect present. The results of the adjusted models will be compared to the non-adjusted model.

b) The WOMAC area under the curve between enrolment and 6 months will be calculated using the trapezoidal rule. The mean areas under the curve will be compared using the non-parametric Mann-Whitney test. In the event of missing data after the initial visit, an area under the curve of 1 will be used.

The endpoint tests will be considered significant at the 2.5% threshold so as to take into account the double primary endpoint.

3. Secondary endpoint analysis

The long-term analysis of the quantitative efficacy criteria repeated over time (personalised WOMAC, numeric pain scale, SF12, number of steps, satisfaction) will be carried out using a linear mixed model for repeated measurements (MMRM) taking into account the correlation of the measurements repeated in the same subject (random effect). The fixed effects will be the randomisation arm, operator, time, initial endpoint value and interaction between the time and the randomisation arm. The model will therefore allow us to compare the means adjusted to the absolute variations between the different times of interest (preoperative, 6 months, 1 year) and the initial visit. This analysis technique is consistent with the principle of the intention-to-treat analysis provided that all patients have a baseline value reported for the endpoint.

4. Health economics evaluation

This is a prospective study in which resource consumption by the patients in each arm will be collected in the case report form and evaluated 1) from the Health Insurance perspective, based on the reference nomenclature and prices in force at the end of the study, and 2) from the care-system perspective using the available hospital accounting data.

The resources collected include:

- time period before the intervention by the surgeons in the control group, and by the teams in charge of education and rehabilitation management in the intervention group;
- length of hospital stay and GHM (or the components of the medical unit summary) corresponding to the stay for the intervention;
- length of stay at rehabilitation care facilities;
- consultations and nursing or physiotherapy procedures during the follow-up period;
- imaging studies and medications consumed during follow-up;
- length of time off work or the time taken to resume normal activity.

There are, a priori, no structure costs related to the intervention as this uses existing capacities. However, we can consider designing a budgetary impact and an impact in terms of therapeutic education professionals if this intervention was to be widely used.

Pricing:

From the Health Insurance perspective: in the control group, the preoperative consultation by the surgeon will be priced at the key-letter rate. The education sessions in the intervention group are not currently priced in the Common Classification of Medical Procedures (CCAM), but we could potentially, by analogy with the pricing of education sessions for diabetic patients, propose 5C for sessions. Admissions to Medical, Surgical and Obstetric (MCO) units will be priced at the GHM rate with any additional supplements (length of stay, mainly). Admissions to rehabilitation care facilities will be priced according to the rates in force at the end of the study, a fixed daily rate or GHM. Other outpatient consumptions will be priced according to the nomenclature rates corresponding to the reference products for medications. With regard to periods of sick leave for individuals who work, these will be priced in line with the average daily allowance amount.

From the care-system perspective: the main difference is that we are seeking to identify which workload transfers between the acute sector (MCO) and the rehabilitation sector would result from the implementation of an intensive preoperative education programme. Indeed, we can speculate that this programme would enable patients to be discharged earlier (which is neutral in terms of cost from the Health Insurance perspective but not from the hospital perspective) and therefore increase the orthopaedic department activity. Based on the analyses carried out by the hospital medical information departments, we can find out if, on average, a department's revenue is higher than its expenditure. It is therefore possible to determine (again, on average) whether or not a one-day reduction in the length of hospital stay would enable the department to generate revenue in relation to increased activity. The rehabilitation care facility perspective is the simplest as the cost of the intervention involved in the study is equal to the salaries of the individuals involved. Given the absence of pricing for education sessions, there is not currently any revenue to compare.

The calculation from the care-system perspective is therefore particularly interesting as it involves both the financial balance within the orthopaedics department and a potential public decision regarding pricing for the rehabilitation care facility sector.

Health economics evaluation.

The intervention costs (from the Health Insurance perspective for the basic calculation) will be compared. In the event that the most expensive strategy is the one which provides the best functional outcome and quality of life, we will carry out a cost-effectiveness analysis. The values will be taken from the EuroQol questionnaire. The base ratio estimation will be completed with a sensitivity analysis (particularly on the pricing of education procedures) and a bootstrap estimation of confidence intervals.

X - SAFETY ASSESSMENT

X.1 - DESCRIPTION OF THE PARAMETERS OF SAFETY ASSESSMENT

- **Adverse event:**

Any harmful manifestation occurring in an individual taking part in a biomedical research study, regardless of whether or not the manifestation is related to the study.

- **Adverse event** in a study not involving a product mentioned in Article L.5311-1 (medicinal products, biomaterials and medical devices, in vitro diagnostic medical devices, labile blood products, organs, tissues, cells and products of human or animal origin, and cellular products for therapeutic purposes).

Any adverse event due to the study.

- **Serious adverse event or effect:**

Any adverse event or effect that leads to death, is life-threatening for the individual taking part in the study, involves hospital admission or an extended hospital stay, causes significant or permanent incapacity or disability, or leads to a congenital abnormality or malformation.

X.2 - PROCEDURE TO MANAGE SERIOUS ADVERSE EVENTS

In the case of biomedical research classified as "risk A", that is to say where the foreseeable risk added by the research is negligible, no serious adverse effects are expected during the research, because:

The study-specific procedures are as follows:

- 4 exercise sessions;
- 4 therapeutic education sessions;
- preoperative assessment;
- daily assessment of discharge ability from D1 to D5;
- self-exercise programme and CD;
- follow-up questionnaires at 3, 4 and 5 months (by post, email or telephone).

This is a multidisciplinary programme combining therapeutic education and exercises, with the exercise intensity being adapted to the patient's pathology and age, under the supervision of a physiotherapist. We have no known or reported factors regarding the risks related to the education programme.

Also, there can be no unexpected serious side effect (SUSAR). In these conditions :

• There is no need for the investigator to notify the sponsor of a serious adverse event occurring during the research. If such events occur, they are related to the pathology or its therapeutic

management within the framework of the care (for example: death related to the pathology, hospitalization or prolongation of hospitalization due to the progression of the disease or to intercurrent illnesses, life-threatening effects unrelated to research, etc.), and are not related to research.

• The classification grid for adverse events will be appended to the protocol. It will only include the list of expected SAEs linked to the pathology or to the usual treatment and which must not be notified to the sponsor. The column of serious adverse events to be notified without delay by the investigator to the sponsor will be marked "none", with some exceptions.

Indeed, in the event that the investigator becomes aware of an event that could jeopardize the safety of any person participating in the research (for example: therapeutic error or deviation from the protocol), he will be required to notify the sponsor. using the form provided for this purpose and annexed to the protocol.

• There is no need to create an independent monitoring committee.

Finally, any non-serious adverse event occurring during the research and which is directly related to it will be notified in the observation log on the adverse events page.

The adverse events that may occur during the study are mainly related to the assigned treatment strategies.

- Main adverse events related to the education programme
  - ✓ no known or reported evidence on this subject
- Main adverse events related to the exercise programme
  - ✓ increase in exercise-related pain
  - ✓ exercise-related injuries to muscles (cramps, elongation), ligaments (sprain) and joints (effusion). These are minor, non-progressive injuries which are quick to heal, as the exercise intensity will be adapted to the patient's pathology and age, under the supervision of a physiotherapist.

If any events occur, the patients will be assessed by the doctor in charge of the study for their treatment.

X.3 - STUDY-SPECIFIC COMMITTEES

X.3.1 - STEERING COMMITTEE

The steering committee will be made up of the Principal Investigator, Prof. Serge Poiraudeau, investigators from the various sites, Dr. Emmanuel Coudeyre, Dr. Pascal Richette, Prof. Philippe Anract, Dr. David Biau, Prof. Rémy Nizard, Dr. Johann Beaudreuil, Prof. Stéphane

Boisgard, the methodologists in charge of the project, Dr. Isabelle Boutron, Prof. Philippe Ravaud, Prof. Isabelle Durand-Zaleski, one or more Project Advisers from the Clinical Research Department (DRCD), the heads of the CIC Cochin Necker Clinical Research Unit (URC), Prof. Jean-Marc Tréluyer, and one or more Project Advisers from the CIC Cochin Necker Clinical Research Unit (URC).

The roles of the steering committee are as follows:

- to define the general organisation and implementation of the study and coordinate the information;
- to initially define the methodology and decide on the measures to be taken throughout the course of the study if any unexpected events occur;
- to supervise the implementation of the study, particularly in terms of tolerance and adverse events.

X.3.2 - INDEPENDENT MONITORING COMMITTEE

The implementation of an independent monitoring committee is not planned for this study. The absence of a monitoring committee is justified by the fact that there is no expected risk.

XI - RIGHT TO ACCESS THE INFORMATION AND SOURCE DOCUMENTS

Individuals with direct access in accordance with the legislative and regulatory provisions in force, in particular Articles L.1121-3 and R.5121-13 of the French Public Health Code (e.g., researchers, individuals in charge of quality control, monitors, clinical research assistants, auditors and all individuals collaborating in clinical trials) will take all the necessary precautions in order to ensure the confidentiality of the information related to the investigational medicinal products, trials, individuals taking part in the study and, in particular, any information involving their identity, as well as the results obtained. The data collected by these individuals through the course of quality controls or audits will then be made anonymous.

XII - LEGAL AND ETHICS ASPECTS

The sponsor is defined by Law 2004-806 of 9 August 2004. AP-HP is the sponsor of this study and the Regional Delegation of Clinical Research (DRCD) undertakes the regulatory tasks.

Before beginning the study, each investigator must provide the sponsor's representative in the study with a signed and dated copy of their curriculum vitae, which must include their French National Medical Council registration number.

XII.1 - REQUEST FOR AUTHORISATION FROM THE AFSSAPS

Before beginning the study, AP-HP, as the sponsor, must submit an authorisation request file to the competent authority (the AFSSAPS). The competent authority, as defined in Article L.1123-12, makes decisions related to the safety of individuals taking part in a biomedical research study, taking into account the safety and quality of the products used during the study in accordance with the regulations in force, where applicable, their condition of use and the safety of individuals with regard to procedures carried out and the methods used, as well as the planned methods of patient follow-up.

XII.2 - REQUEST FOR OPINION FROM THE ETHICS COMMITTEE

In accordance with Article L.1123-6 of the French Public Health Code, the sponsor must submit the study protocol to an Ethics Committee. The committee's opinion will be reported to the competent authority by the sponsor before the study begins.

XII.3 - AMENDMENTS

The DRCD must be informed of any planned changes to the protocol by the principal investigator.

Amendments must be classified as substantial or non-substantial. A substantial amendment is an amendment which may, in one way or another, change the guarantees given to the individuals taking part in the biomedical research (change in inclusion criteria, extension of enrolment period, participation of new sites, etc.).

Once the study has started, any substantial amendments proposed by the sponsor must receive a favourable opinion from the ethics committee and authorisation from the competent authority prior to being implemented. In this case, where necessary, the committee will ensure that a new consent form is duly collected from individuals taking part in the study.

Moreover, any extension to the study (radical change in the treatment regimen or populations included, extension of treatments and/or therapeutic procedures not originally foreseen in the protocol) must be considered as a new study.

Any substantial amendment must be submitted **by the sponsor** for authorisation from the AFSSAPS and/or for the ethics committee's opinion.

XII.4 - CNIL DECLARATION

The law sets forth that the declaration of the electronic file with the personal data collected for the study must be prepared before the effective start of the study.

A reference methodology specific to the processing of personal data carried out in the context of biomedical research studies, defined by Law 2004-806 of 9 August 2004 as falling within

the scope of Articles L.1121-1 et seq. of the French Public Health Code, was established by the CNIL in January 2006. This methodology allows a simplified declaration procedure when the nature of the data collected in the study is consistent with the list provided by the CNIL in its reference document. When the protocol undergoes a quality control of the data by a CRA representing the sponsor and falls within the scope of the simplified CNIL procedure, the DRCD as the sponsor will ask the person in charge of the electronic file to undertake in writing to comply with the simplified MR06001 reference methodology.

XII.5 - INFORMATION SHEET AND INFORMED CONSENT FORM

Written consent must be collected from any individual taking part in the study before any procedures related to the biomedical research are performed.

Within the context of this study, the patients will be enrolled at the visit with the orthopaedist, the rheumatologist or the rehabilitation physician (enrolment visit). During this visit, the patient will be informed of all of the study-related information. At the end of the enrolment visit, if the patient meets all the eligibility criteria and agrees to participate in the study, he or she will provide the physician with the signed consent form.

XII.6 - FINAL STUDY REPORT

The final study report will be prepared by the principal investigator in collaboration with the biostatistician for this study. This report will be submitted to each of the investigators for their opinion. Once a consensus has been reached, the final version must be approved with the signature of each of the investigators and sent to the sponsor as soon as possible after the effective end of the study. A report prepared in accordance with the competent authority reference plan must be sent to the competent authority and to the ethics committee within one year after the end of the study, with the end of the study understood to be the last follow-up visit of the last subject enrolled. This period is set at 90 days if the study is terminated early.

XIII - DATA PROCESSING AND STORAGE OF STUDY-RELATED DOCUMENTS AND DATA

The documents from a study falling under the scope of the law on biomedical research must be archived by all the parties for a period of 15 years after the end of the study (see GCP, chapter 8: essential documents).

This indexed archive consists of:

- Copies of the AFSSAPS authorisation letter and the mandatory opinion from the Ethics Committee;
- Subsequent versions of the protocol (identified by the version number and date);

- Letters of correspondence with the sponsor;
- The informed consent forms signed by the subjects in a sealed envelope (in the case of minor subjects, these are signed by their parents or guardians) with the corresponding list or enrolment register;
- The complete and validated case report form for each subject enrolled;
- Any specific annexes to the study;
- The final study report from the statistical analysis and the quality control of the study (sent in duplicate to the sponsor);
- Certificates from any audits performed during the course of the study.

The database used for the statistical analysis must also be archived by the head analyst (hard copy or electronic copy).

XIV - INSURANCE AND SCIENTIFIC COMMITMENT

XIV.1 - INSURANCE

Assistance Publique - Hôpitaux de Paris is the sponsor of this study. In accordance with the law on biomedical research studies, it has taken out an insurance policy with the company HDI - GERLING for the full duration of the study, guaranteeing its own civil liability as well as that of any intervening parties (physicians or staff involved in conducting the study) (Law no. 2004-806, Art. L.1121-10 of the French Public Health Code).

Assistance Publique - Hôpitaux de Paris reserves the right to interrupt the study at any given time for medical or administrative reasons. If this occurs, the investigator will be notified.

XIV.2 - PRINCIPAL INVESTIGATOR'S COMMITMENT

Each investigator undertakes to comply with the obligations of the law and to conduct the study in accordance with the GCP guidelines, complying with the principles set forth in the Declaration of Helsinki in force. To this end, a copy of the scientific commitment (DRCD document), dated and signed by the principal investigator of each clinical department of a participating site, will be provided to the sponsor's representative.

A task delegation form will be filled in, dated and signed by all study collaborators.

XV - RULES REGARDING PUBLICATION

AP-HP owns the data and it may not be used or transferred to third parties without AP-HP's prior agreement.

The individuals who actively participated in preparing and implementing the protocol, as well as writing up the results, will be named first in the publications.

Assistance Publique-Hôpitaux de Paris must be mentioned as the sponsor of the biomedical research study and as a provider of funding, where applicable. “Assistance Publique-Hôpitaux de Paris” must appear in the address of the authors.

XVI - LIST OF ANNEXES

Annex 1: Literature references

Annex 2: Investigators and Associate Teams

Annex 3: WOMAC Questionnaire Functional Scale

Annex 4: MOS SF-12 Quality of life questionnaire

Annex 5: Quality of life questionnaire: EQ-5D

Annex 6: RAPT (Risk Assessment and Prediction Tool)

Annex 7: Multidisciplinary programme procedure: Therapeutic education

Annex 8: Multidisciplinary programme procedure: Exercise sessions

Annex 9: Pre-TKA self-exercise programme

Annex 10: Pedometer use

Annex 11: Knee arthroplasty guide

Annex 12: Table for Classification of adverse events for a biomedical research study not involving a medicinal product, assimilated product, medical device or genetics

Annex 13: Serious adverse event reporting form

ANNEX 1 – LITERATURE REFERENCES

1. Ackerman IN, Benell KL. Does pre-operative physiotherapy improve outcomes from lower limb joint replacement surgery, A systematic review. Aust J Phys 2004; 50: 25-30.
2. Altman DG, Schulz KF, Moher D, Egger M, Davidoff F, Elbourne D, Gotzsche P, Lang T. The revised CONSORT statement for reporting randomized trials: explanation and elaboration. Ann Inter Med 2001; 134: 663-694.
3. Baecke JAH, Burema J, Frijters JER. A short questionnaire for the measurement of habitual physical activity in epidemiological studies. Am J Clin Nutr, 1982; 36: 936-42.
4. Beaupre L, Lier D, Davies DM, Johnston DBC. The effect of a preoperative exercise and education program on functional recovery, health related quality of life, and health service utilization following primary total knee arthroplasty. J Rheumatol 2004; 31: 1166-73.
5. Boutron I, Moher D, Altman DG, Schulz KF, Ravaud P for the CONSORT Group. Methods and processes of the CONSORT Group: Example of an extension for trials assessing nonpharmacologic treatments. Ann Inter Med 2008; 148: 295-309.
6. Brooks R, the EuroQol Group. EuroQol: the current state of play. Health Policy; 1996; 37: 53-72.
7. Coudeyre E, Jardin C, Givron P, Ribinik P, Revel M, Rannou F. Quel est l'intérêt d'une rééducation avant la pose d'une prothèse totale de hanche ou de genou ? Elaborations de recommandations françaises pour la pratique clinique. Ann Readapt Med Phys; 2007; 50: 189-97.
8. Craig P, Dieppe P, Macintyre S, Mitchie S, Nazareth I, Petticrew M. Developing and evaluating complex interventions: the new Medical Research Council guidance. BMJ 2008; 337: 979-983.
9. Crowe J, Henderson J. Pre-arthroplasty rehabilitation is effective in reducing hospital stay. Can J Occup Ther 2003; 70: 88-96.
10. Dowsey MM, Kilgour ML, Santamaria NM, Choong PF. Clinical pathways in hip and knee arthroplasty: a prospective controlled study. Med J Aust; 1999; 170(2): 59-62
11. D'Lima DD, Colwell CW, Morris BA, Hardwick ME, Kozin F. The Effect of Preoperative Exercise on Total Knee Replacement Outcomes. Clin Orthop Relat Res; 1996; 326: 174-182.
12. Fedmer. Critères de prise en charge en Médecine Physique et Réadaptation Sofmer Bordeaux 2001. Available at http://www.cpod.com/monoweb/fedmer/criteresPEC/index.htm
13. Fortin PR, Clarke AE, Joseph L et al. Outcomes of total hip and knee replacement: preoperative functional status predicts outcomes at six months after surgery. Arthritis Rheum. 1999 ; 42: 1722-8.
14. Fransen M, Edmonds J. Reliability and validity of the EuroQol in patients with osteoarthritis of the knee. Rheumathology; 1999; 38: 807-13.
15. Fransen M, McConnell S, Bell M. Exercise for osteoarthritis of the hip or knee. Cochrane Database Syst Rev. 2003; (3): CD004286.

16. Ganz SB, Wilson PD Jr, Cioppa-Mosca J, Petarson MG. The day of discharge after total hip arthroplasty and the achievement of rehabilitation functional milestones: 11-year trends. J Arthroplasty; 2003; 18(4):453-7.

17. Gilbey HJ, Ackland TR, Wang AW, Morton AR, Trouchet T, Tapper J. Exercise Improves Early Functional Recovery After Total Hip Arthroplasty. Clin Orthop Relat Res 2003; 408; 193-200.

18. HAS. HAS recommendations established by formal consensus, regarding surgical and orthopaedic procedures which do not generally require (for a patient qualifying for massage-physiotherapy treatment) admission for the purpose of providing follow-up care and rehabilitation, as mentioned in Article L.6111-2 of the French Public Health Code. 2006 Paris, France.

19. Hochberg MC, Altman RD, Brandt KD et al. Guidelines for the medical management of osteoarthritis. Part I. Osteoarthritis of the hip. American College of Rheumatology. Arthritis Rheum. 1995 ; 38: 1535-40.

20. Hochberg MC, Altman RD, Brandt KD, Clark BM et al. Guidelines for the medical management of osteoarthritis. Part II. Osteoarthritis of the knee. American College of Rheumatology. Arthritis Rheum. 1995 ; 38: 1541-6.

21. Canadian Institute for Health Information. 2006 report. Hip and knee arthroplasties in Canada. Canadian Joint Replacement Registry (CJRR). Canadian Institute for Health Information, Ontario, 2006, pp 12, 25.

22. Kraaimaat FW, Evers AWM. Pain-coping strategies in chronic pain patients: psychometric characteristics of the Pain-Coping Inventory (PCI). Int J Behav Med 2003; 10: 343-63.

23. Kurtz SM, Ong KL, Lau E, Mowat F, Halpern M. Projections of primary and revision hip and knee arthroplasty in the United States from 2005 to 2030. J Bone Joint Surg Am. 2007;89(4): 780-5

24. Kwoh CK, Petrick MA, Munin MC. Inter-rater reliability for function and strength measurements in the acute care hospital after elective hip and knee arthroplasty. Arthritis Care Res; 1997; 10(2); 128-34.

25. Mathias S, Nayak US, Isaacs B. Balance in elderly patients: the “get up and go” test. Arch Phys Med Rehabil; 1986; 67: 387-89.

26. McDonald S, Green SE, Hetrick S. Pre-operative education for hip or knee replacement. The Cochrane Database of Systematic Reviews 2004, Issue 1. Art. No.: CD003526.

27. Mitchell C, Walker J, Walters S, Morgan AB, Binns T, Mathers N. Costs and effectiveness of pre- and post-operative home physiotherapy for total knee replacement: randomized controlled trial. J Eval Clin Pract. 2005; 11: 283-92.

28. Mouthon L, Rannou F, Bérezné A, Pagnoux C, Guilpain P, Goldwasser F, Revel M, Guillevin L, Fermanian J, Poiraudeau S. Patient preference disability questionnaire in systemic sclerosis: a cross-sectional survey. Arthritis Rheum 2008; 59: 968-73.

29. Munin MC, Rudy TE, Glynn NW, Crossett LS, Rubash HE. Predicting discharge outcome after elective hip and knee arthroplasty. Am J Phys Med Rehabil; 1995; 74(4): 294-301

30. Munin MC, Rudy TE, Glynn NW, Crossett LS, Rubash HE. Early inpatient rehabilitation after elective hip and knee arthroplasty. Jama; 1998; 279(11):847-52

31. Munjanja SP Lindmark G, Nyström L Randomized controlled trial of a reduced-visits programme of antenatal care in Harare, Zimbabwe, Lancet 1996, 348:364-369

32. Pendleton A, Arden N, Dougados M, Doherty M et al. EULAR recommendations for the management of knee osteoarthritis: report of a task force of the Standing Committee for International Clinical Studies Including Therapeutic Trials (ESCISIT). Ann Rheum Dis; 2000; 59: 936-44.

33. Pereira MA, FitzerGerald SJ, Gregg EW, Joswiak ML, Suminski RR, Utter AC, Zmuda JM. A collection of physical activity questionnaires for health-related research. Med Sci Sports Exerc. 1997; 29 (6 suppl): S1-205.

34. Perrot S, Poiraudeau S, Kabir M, Bertin P, Sichere P, Serrie A, Rannou F. Active or passive pain coping strategies in hip and knee osteoarthritis? Results of a national survey of 4,719 patients in a primary care setting. Arthritis Rheum; 2008; 59: 1555-62.

35. Philadelphia Panel. Philadelphia Panel evidence-based clinical practice guidelines on selected rehabilitation interventions for knee pain. Phys Ther; 2001; 81: 1675-1700.

36. Philippaerts RM, Westerterp KR, Lefevre J. Doubly labelled water validation of three physical activity questionnaires. Int J Sports Med; 1999; 20: 284-89.

37. Podsiadlo D, Richardson S. The timed “Up & Go”: a test of basic functional mobility for frail elderly persons. J Am Geriatr Soc; 1991; 39: 142-48.

38. Ravaud P, Flipo R-M, Boutron I, Roy C, Mahmoudi A, Giraudeau B, Pham T. The ARTIST (Osteoarthritis Intervention Standardized) study: A pragmatic randomised controlled trial comparing standardized consultation to usual care for patients with knee osteoarthritis under primary care. BMJ 2008 in Press.

39. Roddy E, Zhang W, Doherty M et al. Evidence-based recommendations for the role of exercise in the management of osteoarthritis of the hip or knee - the MOVE consensus. Rheumatology (Oxford) 2005; 44: 67-73.

40. Seror R, Tubach F, Baron G, Falissard B, Logeart I, Dougados M, Ravaud P. Individualising the Western Ontario and McMaster Universities osteoarthritis index (WOMAC) function subscale: incorporating patient priorities for improvement to measure functional impairment in hip or knee osteoarthritis. Ann Rheum Dis; 2008; 67: 494-99.

41. Tugwell P, Bombardier C, Buchanan WW, Goldsmith CH, Grace E, Hanna B. The MACTAR patient preference disability questionnaire – an individualized functional priority approach for assessing improvement in physical disability in clinical trials in rheumatoid arthritis. J Rheumatol 1987; 14: 446-51.

42. Verhoeven AC, Boers M, Van Der Liden S. Validity of the MACTAR questionnaire as a functional index in a rheumatoid arthritis clinical trial. The McMaster Toronto Arthritis. J Rheumatol 2000; 27: 2801-9.

43. Viton JM, Atlani L, Mesure S, Franceschi JP, Massion J, Delarque A, Bardot A. Reorganization of equilibrium and movement control strategies in patients with knee arthritis. Scand J Rehabil Med. 1999; 31: 43-8.

44. Walker WC, Keyser-Marcus LA, Cifu DX, Chaudhri M. Inpatient interdisciplinary rehabilitation after total hip arthroplasty surgery: a comparison of revision and primary total hip arthroplasty. Arch Phys Med Rehabil; 2001; 82(1): 129-33.

45. Wang AW, Gilbey HJ, Ackland TR. Perioperative exercise programs improve early return of ambulatory function after total hip arthroplasty: A randomized, controlled trial. Am J Phys Med Rehabil 2002; 81: 801-806.

46. Weidenheim L, Mattsson E, Brostrom LA, Wersallbrobertsson E. Effect of pre-operative physiotherapy in unicompartmental prosthetic knee replacement. Scand J Rehabil Med 1993; 25: 33-39.

47. Zavadak KH, Gibson KR, Whitley DM, Britz P, Kwoh CK. Variability in the attainment of functional milestones during the acute care admission after total joint replacement. J Rheumatol; 1995; 22(3): 482-7.

48. Zhang W, Doherty M, Arden N et al. EULAR evidence based recommendations for the management of hip osteoarthritis: report of a task force of the EULAR Standing Committee for International Clinical Studies Including Therapeutics (ESCISIT). Ann Rheum Dis. 2005 ; 64: 669-81.

49. Zhang W, Doherty M. EULAR recommendations for knee and hip osteoarthritis: a critique of the methodology. Br J Sports Med 2006; 40: 664-9.

ANNEX 2 – INVESTIGATORS AND ASSOCIATE TEAMS

Site no.	Hospital and Department	Principal Investigators
01	**Cochin Hospital**<br>27 rue du Faubourg St Jacques<br>75679 Paris Cedex 14<br><br>**Department of Physical Medicine and Rehabilitation of the Musculoskeletal System and Spinal Disorders (PMR)**	**Prof. Serge Poiraudeau**
02	**Clermont-Ferrand University Hospital**<br>Bd Leon Malfreyt<br>63058 Clermont-Ferrand France<br><br>**Rehabilitation Department**	**Prof. Emmanuel Coudeyre**<br>(Rehabilitation Specialist)
03	**Lariboisière Hospital**<br>2 rue Ambroise Paré<br>75010 Paris<br><br>**Rheumatology Department**	**Prof. Pascal Richette**<br>(Rheumatologist)

ANNEX 3 – WOMAC QUESTIONNAIRE FUNCTIONAL SCALE

Function Section

For each of the following activities, please specify the difficulties experienced due to the affected joint, over the past 48 hours.

**How significant is the difficulty you experienced when:**	**None**<br>**0**	**Minimal**<br>**1**	**Moderate**<br>**2**	**Severe**<br>**3**	**Very severe**<br>**4**
1. Going down stairs	❑	❑	❑	❑	❑
2. Going up stairs	❑	❑	❑	❑	❑
3. Standing up from the seated position	❑	❑	❑	❑	❑
4. Standing	❑	❑	❑	❑	❑
5. Leaning forward	❑	❑	❑	❑	❑
6. Walking on level ground	❑	❑	❑	❑	❑
7. Getting in and out of the car	❑	❑	❑	❑	❑
8. Doing the shopping	❑	❑	❑	❑	❑
9. Putting on tights or socks	❑	❑	❑	❑	❑
10. Getting out of bed	❑	❑	❑	❑	❑
11. Taking off tights or socks	❑	❑	❑	❑	❑
12. Lying down in bed	❑	❑	❑	❑	❑
13. Getting in or out of the bath	❑	❑	❑	❑	❑
14. Sitting down	❑	❑	❑	❑	❑
15. Sitting down on and standing up from the toilet	❑	❑	❑	❑	❑
16. Cleaning the house	❑	❑	❑	❑	❑
17. Carrying out daily housework	❑	❑	❑	❑	❑

Calculation = (sum/17) x 25

Total 0-100 = |__|__| |__|__| |__|__|

Personalised WOMAC Section

Please choose (from the 17 items in the previous list) the 5 most important items for which you would like to see an improvement.

1.- ………………………………………………………………
2.- ………………………………………………………………
3.- ………………………………………………………………
4.- ………………………………………………………………
5.- ………………………………………………………………

Calculation = (sum/5) x 25

Total 0-100 = |_|_|_|_|_|_| |_|_|

ANNEX 4 – MOS SF-12 QUALITY OF LIFE QUESTIONNAIRE

The following questions ask for your views about your health. Your answers will help monitor your health condition and to know how well you are able to carry out your usual activities.
Answer all of the following questions by following the instructions you have been given. If you are unsure, please give the best answer you can.

1. **In general, would you say your health is:** *(mark one answer only)*

   - Excellent .... ☐
   - Very good .... ☐
   - Good .... ☐
   - Fair .... ☐
   - Poor .... ☐

The following is a list of activities you might do during a typical day. For each of these, indicate whether your current health condition limits you in these activities.

(mark one answer only per line)

	Very limited	Somewhat limited	Not at all limited
2. **Moderate physical activities** such as moving a table, pushing a vacuum cleaner, bowling	☐	☐	☐
3. Climbing **several flights** of stairs	☐	☐	☐

During the <u>past 4 weeks,</u> due to your <u>physical condition</u>,

(mark one answer only per line)

	YES	NO
4. Have you **accomplished less** than you would have liked?	☐	☐
5. Have you had any **difficulties** in doing your job or any other activities (e.g. they have required an additional effort)?	☐	☐

During the <u>past 4 weeks</u>, due to your <u>emotional state</u> (such as feeling sad, nervous or depressed),

(mark one answer only per line)

	YES	NO
6. Have you **accomplished less** than you would have liked?	☐	☐
7. Have you had **difficulties** in doing what you had to do **with as much care and attention as usual?**	☐	☐

8. **During the past 4 weeks, how much did your physical pain interfere with your work or housework?**

(mark one answer only)

-Not at all .... ☐
-A little bit .... ☐
-Moderately .... ☐
-A lot .... ☐
-Extremely ……………….................….. ☐

The following questions are related to how you have felt <u>during the past 4 weeks.</u> For each question, please indicate the response you feel is most appropriate.

<u>During the past 4 weeks,</u> have there been times when:-

(mark one answer only per line)

	All of the time	Most of the time	A good bit of the time	Some of the time	A little of the time	None of the time
9. You have felt calm and relaxed?	☐	☐	☐	☐	☐	☐
10. You have felt full of energy?	☐	☐	☐	☐	☐	☐
11. You have felt sad and discouraged?	☐	☐	☐	☐	☐	☐

12. **<u>During the past 4 weeks</u>, how much of the time has your <u>physical health or emotional problems</u> interfered with your social activities and relationships with others (family, friends, etc.)?**

(mark one answer only)

-All of the time.. ☐
-Most of the time .... ☐
-From time to time .... ☐
-Rarely .... ☐
-Never .... ☐

ANNEX 5 – QUALITY OF LIFE QUESTIONNAIRE: EQ-5D-3L

Please indicate, for each of the following sections, which statement best describes your health today, by ticking the corresponding box.

Mobility

I have no problems in walking about ☐

I have some problems in walking about ☐

I am confined to bed ☐

Self-care

I have no problems with self-care ☐

I have some problems washing or dressing myself ☐

I am unable to wash or dress myself ☐

Usual activities (*e.g. work, study, housework, family or leisure activities)*

I have no problems with performing my usual activities ☐

I have some problems with performing my usual activities ☐

I am unable to perform my usual activities ☐

Pain/Discomfort

I have no pain or discomfort ☐

I have moderate pain or discomfort ☐

I have extreme pain or discomfort ☐

Anxiety/Depression

I am not anxious or depressed ☐

I moderately anxious or depressed ☐

I am extremely anxious or depressed ☐

Best health condition
imaginable

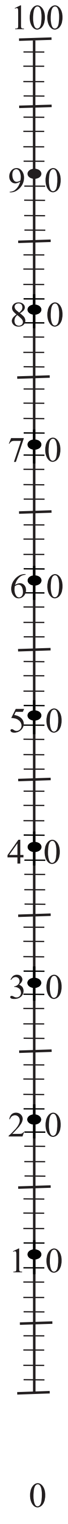

Worst health condition
imaginable

To help you indicate to what extent a health condition is good or bad, we have created a graduated scale (like a thermometer) on which 100 corresponds to the best health condition you can imagine and 0 corresponds to the worst health condition you can imagine.

Please indicate on this scale where you consider your health condition to be today. To do this, please draw a line from the box below to the point on the scale which corresponds to your health condition today.

Your health condition today

ANNEX 6 – RAPT (RISK ASSESSMENT AND PREDICTION TOOL)

RAPT index

	Data	Score
1. How old are you?	50-65 years old<br>66-75 years old<br>> 75 years old	*=2*<br>*=1*<br>*=0*
2. What is your sex?	Male<br>Female	*=2*<br>*=1*
3. How far on average can you walk?	More than 400 metres (with or without rest)<br>200 to 400 metres<br>Housebound (most of the time)	*=2*<br>*=1*<br>*=0*
4. Do you use a walking aid (more often than not)?	None<br>Single-point stick<br>Crutches or a frame	*=2*<br>*=1*<br>*=0*
5. Do you have external help?<br>(home help, meals on wheels, district nursing)	Never or once per week<br>Two or more times per week	*=1*<br>*=0*
6. Will you live with someone who can help you after your operation?	Yes<br>No	*=3*<br>*=0*
Total score out of 12		=

ANALYSIS

Destination at discharge from surgical department according to score.
Score < 6: Transfer to a Rehabilitation Care Facility
Score 6–9: Direct return home subject to additional perioperative care.
Score > 9: Direct return home

The patient's preference regarding orientation is also a crucial factor.
The orientation predicted by the score is discussed with the patient to decide on a discharge plan.

**Patient preference**	**Predictive score**	**Final decision**
…………………………..	……………………………..	……………………………..

ANNEX 12

Grille de Notification des Evénements Indésirables pour une Recherche Biomédicale
ne portant pas sur un produit de santé (Art. R. 1123-54 du Code de la Santé publique)

ASSISTANCE PUBLIQUE HÔPITAUX DE PARIS

EDEX Codes projet : P100122 – IDRCB 2012-A-00208-35 Risque de la Recherche : **A** CSI / DSMB : Oui ☐ **Non** ☒

« Evaluation d'un programme d'éducation associé à un programme d'exercices (EDEX) avant mise en place d'une prothèse totale de genou »

**A NE PAS NOTIFIER** AU PROMOTEUR<br>**Evénements recensés dans le protocole comme ne devant pas être notifiés mais qui pourront être recueillis dans le cahier d'observation (CRF)**		**A NOTIFIER SANS DELAI** AU PROMOTEUR<br>**Envoi du formulaire de notification d'EIG par fax au 01 44 84 17 99 et à recueillir dans le CRF**	
Événements pouvant être graves **mais non liés aux actes et procédures ajoutés spécifiquement par cette recherche**	**Effets Indésirables (EI) Non Graves ATTENDUS**<br>Connus pour être liés aux actes et procédures ajoutés spécifiquement par cette recherche	**Effets Indésirables Graves (EIG) ATTENDUS**	**Effets Indésirables Graves (EIG) INATTENDUS (SUSARs)**
<u>Description</u> :<br>➢ Tout ce qui est en rapport avec l'évolution naturelle et habituelle de la pathologie de la gonarthrose :<br>• aggravation de la maladie<br>• hospitalisation programmée ou non pour suivi de la pathologie<br>➢ Tout effet indésirable grave susceptible d'être lié aux traitements prescrits dans le cadre du soin pendant le suivi de la recherche<br>➢ Tout autre évènement médical en dehors de la pathologie de la gonarthrose, non lié aux actes et procédures ajoutés spécifiquement par cette recherche	<u>Description</u> :<br>➢ Liés au programme d'éducation thérapeutique :<br>• pas d'éléments connus ou rapportés<br>➢ Liés au programme d'exercices :<br>• augmentation des douleurs<br>• lésions musculaires (crampes, élongations), ligamentaires (entorses) et articulaires (épanchement) en rapport avec les exercices<br>Il s'agit de lésions bénignes, non évolutives avec cicatrisation rapide, car l'intensité de l'exercice sera adaptée à la pathologie et âge des patients, sous la supervision d'un kinésithérapeute. Devant la présence de ces événements, les patients seront évalués par le médecin responsable de l'étude pour leur prise en charge.	**Aucun effet indésirable grave n'est attendu dans le cadre de cette recherche.**<br>**NEANT**	**Seuls les EIG directement liés aux actes et procédures ajoutés spécifiquement par cette recherche sont à notifier au promoteur, <u>sans délai à compter du jour où l'investigateur en a connaissance</u>.**

Nom, prénom et signature de l'investigateur coordonnateur :	Nom, prénom et signature du responsable de l'URC :	Nom, prénom et signature du référent projet :	Nom, prénom et signature du responsable Vigilance :	Nom, prénom et signature du coordonnateur médical :
Date :	Date :	Date :	Date :	Date :

10SPU-Edex_grille-vigilance_v2-0_20140331_DRCD

FORMULAIRE DE DECLARATION D'UN EVENEMENT INDESIRABLE GRAVE (EIG) SURVENANT AU COURS D'UNE RECHERCHE BIOMEDICALE NE PORTANT PAS SUR UN PRODUIT MENTIONNE A L'ARTICLE L.5311-1 DU CODE DE LA SANTE PUBLIQUE

ASSISTANCE PUBLIQUE HÔPITAUX DE PARIS

PARTIE RESERVEE AU PROMOTEUR : NE PAS REMPLIR
|_|_|_|-|_|_|_|- *DRCD* -|_|_|_|_|_|-|_|_|_|_|_|

Cette fiche doit être retournée dûment complétée (2 pages) au DRCD par fax : +33 (0)1 44 84 17 99
A l'attention de « *Moufida Dabbech* » et de « *Karine Goude* »

Date de notification : |__|__| jj |__|__| mm |__|__|__|__| aaaa

Code de la Recherche : P100122

N°IDRCB : 2012-A-00208-35 **Déclaration initiale** ☐ **Suivi d'EIG déclaré** ☐ **N°du suivi** |__|

Titre de la Recherche Biomédicale : *« Evaluation d'un programme d'éducation associé à un programme d'exercices (EDEX) avant mise en place d'une prothèse totale de genou »*

1) Nom et adresse du centre : ____________________

Centre n° : |__|__| Investigateur (Qualité - Nom - Prénom) : ____________________

2) Identification du patient :

Nom : |__| Prénom : |__|

Patient n° : |__|__|__|__|

Sexe : Masculin ☐ Féminin ☐

Date de naissance : |__|__| |__|__| |__|__|__|__|

Age : |__|__| ans

Poids : |__|__|__| kg

Taille : |__|__|__| cm

Date d'inclusion : |__|__| |__|__| |__|__|__|__|

Date de randomisation : |__|__| |__|__| |__|__|__|__|

Bras __________ ☐ Bras __________ ☐

3) Evénement indésirable grave :

Décès ☐

Mise en jeu du pronostic vital ☐

Nécessite ou prolonge l'hospitalisation : ☐
Du |__|__|__|__|__|__|__|__| *au* |__|__|__|__|__|__|__|__| ☐ *en cours*

Incapacité ou invalidité ☐

Anomalie congénitale ☐

Autre(s) critère(s) médicalement significatif(s) (préciser) : ☐

____

____

Antécédents (allergie, insuffisance rénale ...) : ____________________

____

4) Description complète de l'événement indésirable (diagnostic retenu, localisation anatomique, critères permettant de considérer l'événement comme grave) :

Intensité : Légère ☐ Modérée ☐ Sévère ☐

Date de survenue : |__|__| jj |__|__| mm |__|__|__|__| aaaa et heure de survenue : |__|__| hh |__|__| min

____

____

Délai de survenue : ____________________

5) Médicament(s) concomitant(s) à l'exclusion de ceux utilisés pour traiter l'événement indésirable :

Nom commercial (de préférence) ou Dénomination Commune Internationale	Voie	Dose/ 24h	Date de début	En cours	Date de fin	Indication	Causalité* (1,2,3 ou 4)
			|__|__| |__|__| |__|__|	☐	|__|__| |__|__| |__|__|		
			|__|__| |__|__| |__|__|	☐	|__|__| |__|__| |__|__|		
			|__|__| |__|__| |__|__|	☐	|__|__| |__|__| |__|__|		
			|__|__| |__|__| |__|__|	☐	|__|__| |__|__| |__|__|		

** 1 = Probable 2 = Possible 3 = Non liée 4 = Inconnue*

Identification du patient : Nom |__| Prénom : |__| Centre : |__|__| Code de la Recherche : P100122

6) Description du dispositif médical (DM) concomitant :

Nature	Modèle /type/référence	N° de série ou de lot	Nom du fournisseur	Nom du fabricant	Si DM stérile : Date de stérilisation et Date de péremption	Causalité* (1,2,3 ou 4)
					|__|__| |__|__| |__|__| et |__|__| |__|__| |__|__|	

* 1 = Probable 2 = Possible 3 = Non liée 4 = Inconnue

7) Evolution (indiquez si des mesures symptomatiques ont été prises : non ☐ oui ☐ *Si oui, préciser*) :

____

8) Date de disparition : |__|__| jj |__|__| mm |__|__|__|__| aaaa et heure de disparition : |__|__| hh |__|__| min

9) Autre(s) étiologie(s) envisagée(s) : non ☐ oui ☐ *Si oui, préciser :*

____

10) Examen(s) complémentaire(s) réalisé(s) : non ☐ oui ☐ *Si oui, préciser date, nature et résultats :*

____

11) Selon l'investigateur, l'événement indésirable grave semble plutôt lié :

☐ à un dispositif médical posé
☐ à un (ou plusieurs) médicament(s) administré(s) : le(s)quel(s) : ____________________
☐ aux procédures de la recherche biomédicale
☐ à une maladie intercurrente
☐ à la progression de la maladie
☐ autre : ____________________

Date : |__|__||__|__||__|__| **Tampon du service :** **Nom de l'investigateur:** ______________ **Signature :**

Nom et fonction du Notificateur: ________________________ **Téléphone** ________________ **Signature :**

PARTIE RESERVEE AU PROMOTEUR : NE PAS REMPLIR

***Numéro d'identification de l'événement :* EV | | | |**

Date de réception par le promoteur : | | | | | | | | | | | |

Date de ce rapport : |__|__| |__|__| |__|__|__|__| ☐ initial ☐ suivi n°|__|__|

Selon le promoteur, l'événement indésirable semble plutôt lié :

☐ à un dispositif médical posé
☐ à un (ou plusieurs) médicament(s) administré(s) : le(s)quel(s) :
☐ aux procédures de la recherche biomédicale
☐ à une maladie intercurrente
☐ à la progression de la maladie
☐ autre :

Si selon le promoteur, l'événement semble plutôt lié à la recherche biomédicale :

☐ L'événement indésirable grave est attendu ☐ L'événement indésirable grave est inattendu

Commentaires du promoteur : ____

Nom et qualité du représentant du promoteur : **Signature :**

ASSISTANCE PUBLIQUE HÔPITAUX DE PARIS

EVALUATION OF AN EDUCATIONAL PROGRAM ASSOCIATED WITH EXERCISES (EDEX) BEFORE TOTAL KNEE ARTHROPLASTY

# - EDEX -

VERSION no. 6.0 dated 07/02/2018

**Sponsor:**	Assistance Publique des Hôpitaux de Paris<br>Delegation of Clinical Research and Innovation (DRCI)<br>Carré Historique – Hôpital Saint-Louis<br>1, avenue Claude Vellefaux<br>75010 Paris<br>Project Leader: Karine Goude-Ory<br>Tel.: (+33) 01 44 84 17 22 / Fax: (+33) 01 44 84 17 01
**Principal Investigator:**	Prof. François Rannou<br>Department of Physical Medicine and Rehabilitation of the Musculoskeletal System and Spinal Disorders<br>Hôpital Cochin,<br>27 rue du Faubourg St Jacques<br>75679 PARIS Cedex 14<br>Tel.: (+33) 01 58 41 25 35 / Fax: (+33) 01 58 41 25 45<br>Email: francois.rannou@aphp.fr
**Methodology and statistical analysis:**	Prof. Isabelle Boutron, Prof. Philippe Ravaud,<br>Clinical Epidemiology Centre<br>Hôpital Hôtel Dieu<br>1, place du Parvis Notre Dame 75004 Paris<br>Tel.: (+33) 01 40 25 79 43
**Health economics evaluation:**	Prof. Isabelle Durand-Zaleski<br>URC Eco Ile de France<br>GH Albert Chenevier-Henri Mondor<br>51 avenue du Maréchal de Lattre de Tassigny<br>94010 Créteil
**Clinical Research Unit:**	CRU Cochin - Necker<br>Site Tarnier<br>89 rue d’Assas<br>75006 PARIS<br>Tel.: (+33) 01 58 41 28 84 / Fax: (+33) 01 58 41 11 83

Biomedical research PROTOCOL SIGNATURE page
for the PRINCIPAL investigator and the SPONSOR's representative

Biomedical research study no. 100122 *code:* ***EDEX AOM 10042***

ANSM registration number: **2012-A00208-35**

Title: "Evaluation of an Educational Program Associated With Exercises (EDEX) Before Total Knee Arthroplasty"

Version no. 6.0 dated 07/02/2018

Principal Investigator:

Prof. François Rannou

Department of Physical Medicine and Rehabilitation of the Musculoskeletal System and Spinal Disorders
Hôpital Cochin,
27 rue du Faubourg St Jacques
75679 PARIS Cedex 14

Date:

Signature:

Sponsor:

Assistance Publique – Hôpitaux de Paris
Delegation of Clinical Research and Innovation
Hôpital Saint Louis
75010 PARIS

Date: …………../………/………..

Signature:

NB: This version corresponds to the text from the protocol and annexes sent to the Ethics Committee (CPP) for its opinion and to the competent authority for authorisation.

TABLE OF CONTENTS

Summary 5

I - INTRODUCTION 7

I.1 - INFORMATION IN THE LITERATURE AND JUSTIFICATION OF THE RESEARCH 7
I.2 - EXPECTED RESULTS AND PROSPECTS 9
I.3 - STUDY FEASIBILITY 9
I.4 - EXPECTED BENEFITS AND RISKS 9

II - STUDY OBJECTIVE 10

II.1 - PRIMARY OBJECTIVE 10
II.2 - SECONDARY OBJECTIVES 10

III - METHODOLOGY 10

III.1 - STUDY TYPE 10
III.2 - STUDY DURATION 11
III.3 - EXPERIMENTAL DESIGN 11
III.4 - PATIENT ENROLMENT AND RANDOMISATION 12

IV - STUDY POPULATION 12

IV.1 - INCLUSION CRITERIA 12
IV.2 - EXCLUSION CRITERIA 13
IV.3 - REQUIRED NUMBER OF SUBJECTS 13
IV.4 - DIAGNOSIS STANDARDISATION 13
IV.5 - RECRUITMENT METHOD 13

V - STUDY TREATMENT 13

V.1 - TREATMENT OUTLINE 13
V.2 - ASSOCIATED TREATMENTS 16
V.2.1 - Authorised treatments 16
V.2.2 - Prohibited treatments 16
V.3 - PROVISIONS PUT IN PLACE TO COMPENSATE FOR THE LACK OF BLINDING 16
V.4 - PATIENT CARD 16

VI - ENDPOINTS 16

VI.1 - PRIMARY ENDPOINTS 16
VI.2 - SECONDARY ENDPOINTS 17

VII - PATIENT STUDY PLAN 18

General outline of the study 18
VII.1 - VISIT V0 - ENROLMENT VISIT 19
VII.2 - INTERVENTION PROTOCOL 20
VII.3 - FOLLOW-UP VISITS AFTER THE INTERVENTION 21
VII.3.1 - Preoperative visit (V1) 21
VII.3.2 - Visits during the stay at the surgical department (V2) 22
VII.3.3 - Contact by post or email 6 weeks after surgery 22
VII.3.4 - Contact 3 months after surgery (V2) 22
VII.3.5 - Contact by post or email 4 and 5 months after surgery 23
VII.3.6 - Contact by post or email 6 months after surgery 23
VII.3.7 - Contact 12 months after surgery (V4) 23
VII.4 - EXITING THE TRIAL AND EARLY WITHDRAWAL 24
VII.5 - PROCEDURES FOR LIMITING MISSING DATA 24
VII.6 - SPECIFIC CHARACTERISTICS OF THE STUDY IN RELATION TO THE PATIENT'S USUAL CARE 24
VII.7 - DESCRIPTION OF THE RULES FOR PARTIAL OR FULL TERMINATION OF THE STUDY 25

VIII - QUALITY CONTROL AND ASSURANCE 25

VIII.1 - MONITORING PROCEDURES 25
VIII.2 - TRANSCRIPTION OF INFORMATION INTO THE CASE REPORT FORM 26

IX - STATISTICAL DATA MANAGEMENT 27

IX.1 - RESPONSIBILITY FOR THE DATA ANALYSIS AND SOFTWARE USED 27
IX.2 - JUSTIFICATION OF THE REQUIRED NUMBER OF SUBJECTS 28

IX.3 - ANALYSIS STRATEGY FOR COLLECTED DATA 28
IX.4 - JUSTIFICATION OF STATISTICAL TESTS 28

X - SAFETY ASSESSMENT 30

X.1 - DEFINITIONS 30
X.2 - DESCRIPTION OF THE SAFETY ASSESSMENT PARAMETERS 31
X.3 - SERIOUS ADVERSE EVENT MANAGEMENT PROCEDURE 32
X.4 - STUDY-SPECIFIC COMMITTEES 34
X.4.1 - Steering committee 34
X.4.2 - Independent monitoring committee 34

XI - RIGHT TO ACCESS THE INFORMATION AND SOURCE DOCUMENTS 34

XII - LEGAL AND ETHICS ASPECTS 35

XII.1 - REQUEST FOR AUTHORISATION FROM THE ANSM 35
XII.2 - REQUEST FOR OPINION FROM THE ETHICS COMMITTEE 35
XII.3 - AMENDMENTS 35
XII.4 - CNIL DECLARATION 36
XII.5 - INFORMATION SHEET AND INFORMED CONSENT FORM 36
XII.6 - FINAL STUDY REPORT 36

XIII - DATA PROCESSING AND STORAGE OF STUDY-RELATED DOCUMENTS AND DATA 37

XIV - INSURANCE AND SCIENTIFIC COMMITMENT 37

XIV.1 - INSURANCE 37
XIV.2 - PRINCIPAL INVESTIGATOR'S COMMITMENT 38

XV - RULES REGARDING PUBLICATION 38

XVI - LIST OF ANNEXES 39

Annex 1 – Literature references 40
Annex 2 – Investigators and Associate Teams 44
Annex 3 – WOMAC Questionnaire Functional Scale 45
Annex 4 – MOS SF-12 Quality of life questionnaire 47
Annex 5 – Quality of life questionnaire: EQ-5D-3L 49
Annex 6 – RAPT (Risk Assessment and Prediction Tool) 51
Annex 12 52
Annex 13 53

SUMMARY

**Title**	Evaluation of an Educational Program Associated With Exercises (EDEX) Before Total Knee Arthroplasty
**Principal Investigator**	Prof. François Rannou (Cochin Hospital)
**Research sites**	- Cochin Hospital<br>- Lariboisière Hospital<br>- Clermont-Ferrand University Hospital
**Study duration**	60 months (45-month enrolment period)
**Patient follow-up**	Between 14 and 15 months, depending on the time between the enrolment visit and the intervention for the educational programme combined with an exercise programme
**Study aim**	<u>Primary objective</u>:<br>To assess the effectiveness of a standardised education and exercise programme proposed before a total knee replacement for knee osteoarthritis, in terms of:<br>- ability, at discharge from the surgical department at D4, plus or minus 1 day, to carry out lying-sitting and sitting-standing transfers, walk 30 metres and go up and down one flight of stairs;<br>- speed of functional recovery within the first 6 months (area under the curve of the function subscale of the WOMAC index).<br><br><u>Secondary objectives:</u><br>- to assess the effectiveness of the intervention in terms of pain, function (personalised WOMAC), quality of life, number of steps and treatment satisfaction at 6 and 12 months;<br>- to estimate and compare the cost of the strategies and, where appropriate, carry out a cost-effectiveness analysis.
**Methodology**	A multi-centre, randomised, controlled study
**Number of sites**	3 sites
**Number of patients**	**300 patients will be enrolled:**<br>150 patients in the experimental arm<br>150 patients in the control arm (usual information and advice given by the surgeon)
**Selection criteria**	<u>Inclusion criteria:</u><br>- Men or women aged from 50 to 85 years;<br>- Patients with knee osteoarthritis according to the ACR criteria for which a total knee arthroplasty has been scheduled by the surgeon;<br>- Medical examination carried out in advance, the results of which will be communicated to the patient;<br>- Patients having provided their written informed consent to take part in the study;<br>- Patients affiliated with a social security scheme (beneficiary or entitled party).<br><br><u>Exclusion criteria:</u><br>- Institutionalised patients;<br>- Patients having already undergone ipsilateral total knee arthroplasty;<br>- Patients with chronic inflammatory arthritis;<br>- Cognitive or behavioural disorders making the assessment impossible;<br>- Difficulties in understanding and communicating in French;<br>- TKA for an indication other than knee osteoarthritis.
**Early withdrawal criteria**	- Failure to meet the eligibility criteria<br>- The onset during the study of a condition listed in the exclusion criteria

**Study treatment**	4 group sessions consisting of a therapeutic education session and a physical exercise programme
**Study outline**	- Enrolment visit (V0);<br>- Intervention (education programme combined with an exercise programme);<br>- Preoperative visit;<br>- Surgery department discharge visit (medical record information);<br>- Monthly follow-up by post or email in terms of function (and personalised) subscale of the WOMAC index and pain in the past 48 hours, 6 weeks after surgery, 4 and 5 months after surgery, and 6 months after surgery with a self-questionnaire.<br>Postoperative self-questionnaires at 3 and 12 months, according to the patient's preference:<br>- Self-questionnaires given at a consultation;<br>- or self-questionnaires will be sent by post (prepaid envelope);<br>- or self-questionnaire information can be collected by a clinical study technician by phone.
**Primary and secondary endpoints**	Primary endpoints<br>- Ability at discharge: Assessed at D4, plus or minus 1 day. The endpoints to be assessed will be the ability to carry out lying-sitting and sitting-standing transfers, walk 30 metres and go up and down one flight of stairs (percentage of patients with a score of 3 out of 3 for the 4 items on the day of discharge);<br>- Speed of postoperative functional recovery at 6 months (area under the curve of the function subscale of the WOMAC index).<br><br>Secondary endpoints<br>- Pain assessment (numeric scale), function (personalised WOMAC index), quality of life (MOS SF-12, EQ-5D-3L), number of steps and treatment satisfaction;<br>- Cost comparison between the two strategies from the Health Insurance perspective and from the care system perspective, as a cost-effectiveness ratio.
**Statistical analysis**	An intention-to-treat analysis will be carried out with the data.<br>The statistical analysis will be carried out at the Prof. P. Ravaud Clinical Ethics Centre - Hôtel Dieu hospital, under the responsibility of Prof I. Boutron and G. Baron. SAS software will be used.

I - INTRODUCTION

I.1 - INFORMATION IN THE LITERATURE AND JUSTIFICATION OF THE RESEARCH

Knee osteoarthritis leads to deficiencies in muscle strength, mobility and balance, and a deterioration in cardiopulmonary function, which contribute to changes in patients' functional capacities, affecting gait in particular [Viton, 1999]. It is exacerbated by excess weight and a sedentary lifestyle, and specific education measures are able to reduce these two risk factors [Ravaud 2008]. It is the main cause for a total knee arthroplasty (TKA) being performed. In the United States, 400,000 knee arthroplasties were performed in 2005 and the expected increase in knee osteoarthritis surgery is in the order of 700% by 2030 [Kurtz 2007]. In Canada, from 2004-2005, the performance of TKAs has more than doubled (representing an increase of 124.8%) since 1994-1995 [Canadian Institute for Health Information, 2006]. Patients' functional status and pain level are frequently improved after undergoing TKA, and the pre-TKA physical and functional status (WOMAC) is predictive of postoperative recovery following arthroplasty [Fortin 1999].

The methods of postoperative management are changing. Developments in surgical techniques tend to reduce the length of stay at surgical departments and encourage the direct return home after an elective orthopaedic intervention. Recommendations by the French National Health Authority (HAS) [HAS 2006] and the transition to activity-based pricing (T2A) contribute to shortening the length of hospital admissions to short stays and changing the conditions for admission to a Physical Medicine and Rehabilitation (PMR) facility. Rehabilitation carried out during the preoperative period could enable patients to be better prepared for the intervention, have better functional results and become independent more quickly following the surgery, thereby reducing the length of stay and facilitating the methods of returning home (whether directly or after a stay at a PMR facility). The type of programme required to meet these objectives is still to be defined.

A literature review assessed the benefit of preoperative physiotherapy without multidisciplinary rehabilitation treatment before total knee arthroplasty (TKA) and total hip arthroplasty (THA) [Ackerman 2004]. This review included five randomised, controlled studies: three on the knee, one of which for unicompartmental knee arthroplasty [Weidenheim 1993], and two studies on the hip, from the same patient cohort [Gilbey 2003; Wang 2002]. It concluded the preoperative physiotherapy to be ineffective on the deficiencies and functional incapacity for TKA. However, the studies analysed have significant methodological limitations and most involve a small number of patients, which limits any conclusions being drawn from this analysis.

Educational preoperative interventions have also been assessed. A Cochrane Library review [McDonald 2004] assessed the impact of preoperative education before hip and knee

arthroplasty. This review included nine studies, four of which had length of postoperative stay and functional incapacity as endpoints. Only one study [Crowe 2003] has shown a positive effect in terms of reducing the length of stay without having an impact on functional capacity. This study involved an occupational therapy intervention and proposed personalised management adapted to each patient. This study suggests that the combination of education and exercises could be more effective than a single isolated education measure.

A larger number of studies conducted on osteoarthritis have shown rehabilitation to be beneficial in terms of functional capacities [Fransen 2003]. These studies have allowed for recommendations to be drafted for the practice, recommending exercises for treating knee osteoarthritis. However, the main limitation is that they focus only on medical treatment of lower-limb osteoarthritis before the arthroplasty stage [Hochberg, 1995: 1535, Hochberg 1995: 1541, Pendleton 2000, Roddy 2005, Zhang 2005, Zhang 2006].

The Philadelphia Panel [Philadelphia Panel 2001] and the French Society of Physical Medicine and Rehabilitation, in association with the French Society of Rheumatology and the French Society of Orthopaedic Surgery and Traumatology [Rannou 2007] have taken a pragmatic, multidisciplinary approach combining the systematic analysis of the literature and taking into account practices by differentiating between the pre- and postoperative phase of knee arthroplasty [Coudeyre 2007, Fedmer 2001, Genêt 2007, Thoumie 2007, Barrois 2007, Coudeyre 2007, Lefevre-Colau 2007, Paysant 2007, Froehlig 2008]. The review carried out by the Philadelphia Panel in 2001 [Philadelphia Panel 2001] focussed only on rehabilitation before total knee arthroplasty. It included only one single study [D'Lima 1996] and was unable to reach a conclusion regarding the benefit of the reinforcement exercises. A systematic literature review combined with a practice analysis regarding the benefit of rehabilitation before TKA or THA by a French multidisciplinary panel of experts [Coudeyre 2007] included 10 articles, 3 of which were on TKA and 1 on TKA and THA. The authors concluded that carrying out preoperative rehabilitation before TKA is highly likely to be beneficial, particularly for reducing the length of stay at the surgical department and improving the methods for returning home, and recommended that this rehabilitation should include, as a minimum, exercises combined with educational management. This management would be particularly desirable for very fragile patients with altered functional capacities, comorbidities and/or social issues. The authors highlight that therapeutic trials of good methodological quality which differentiate between TKA and THA are required to support these conclusions.

I.2 - EXPECTED RESULTS AND PROSPECTS

The study will be conducted in patients between the ages of 50 and 85 years with knee osteoarthritis for which total knee arthroplasty has been scheduled by the surgeon. Two patient groups will be studied: one of the two groups will undergo four group sessions including therapeutic education and physical exercises, a knee arthroplasty guide and an exercise CD at least 2 months before the intervention; the other group (control group) will receive the usual information and advice given by the surgeon and according to each centre, and a knee arthroplasty guide.

If this programme is confirmed to be effective, it should lead to a reduction in the length of hospital stay and rate of transfers to rehabilitation care facilities, achieving quicker and more complete functional recovery, improving the patient's quality of life and satisfaction, and reducing treatment costs.

I.3 - STUDY FEASIBILITY

The feasibility of the study is attested by:

- The experience of the clinical teams in the pre- and postoperative management and treatment (particularly treatments including education and physiotherapy) of TKA, with the institutional recognition of the orthopaedic centres ensuring sufficient recruitment;
- Standardisation of the intervention;
- The existence of a cross-functional, complementary steering committee: methodological, logistical and clinical, with an adviser per department, including the principal investigator.

I.4 - EXPECTED BENEFITS AND RISKS

Carrying out a multidisciplinary programme combining education and exercises should lead to quicker and more complete functional recovery, reduce the length of hospital stay and the rates of transfers to rehabilitation care facilities, improving the patient's quality of life and satisfaction, and reducing treatment costs.

The risks are minimal, and are related to the adverse events which may occur during the study.

We have no known or reported factors regarding the risks related to the education programme.

The main risks related to the exercise programme are the increase in exercise-related pain and exercise-related injuries to muscles (cramps, elongation), ligaments (sprain) and joints (effusion). If any events occur, the patients will be assessed by the doctor in charge of the study for their treatment. These are minor, non-progressive injuries which are quick to heal, as the exercise intensity will be adapted to the patient's pathology and age, under the supervision of a physiotherapist.

II - STUDY OBJECTIVE

II.1 - PRIMARY OBJECTIVE

The primary objective of this study is to assess the effectiveness of the programme on:

- ability, at discharge from the surgical department at D4, plus or minus 1 day, to carry out lying-sitting and sitting-standing transfers, walk 30 metres and go up and down one flight of stairs;
- speed of postoperative functional recovery within the first 6 months (area under the curve of the function subscale of the WOMAC index).

II.2 - SECONDARY OBJECTIVES

The secondary objectives in these patients with knee osteoarthritis for whom a TKA is scheduled are as follows:

1) Clinical assessment at 6 months and 12 months:

  a. function (WOMAC);
  b. pain (numeric scale 0-100);
  c. quality of life (SF-12 and EQ-5D-3L);
  d. number of steps (pedometer);
  e. treatment satisfaction.

2) Health economics evaluation for the intervention. The costs of the two strategies will be compared at 6 and 12 months by taking into account the perspectives of the payer (Health Insurance) and the healthcare system. In addition, patients' quality of life will be estimated at the enrolment visit, and 6 months and 12 months after the procedure, using the EuroQoL questionnaire (EQ-5D-3L) [Brooks 1996, Fransen 1999] (Annex 5). In the event that the most expensive strategy is the one that provides the best medical outcome and the best quality of life, we will carry out a cost-effectiveness analysis.

Implementing and evaluating complex intervention strategies such as combining educational and supervised exercise programmes is complicated and raises a number of specific methodological issues. Recently published recommendations [Craig 2008] highlight the need to take into account multiple endpoints. Indeed, complex interventions have multiple objectives (improving function, patient information and changing patient behaviour in terms of physical activity and weight-loss).

III - METHODOLOGY

III.1 - STUDY TYPE

This is a randomised, controlled, multi-centre, prospective study.

III.2 - STUDY DURATION

The planned enrolment period is 45 months. The study is therefore expected to last 60 months.

The patients taking part in the study will be monitored for between 14 and 15 months depending on the time elapsed between the enrolment visit and the intervention for the multidisciplinary programme.

The patients will not be able to take part in a biomedical research study with therapeutic assessment likely to change the primary and secondary endpoints of the EDEX study for a duration of 15 months.

Following the study, there is no exclusion period during which participation in another study is prohibited.

III.3 - EXPERIMENTAL DESIGN

We are going to implement a randomised, controlled, multi-centre trial comparing:

1) usual treatment provided by the surgeon;
2) usual treatment provided by the surgeon combined with standardised and personalised multidisciplinary treatment.

The randomised, controlled trial is considered the gold standard for therapeutic assessment.

The study plan, its implementation, analysis and final write up of the results will be carried out in accordance with the CONSORT statement recommendations [Altman *et al.* 2001] and their extensions for non-pharmacological trials [Boutron, Ravaud *et al.* 2008], and the recommendations of the Medical Research Council for developing and evaluating complex interventions [Craig *et al.* 2008].

The patients randomised into the control group will not have any specific treatment apart from the standard treatment proposed by the surgeon and the messages from a knee arthroplasty guide. This choice allows patients to be in the situation which most accurately represents the current clinical practice and takes into account the lack of a standard treatment having proven to be effective which will be used according to consensus for all the sites.

This choice leads to some methodological issues, as blinding of patients, surgeons and the medical and paramedical team responsible for patient management will not be possible. As a result, there will be a risk of bias in performance and assessment. To take into account these risks, we will:

a) Systematically collect the co-interventions prescribed in each group (analgesic treatments, physiotherapy prescription, number of consultations with the surgeon) to assess the risk of contamination and performance bias;

b) Organise an independent assessment by a clinical study technician.

The patients will be informed that the aim of this study will be to compare two treatments for patients in the preoperative period, which are different and possibly effective. To this end, we will explain that they can have either routine care provided by their surgeons, or multidisciplinary care consisting of 4 exercise and therapeutic education sessions while awaiting the surgical intervention, in addition to the routine care.

III.4 - PATIENT ENROLMENT AND RANDOMISATION

Randomisation characteristics

Randomisation will be stratified by site. The randomisation list will be prepared with variable block sizes. The list and block size will not be communicated to the investigators. The secrecy of the assignment will be maintained using an electronic randomisation system.

Enrolment and randomisation procedure

Taking into account the lack of availability of clinicians, and surgeons in particular, patient enrolment in clinical trials is difficult. To compensate for this difficulty, patient screening will be carried out by a clinical study technician.

The clinical study technician will systematically identify all patients who have been scheduled to undergo knee arthroplasty. As agreed with the surgeon in charge of the patient, the clinical study technician will contact the potentially eligible patient and will propose that he or she participate in the EDEX study. If the patient is interested in this study, a consultation will be scheduled, which must take place on the same day as the pre-anaesthesia consultation. During this consultation, the rehabilitation physician or the rheumatologist, assisted by the clinical study technician, will confirm the eligibility criteria, will give the patient the relevant information and, after obtaining the patient’s consent, the patient will be randomised. Patients randomised into the intervention group will be contacted by the clinical study technician to arrange for the intervention to be carried out.

IV - STUDY POPULATION

IV.1 - INCLUSION CRITERIA

- Men or women aged from 50 to 85 years;
- Patients with knee osteoarthritis according to the ACR criteria for which total knee arthroplasty has been scheduled by the surgeon;
- Medical examination carried out in advance, the results of which will be communicated to the patient;
- Patients having provided their written informed consent to take part in the study;
- Patients affiliated with a social security scheme (beneficiary or entitled party).

IV.2 - EXCLUSION CRITERIA

- Institutionalised patients;
- Patients having already undergone ipsilateral total knee arthroplasty;
- Patients with chronic inflammatory arthritis;
- Cognitive or behavioural disorders making the assessment impossible;
- Difficulties in understanding and communicating in French;
- TKA for an indication other than knee osteoarthritis.

IV.3 - REQUIRED NUMBER OF SUBJECTS

A total of 150 patients are planned to be enrolled in the experimental group (multidisciplinary programme) and 150 patients in the control group, i.e. 300 patients in total.

IV.4 - DIAGNOSIS STANDARDISATION

Knee osteoarthritis according to the ACR criteria for which total knee arthroplasty has been scheduled by the surgeon.

IV.5 - RECRUITMENT METHOD

Patients will be recruited from the Orthopaedics, Rheumatology and PMR departments at three centres specialising in the surgical treatment of knee osteoarthritis (Cochin Hospital, Lariboisière Hospital and Clermont-Ferrand University Hospital). Patient recruitment will be done through the usual correspondents, via orthopaedics consultations at the three hospitals. The plan is for the orthopaedists to send potential patients' information to the 3 recruiting sites.
A written information sheet will also be distributed by the sites to free and hospital rheumatologists in the Ile-de-France and Auvergne regions.

Recruitment potential within the sites

280 patients were admitted to the Orthopaedics Department at Cochin Hospital over the past 12 months to undergo a TKA (70% knee osteoarthritis). Over 200 knee arthroplasties are performed each year at the Clermont-Ferrand (90% knee osteoarthritis) and Lariboisière (70% knee osteoarthritis) centres. If we give each of these sites a minimum enrolment hypothesis of 30% of patients in the study, the annual enrolment possibilities may be estimated to be around 160 patients per year.

V - STUDY TREATMENT

V.1 - TREATMENT OUTLINE

As part of this randomised, parallel-group study, 300 patients will be divided into 2 arms (150 patients per arm):

<u>EXPERIMENTAL ARM:</u>

The sessions take place in groups (4 to 6 patients) and include education, performance and teaching of a personalised exercise programme. These sessions begin at least 2 months before the knee arthroplasty is performed.

Supervised session schedule: 2 supervised sessions lasting 1 and a half hours per week over 2 weeks.

Content: Programme preparation and planning are carried out according to the Medical Research Council Guidance recommendations for developing and evaluating complex interventions [Craig *et al.* 2008]. The intervention is developed and defined by a group of experts including orthopaedists, physical medicine and rehabilitation physicians, rheumatologists, physiotherapists, dieticians, psychologists and education science specialists. The group defines the main components of the intervention, its objectives, potential barriers, how to overcome them and how to optimise patient adherence to the messages received.

Therapeutic education sessions (Annex 7): in order to optimise the effect of the messages received, we tested the hypothesis already suggested in other situations (Munjanja SP 1996, Ravaud 2008) that an intervention proposing fewer, but more targeted, messages is more effective than a standardised education programme. To this end, 4 education classes are proposed with very different, simple messages.

In order to improve patient adherence, the first session is dedicated to physical activity before undergoing a TKA and walking after this surgery. The other 3 sessions are based on a single component (orientation at discharge, dietary advice and environment planning). This enables the message to be simplified, better understood by patients and easier to remember. In order to standardise the intervention, training sessions are organised for each of the educational sessions, and group support is provided.

It is made up of four 30-minute sessions before the exercise sessions:

<u>1st session: physical exercise before the arthroplasty.</u>

The role of physical exercise on health and participation, which exercises to do while awaiting surgery, when and how to do them when you return home.

We will confirm that the pedometer is used properly (Annex 10). For active patients, the aim will be to maintain the level of activity. For sedentary patients, the aim will be to gradually increase the number of steps measured using a pedometer by 20% before the surgery. This session is led by a physiotherapist or a sports instructor.

At the end of this session, the physiotherapist will give the patients the self-exercise programme to be done at home.

2nd session:

1st part: Presentation of the criteria for discharge and transfer to the rehabilitation care facilities centre (RAPT table, Annex 6). During this session, we will discuss the patient's opinion regarding their postoperative orientation and how confident they are about returning home.

This session is led by a social worker.

2nd part: Diet

Individual analysis of dietary habits, group reflection on issues such as how to manage weight in the future, how to prevent excessive weight gain after the intervention, possible sources of help, specialist consultations, the psychological consequences of weight gain and management, and the obstacles preventing weight loss. This session will be tailored according to how overweight the patient is, which will be determined according to body mass index (BMI) and their willingness to lose weight. Patients willing to lose weight will be given advice regarding the circuits and the possible treatment methods for achieving this target. Overweight patients who are not very willing to lose this weight will be given advice on how to maintain their current weight.

This session is led by a dietician.

3rd session: preoperative anxiety management.

How to recognise surgery-related anxiety, how to manage it and when to see a doctor. This session is supervised by a psychologist.

4th session: temporary household planning.

When and how to plan your environment How to optimise transfers Presentations of technical aids and advice on how to use them. Individual adaptations of environment and technical aids to facilitate the return home will be discussed based on the description of the patient's environment and lifestyle.

This session is organised by an occupational therapist.

Support materials for these sessions will be in the form of slideshows, pre-TKA exercise CDs and crutches; group discussion and questions. Patients will leave with a knee arthroplasty guide and a self-exercise programme.

Exercise sessions (Annex 8): 4 one-hour sessions after the therapeutic education sessions.

CONTROL ARM:

Usual information and advice given by the orthopaedics department at each centre and knee arthroplasty guide. Patients will also receive a pedometer to measure the number of steps and will learn how to use this on the day of the enrolment visit (Annex 10).

V.2 - ASSOCIATED TREATMENTS

All treatment administered to the patient during the trial must be reported in the case report form.

V.2.1 - AUTHORISED TREATMENTS

The prescription of medicinal products, particularly analgesics and NSAIDs, will be left to the investigator's discretion. Any treatment taken or likely to be taken by the patient during the study must be reported in the self-assessment diary, which will be given to the patient at the enrolment visit.

V.2.2 - PROHIBITED TREATMENTS

There will be no prohibited treatments in the study.

V.3 - PROVISIONS PUT IN PLACE TO COMPENSATE FOR THE LACK OF BLINDING

As the type of treatment proposed does not allow for blinding of the patient or the therapist, various measures will be taken to compensate for the lack of blinding:

- Evaluator blinded to treatments;
- Systematically collect the co-interventions prescribed in each group (analgesic treatments, physiotherapy prescription, number of consultations with the surgeon) to assess the risk of contamination and performance bias.

V.4 - PATIENT CARD

In application of the Good Manufacturing Practice regulations dated 26 May 2006, a patient card will be issued.

VI - ENDPOINTS

VI.1 - PRIMARY ENDPOINTS

The first primary endpoint will be the ability at discharge from the surgical department at D4, plus or minus 1 day. We will assess the four tests described by Zavadak KH *et al*, from D1 to D5 post-surgery:

- transferring from the lying position to the seated position;
- transferring from the seated position to the standing position;
- walking 30 metres;
- going up and down one flight of stairs.

These tests will be performed at four levels of independence:

- level 0: the test cannot be performed

- level 1: the test is performed with physical assistance from a third party
- level 2: the test is performed with verbal assistance from a third party
- level 3: the test is performed without any assistance from a third party

These four tests for measuring the level of dependence in the early postoperative period are the most widely used [Dowsey 1999, Ganz 2003, Kwoh 1997, Munin 1995, Munin 1998, Walker 2001, Zavadak 1995]. The use of sticks or a walker is allowed at all levels. Only intervention from a physical person, whether verbal or mechanical, is considered to be assistance. A patient will be considered to be independent if he or she performs the four tests at level 3. The level of dependency will be assessed every day from D1 to D5 during the patient's hospital stay, by an independent clinical study technician, who is specifically trained and is unaware of the assigned intervention (blinded). The percentage of patients who are independent on the day of discharge from the orthopaedics department will be the primary endpoint.

The second primary endpoint will be the assessment of functional recovery 6 months after surgery using the area under the curve of the function subscale of the WOMAC index.

VI.2 - SECONDARY ENDPOINTS

Efficacy criteria

At 6 months and 12 months:

- The average change from baseline in the average knee pain intensity in the past 48 hours, assessed using a self-administered 11-point numeric scale (0 = no pain and 100 = maximum pain)
- The average change from baseline in the average function assessed using the area under the curve of the function subscale of the WOMAC index (0 = best possible function and 100 = worst possible function)
- The average change from baseline in the average quality of life assessed using the physical and psychological components of the MOS SF-12 score (0 = worst possible quality of life and 100 = best possible quality of life) and using the EQ-5D-3L score (1111 = best possible quality of life and 33333 = worst possible quality of life)
- The average change from baseline in the average self-reported number of steps in the past week, measured using a pedometer.
- The average treatment satisfaction assessed using a self-administered 11-point numeric scale (0 = zero satisfaction and 100 = maximum satisfaction).
- The cost-effectiveness assessed using the cost-effectiveness ratio.

-

VII - PATIENT STUDY PLAN

GENERAL OUTLINE OF THE STUDY

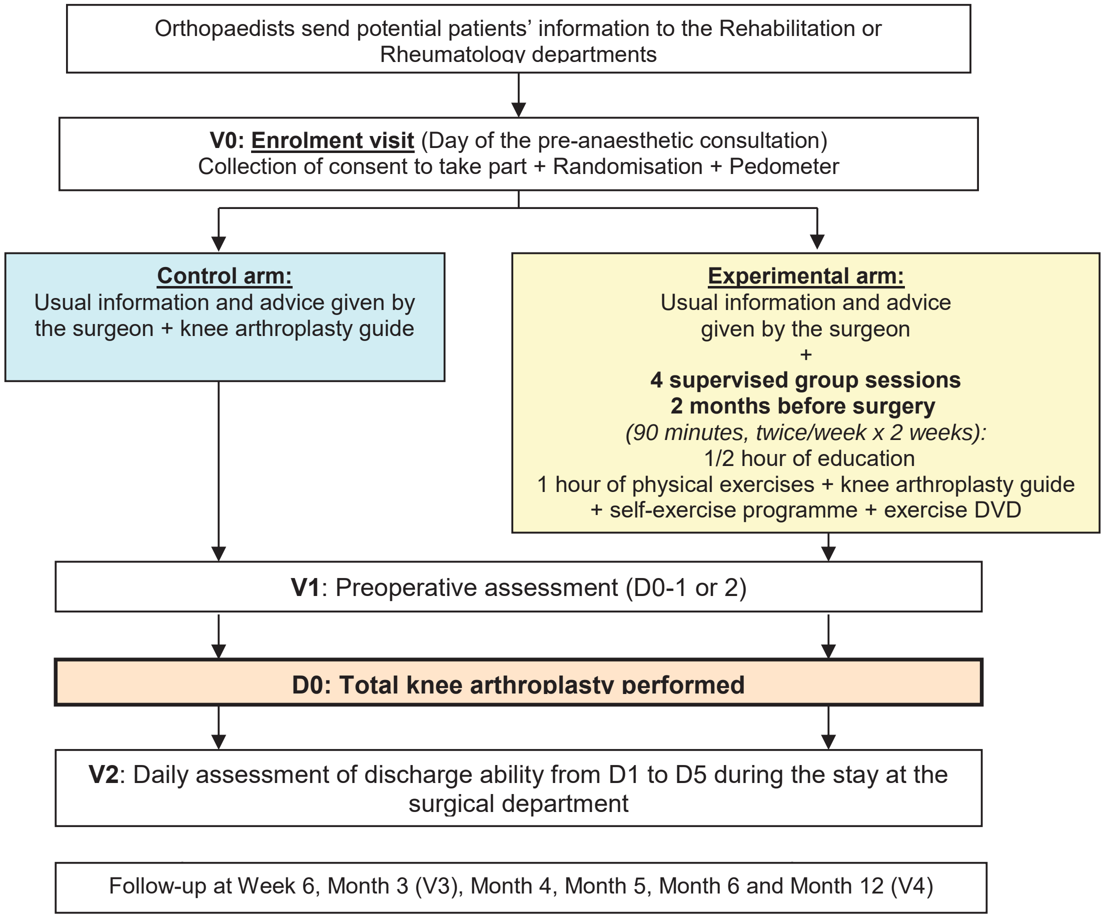

	Enrolment visit	Preoperative visit (V1)	Surgical discharge visit (V2)	Contact at 6 weeks	Contact at M3 (V3) (d)	Contacts at M4, M5	Contact at M6	Contact at M12 (V4) (d)
Information and consent	+							
Randomisation	+							
Weight, height, BMI	+							
WOMAC	+	+		+	+	+	+	+
Numeric pain scale	+	+		+	+	+	+	+
Discharge ability			D1 to D5					
Actual length of stay			+					
Rate of transfer to rehabilitation care facilities			+					
Length of stay at rehabilitation care facilities				+	+		+[(a)]	
MOS SF 12	+	+			+		+	+
EQ-5D-3L	+						+	+
RAPT	+							
Adaptation to environment		+			+			
Number of steps	+[(b)]	+[(c)]			+[(c)]		+[(c)]	+[(c)]
Treatment satisfaction							+	+
Co-interventions	+	+			+		+	+
Adverse Events		+	+		+		+	+

(a) If the length of stay is longer than 6 weeks

(b) To be performed the week after the enrolment visit (7 days)

(c) To be performed the week before the enrolment visit (7 days)
(d) According to the patient's preference, during a consultation, sending of self-questionnaires by post (prepaid envelope) or self-questionnaire information collected by a clinical study technician over the phone

VII.1 - VISIT V0 - ENROLMENT VISIT

The patients selected within the surgical department at Clermont-Ferrand University Hospital and Lariboisière and Cochin hospitals will be contacted by the clinical study technician and, if they are potentially interested in the study, they will be referred to the PMR departments at Cochin and Clermont-Ferrand and the Lariboisière rheumatology department for their enrolment visit and their study follow-up.

At the enrolment visit, the rehabilitation physician or the rheumatologist, with the help of the clinical study technician, will systematically confirm the inclusion and exclusion criteria. The study methods will be clearly explained to the patient. The patient will be given an information sheet and informed consent form. If the patient agrees to take part in the study, he or she will sign the informed consent form.

The following information will be collected in the electronic case report form:

- Demographic parameters (age, sex);
- Socioprofessional parameters (level of studies, profession and professional status (working, on sick leave, on disability, retired));
- Medical examination (weight, height and BMI);
- Inclusion and exclusion criteria;
- Information and consent;
- Demonstration of how to use the pedometer to measure the number of steps taken in the week after the enrolment visit;
- Co-interventions (particularly analgesics and/or NSAIDs, physiotherapy).

Patients will fill in a self-assessment card during the visit, including assessment of the following:

- knee pain over the past 48 hours, assessed using a visual numeric scale from 0 to 100;
- preference regarding hospital discharge destination and RAPT scale (Annex 6);
- function according to the WOMAC scale and the personalised section;
- quality of life (MOS SF-12, Annex 4 and EQ-5D-3L, Annex 5).

The time elapsed between the enrolment visit and the start of the multidisciplinary programme will be no more than one month.

Randomisation: patients will be randomised using CleanWeb software.

After randomisation, the patients in the two arms will receive a copy of the Knee Arthroplasty Guide (Annex 11).

The patients randomised into the “standard-treatment” control arm will receive standard medical care.

VII.2 - INTERVENTION PROTOCOL

Patients will be seen at the day hospital (third-category rehabilitation care facilities) on four occasions over two weeks, at least two months before the intervention.

Intervention: The multidisciplinary programmes will be carried out at the Rehabilitation Department at Cochin Hospital and Clermont-Ferrand Hospital and at the Lariboisière Rheumatology Department. The programme is prepared according to the Medical Research Council Guidance recommendations for developing and evaluating complex interventions (Craig *et al.* 2008). This requires expert consensus, preparation of an information booklet for patients, and training for trainers. All investigators have vast experience in treating patients with TKA. The intervention is never carried out by surgeons, which prevents the risk of contamination.

Patients in the intervention group will receive four 1.5-hour supervised group sessions (4 to 6 patients) consisting of 30 minutes of education and 1 hour of physical exercise at least two months before surgery with two sessions per week for two consecutive weeks.

Transfers to the four multidisciplinary treatment sessions will be covered by the study.

Patients in the control group will receive the usual information and advice given by the orthopaedics department at each centre in line with their usual practice and a knee arthroplasty guide (Annex 10), as well as a pedometer to measure the number of steps (Annex 9).

The multidisciplinary programme is standardised and personalised (Annex 7). For the educational part, the four sessions will be divided up as follows:

- The first session led by a physiotherapist deals with the role of physical activity and exercises before undergoing the arthroplasty and walking after the procedure. It will be checked that the pedometer is used properly. For active patients, the aim will be to maintain the level of activity. For sedentary patients, the aim will be to gradually increase the number of steps measured using the pedometer by 20% before the surgery.
- The second session will be led by a social worker and a dietician. The social worker deals with the topic of “information regarding postoperative orientation” according to the patient’s characteristics (RAPT table, Annex 6). During this session, the patient will discuss their opinion regarding their postoperative orientation and how confident they are about returning home. The second session deals with the role of excess weight and how to control weight. This session will be tailored to the patient’s level of excess weight, which will be determined according to body mass index (BMI) and willingness to lose weight. Patients

willing to lose weight will be given advice regarding circuit training and the possible treatments for achieving this target. Overweight patients who are not very willing to lose this weight will be given advice on how to maintain their current weight.

- The third session led by a psychologist deals with issues such as: how to recognise the symptoms of surgery-related anxiety, how to manage anxiety and when to seek professional help.

The fourth session will be led by an occupational therapist. The rules on adapting the environment, identifying accessibility barriers and the main possible technical support tools will be presented to patients. Individual adaptations of environment and technical aids to facilitate the return home will be discussed based on the description of the patient's environment and lifestyle.

For the exercise part (Annex 8), the sessions include muscle-reinforcement exercises for the lower limbs, endurance exercises (aerobic activity), proprioceptive and balance exercises, comprehensive walking exercises and teaching of a personalised exercise programme (Annex 9) to be carried out no more than once per day for 20 to 30 minutes separately from the aerobic exercise (brisk walking at 60% of the theoretical maximum heart rate) to be carried out no more than 3 times per week for 1 hour. The programme will be adjusted according to the patients' preferences and their motivation to carry out the exercise. The maximum programme (30 minutes of exercises per day, brisk walking 3 times per week) will be proposed to the most motivated and most active patients. The minimum recommended programme will consist of three exercise sessions and one period of brisk walking per week. The amount of exercises carried out (observance) will be noted down by the patient in a weekly diary while awaiting the arthroplasty.

Standardisation among the three sites will be double checked before the start of the study.

VII.3 - FOLLOW-UP VISITS AFTER THE INTERVENTION

VII.3.1 - PREOPERATIVE VISIT (V1)

At the preoperative visit, which will take place one or two days before the surgery, the following information will be collected in the electronic case report form:

- Professional status;
- Number of steps measured by the patient in the week (7 days) before this visit;
- Co-interventions since the enrolment visit;
- Adverse events.

Patients will fill in a self-assessment card including assessment of the following:

- knee pain over the past 48 hours (numeric scale);
- function according to the WOMAC scale and the personalised section;
- quality of life (MOS SF-12);
- adaptation of environment.

VII.3.2 - VISITS DURING THE STAY AT THE SURGICAL DEPARTMENT (V2)

Discharge ability, one of the main endpoints, will be assessed daily by an independent clinical study technician from postoperative D1 until D5 (see section VI-1 of the protocol).

The evaluating physician or clinical study technician will take specific clinical information from the medical file the day before the patient is discharged from the Orthopaedics Department (actual length of stay, rate of transfer to rehabilitation care facilities and adverse events).

After discharge from the Surgical Department, patients will be monitored at consultations, consisting of one visit at 3 months and one at 12 months. These two visits will take place as part of the usual follow-up consultations with the surgeon. Follow-up by post or email, according to the patient's preference, will take place after 6 weeks, 4, 5 and 6 months.

VII.3.3 - CONTACT BY POST OR EMAIL 6 WEEKS AFTER SURGERY

Patients will fill in a self-assessment card including assessment of the following:

- knee pain over the past 48 hours (numeric scale);
- function according to the WOMAC scale and the personalised section;
- a question on the length of stay at rehabilitation care facilities.

VII.3.4 - CONTACT 3 MONTHS AFTER SURGERY (V2)

<u>Postoperative self-questionnaires at 3 months, according to the patient's preference:</u>

- Self-questionnaires given to the investigator at a consultation;
- or self-questionnaires will be sent by post (prepaid envelope);

or self-questionnaire information can be collected by a clinical study technician by phone.

The information below will be collected:

- Professional status;
- Number of steps measured by the patient in the week before this visit;
- Length of stay at rehabilitation care facilities;
- Adverse events.

Patients will fill in a self-assessment card including assessment of the following:

- knee pain over the past 48 hours (numeric scale);
- function according to the WOMAC scale and the personalised section;
- quality of life (MOS SF-12);

- adaptation of environment;
- co-interventions since the preoperative visit.

VII.3.5 - Contact by post or email 4 and 5 months after surgery

During the monthly contacts, the WOMAC functional index and personalised section, as well as pain (numeric scale) over the past 48 hours, will be collected from the patient.

VII.3.6 - Contact by post or email 6 months after surgery

Patients will fill in a self-assessment card including assessment of the following:

- knee pain over the past 48 hours (numeric scale);
- function according to the WOMAC scale and the personalised section;
- number of steps measured by the patient in the week before this contact;
- quality of life (MOS SF-12 and EQ-5D-3L);
- length of stay at rehabilitation care facilities;
- treatment satisfaction;
- co-interventions since the previous visit;
- adverse events.

VII.3.7 - Contact 12 months after surgery (V4)

Postoperative self-questionnaires at 12 months, according to the patient's preference:

- Self-questionnaires given to the investigator at a consultation;
- or self-questionnaires will be sent by post (prepaid envelope);
- or self-questionnaire information can be collected by a clinical study technician by phone.

The information below will be collected:

- Professional status;
- Number of steps measured by the patient in the week before this visit;
- Adverse events.

Patients will fill in a self-assessment card including assessment of the following:

- knee pain over the past 48 hours (numeric scale);
- function according to the WOMAC scale and the personalised section;
- quality of life (MOS SF-12 and EQ-5D-3L);
- treatment satisfaction;
- co-interventions since the previous visit.

VII.4 - EXITING THE TRIAL AND EARLY WITHDRAWAL

Patients may leave the study at any time and for any reason, if they decide to do so. This will have no consequences on the quality of any subsequent care that the patient will receive. Patients may also be withdrawn from the trial at the investigator's discretion. All cases of patients withdrawing from the study must be documented.

In the event of early withdrawal, the investigator must fill in the case report form up to the date of early withdrawal. The reason for the early withdrawal must be specified. Patients withdrawing from the trial cannot be enrolled again in the study. The patient's treatment will continue in the context of routine care.

VII.5 - PROCEDURES FOR LIMITING MISSING DATA

In order to limit missing data, all patients not attending the 3-month and 12-month follow-up consultations (or those who cannot be reached) will be contacted by telephone or by email. The main aim of these reminders is to reschedule a follow-up consultation. In the event of the patient categorically refusing to attend these consultations, as much information as possible will have to be collected by telephone.

The same approach will be taken for patients who cannot be contacted by post or by email at 6 weeks, 4, 5 and 6 months.

VII.6 - SPECIFIC CHARACTERISTICS OF THE STUDY IN RELATION TO THE PATIENT'S USUAL CARE

The specific characteristics of the study are listed in the table below in relation to what is done as part of the patient's care:

Description of the procedures	CARE	STUDY
Advice given by the surgeon at the orthopaedic consultation	X	
Total knee arthroplasty guide	X	
4 exercise sessions		X
4 therapeutic education sessions		X
Preoperative assessment		X
Daily assessment of discharge ability from D1 to D5		X
Self-exercise programme and DVD		X
Contact 3 and 12 months after surgery	X	
Follow-up questionnaires at 6 weeks, 4, 5 and 6 months (by post, email or telephone)		X

VII.7 - DESCRIPTION OF THE RULES FOR PARTIAL OR FULL TERMINATION OF THE STUDY

An extraordinary meeting can be convened at the sponsor's request, in the event that any serious adverse events or results which would affect the study occur. The following will also be considered to be reasons to terminate the study:

- insufficient recruitment;
    a. if, one year after the official start date of the study, no enrolments have been made, the DRCI will make the decision to terminate the study early.
    b. if, after 30% of the time planned for enrolling subjects into the study, set from the date of the first enrolment, fewer than 15% of the subjects have been enrolled, a cause analysis must be carried out and corrective actions must be taken (re-motivation of sites, inclusion of additional sites, change in the criteria regarding the population to be enrolled, etc.).
    c. finally, if, after 50% of the provisional time for enrolments as defined above, fewer than 30% of the patients have been enrolled, the DRCI, in agreement with the Steering Committee, can make a decision regarding the definitive termination of enrolments, or even the early termination of the study, depending on the situation.
- the occurrence of excessive SAEs

VIII - QUALITY CONTROL AND ASSURANCE

The study will be conducted in accordance with the sponsor's standard operating procedures.
The performance of the study at the research sites and the treatment of subjects will be done in accordance with the Declaration of Helsinki and the Good Clinical Practice guidelines in force.

VIII.1 - MONITORING PROCEDURES

The study is classified as risk level A.
The CRAs representing the sponsor will carry out visits to the research sites according to the follow-up schedule for patients in the protocol, the enrolments at the various research sites and the level of risk assigned to the study.
- Start-up visit at each site: before enrolment, for implementation of the protocol and familiarisation with the various parties involved in the biomedical research study.
- At the next visits, the case report forms will be reviewed as the study progresses by the CRAs. The principal investigator at each site, as well as the other investigators who enrol or undertake follow-up with individuals participating in the study, agree to receive visits from the CRAs at regular intervals. In accordance with the Good Clinical Practice guidelines, the following items will be reviewed at the site visits:

- Compliance with the protocol and procedures set out for the study;

- Verification of the patient informed consent forms;
- Examination of the source documents and comparison with the data reported in the case report form in terms of accuracy, missing data and consistency of the data according to the regulations set out by the procedures of the DRCI.

- Closure visit: collection of case report forms, biomedical research documents, archiving.

VIII.2 - TRANSCRIPTION OF INFORMATION INTO THE CASE REPORT FORM

The study data will be collected using the CleanWeb electronic case report form, within the framework of a public contract between the AP-HP and TELEMEDICINE TECHNOLOGIES S.A., notified on 17/11/2003 (reference no. 033845) and renewed on 26/11/2006 (reference no. 063844). The data will be centralised in a server located at the Operational Services Department (DSO) of AP-HP, 67 boulevard Bessières – 75017 PARIS.

An initial version of the eCRF may be put online and tested after sending the specific study specifications by fax to the company TELEMEDICINE. Once the Coordinator, the Project Leader, the Clinical Research Unit, the Data Manager and the Statistician have agreed on the final version of the eCRF, and following the release of the credits, and submission of the purchase order to the company TELEMEDICINE by the DRCI, the eCRF will come into operation.

In accordance with the Good Clinical Practice guidelines, the case report form in which the study data are transcribed must correspond to at least the following standard presentation:

- At the start of the form, the following are normally included: the title of the study, the patient's study reference, possibly the contact information of the individual taking part in the study (first initials of their surname and first name), randomisation number (where applicable), and inclusion and exclusion criteria in the form of a check-list, which allows subject selection to be validated with respect to the study population. At the end of the study, when the study database has been "frozen", the eCRFs of each patient will be printed and signed by the investigator. The references of the study and of the person taking part in the study will then appear in the form of a slip on each page to allow data to be identified in all cases.
- The visit and/or sampling dates of the data transcribed must be reported in this eCRF as well as the point in the study to which they correspond.
- Results must contain the units of measurement, and even the laboratory standards in the event that these vary with the technique used.
- The following items must be included at the end of the eCRF:
    - Concomitant treatments;

- Non-serious adverse events (AEs);
- End of study/Early termination;
- Outside planning, an SAE module.

All the information required by the protocol must be provided in the case report form and an explanation must be provided by the investigator for any missing information.

Information must be transferred to the case report forms as soon as it becomes available, whether clinical or paraclinical information.

Incorrect information detected in the case report forms will be replaced in the form by a registered investigator, who will log in to the software with his or her access information (username and password). These codes are strictly personal and confidential, and under no circumstances may be passed on to third parties. They help ensure data confidentiality and authenticate the interventions. Access information is associated with an electronic signature system which validates the data entered by the investigator. Each signature is stamped with the date and time and recorded in the study audit trail. Signed information cannot be changed. However, the investigator may delete his or her signature if he or she wishes to correct any information. Deleting a signature is also subject to stamping with the date and time.

Subject anonymity will be guaranteed by mentioning no more than the number in the study, the initials of the surname and first name of the individual taking part in the study in all documents required for the study, or by erasing any personal information using a suitable method (e.g., correction fluid) from the source documents to be included in the study documentation.

The electronic data file will be declared to the CNIL in accordance with the appropriate procedure for the case.

IX - STATISTICAL DATA MANAGEMENT

IX.1 - RESPONSIBILITY FOR THE DATA ANALYSIS AND SOFTWARE USED

The statistical analysis will be carried out independently by a blinded statistician from the Epidemiology, Biostatistics and Clinical Research Department of the Cochin Hôtel-Dieu Broca Hospital Group, using Statistical Analysis System version 9.1, under the responsibility of Prof. Isabelle Boutron and Prof. Philippe Ravaud. A statistical analysis plan will be prepared and validated prior to the freezing of the database and the unblinding.

The health economics evaluation will be carried out by Prof. Isabelle Durand-Zaleski.

IX.2 - JUSTIFICATION OF THE REQUIRED NUMBER OF SUBJECTS

The first primary endpoint is the percentage of patients able to be discharged from the surgical department on D4, plus or minus 1 day. The expected percentage in the control arm is 20%. The sample size of 130 patients per arm will allow for an odds ratio of 2.15 (logrank test) with a power of 90% for a p-value of 2.5% in bilateral formulation in order to take into account the double endpoint. The second primary endpoint is the WOMAC function area under the curve analysis at 6 months. The total number of 260 subjects enrolled (130 per group) will allow to demonstrate an effect size of 0.45 for comparison of the average areas under the curve with a power equal to 90% (p-value equal to 2.5%, Mann-Whitney test). Taking into account approximately 10% losses-to-follow-up before the procedure, the number of patients to be enrolled into each study arm will be approximately 150, giving a total of 300.

IX.3 - ANALYSIS STRATEGY FOR COLLECTED DATA

An intention-to-treat analysis will be carried out. All randomised patients will be analysed and each patient will be analysed in the group to which they have been randomised, regardless of the technique received.

IX.4 - JUSTIFICATION OF STATISTICAL TESTS

1. Descriptive statistics

The descriptive statistics will be presented in the form of absolute and relative frequencies for the qualitative variables and means with standard deviation and range for the quantitative variables.

2. Primary endpoint analysis

a) The percentage of patients able to be discharged from the surgical department on D4, plus or minus 1 day, will be compared between the 2 groups using the Chi-squared test. Furthermore, analyses adjusted for possible confounding factors (such as patient weight) will also be carried out using a logistic regression model. In addition, an operator effect will be added secondarily to the model to take into account the correlation between the time periods observed in patients operated on by the same surgeon in the event that there is an operator effect present. The results of the adjusted models will be compared to the non-adjusted model.

b) The WOMAC area under the curve between enrolment and 6 months will be calculated using the trapezoidal rule. The mean areas under the curve will be compared using the non-parametric Mann-Whitney test. In the event of missing data after the initial visit, an area under the curve of 1 will be used.

The endpoint tests will be considered significant at the 2.5% threshold so as to take into account the double primary endpoint.

3. Secondary endpoint analysis

The long-term analysis of the quantitative efficacy criteria repeated over time (personalised WOMAC, numeric pain scale, SF12, number of steps, satisfaction) will be carried out using a linear mixed model for repeated measurements (MMRM) taking into account the correlation of the measurements repeated in the same subject (random effect). The fixed effects will be the randomisation arm, operator, time, initial endpoint value and interaction between the time and the randomisation arm. The model will therefore allow us to compare the means adjusted to the absolute variations between the different times of interest (preoperative, 6 months, 1 year) and the initial visit. This analysis technique is consistent with the principle of the intention-to-treat analysis provided that all patients have a baseline value reported for the endpoint.

4. Health economics evaluation

This is a prospective study in which resource consumption by the patients in each arm will be collected in the case report form and evaluated 1) from the Health Insurance perspective, based on the reference nomenclature and prices in force at the end of the study, and 2) from the care-system perspective using the available hospital accounting data.

The resources collected include:

- time period before the intervention by the surgeons in the control group, and by the teams in charge of education and rehabilitation management in the intervention group;
- length of hospital stay and GHM (or the components of the medical unit summary) corresponding to the stay for the intervention;
- length of stay at rehabilitation care facilities;
- consultations and nursing or physiotherapy procedures during the follow-up period;
- imaging studies and medications consumed during follow-up;
- length of time off work or the time taken to resume normal activity.

There are, a priori, no structure costs related to the intervention as this uses existing capacities. However, we can consider designing a budgetary impact and an impact in terms of therapeutic education professionals if this intervention was to be widely used.

Pricing:

From the Health Insurance perspective: in the control group, the preoperative consultation by the surgeon will be priced at the key-letter rate. The education sessions in the intervention group are not currently priced in the Common Classification of Medical Procedures (CCAM), but we could potentially, by analogy with the pricing of education sessions for diabetic patients, propose 5C for sessions. Admissions to Medical, Surgical and Obstetric (MCO) units will be priced at the GHM rate with any additional supplements (length of stay, mainly). Admissions to rehabilitation care facilities will be priced according to the rates in force at the end of the

study, a fixed daily rate or GHM. Other outpatient consumptions will be priced according to the nomenclature rates corresponding to the reference products for medications. With regard to periods of sick leave for individuals who work, these will be priced in line with the average daily allowance amount.

From the care-system perspective: the main difference is that we are seeking to identify which workload transfers between the acute sector (MCO) and the rehabilitation sector would result from the implementation of an intensive preoperative education programme. Indeed, we can speculate that this programme would enable patients to be discharged earlier (which is neutral in terms of cost from the Health Insurance perspective but not from the hospital perspective) and therefore increase the orthopaedic department activity. Based on the analyses carried out by the hospital medical information departments, we can find out if, on average, a department's revenue is higher than its expenditure. It is therefore possible to determine (again, on average) whether or not a one-day reduction in the length of hospital stay would enable the department to generate revenue in relation to increased activity. The rehabilitation care facility perspective is the simplest as the cost of the intervention involved in the study is equal to the salaries of the individuals involved. Given the absence of pricing for education sessions, there is not currently any revenue to compare.

The calculation from the care-system perspective is therefore particularly interesting as it involves both the financial balance within the orthopaedics department and a potential public decision regarding pricing for the rehabilitation care facility sector.

Health economics evaluation.

The intervention costs (from the Health Insurance perspective for the basic calculation) will be compared. In the event that the most expensive strategy is the one which provides the best functional outcome and quality of life, we will carry out a cost-effectiveness analysis. The values will be taken from the EuroQol questionnaire. The base ratio estimation will be completed with a sensitivity analysis (particularly on the pricing of education procedures) and a bootstrap estimation of confidence intervals.

X - SAFETY ASSESSMENT

X.1 - DEFINITIONS

According to Article R1123-39 of the French Public Health Code:

- **Adverse event:**

  Any harmful manifestation occurring in an individual taking part in a biomedical research study, regardless of whether or not the manifestation is related to the study.

- **Adverse event** in a study not involving a product mentioned in Article L.5311-1 (medicinal products, biomaterials and medical devices, in vitro diagnostic medical devices, labile blood products, organs, tissues, cells and products of human or animal origin, and cellular products for therapeutic purposes).

Any adverse event due to the study.

- **Serious adverse event or effect:**

  Any adverse event or effect that leads to death, is life-threatening for the individual taking part in the study, involves hospital admission or an extended hospital stay, causes significant or permanent incapacity or disability, or leads to a congenital abnormality or malformation.

- **New information:**

  Any new safety information that may lead to a reassessment of the risk-benefit ratio of the study, or which may be enough to consider making amendments to study-related documents, to the way the study is conducted and, where applicable, to the way the product is used.

X.2 - DESCRIPTION OF THE SAFETY ASSESSMENT PARAMETERS

The study-specific procedures are as follows:

- 4 exercise sessions;
- 4 therapeutic education sessions;
- preoperative assessment;
- daily assessment of discharge ability from D1 to D5;
- self-exercise programme and DVD;
- follow-up questionnaires at 6 weeks, 4, 5 and 6 months (by post, email or telephone).

This is a multidisciplinary programme combining therapeutic education and exercises, with the exercise intensity being adapted to the patient's pathology and age, under the supervision of a physiotherapist. We have no known or reported factors regarding the risks related to the education programme.

The adverse events that may occur during the study are mainly related to the assigned treatment strategies.

- Main adverse events related to the education programme
  - ✓ no known or reported evidence on this subject
- Main adverse events related to the exercise programme
  - ✓ increase in exercise-related pain

- ✓ exercise-related injuries to muscles (cramps, elongation), ligaments (sprain) and joints (effusion). These are minor, non-progressive injuries which are quick to heal, as the exercise intensity will be adapted to the patient's pathology and age, under the supervision of a physiotherapist.

If any events occur, the patients will be assessed by the doctor in charge of the study for their treatment.
The study doctor will record adverse events (AEs) from the date on which the informed consent form is signed, for the whole duration of the patient's participation in the study, on the adverse events page of the case report form.

As this is a biomedical research study classified as "risk A", i.e. for which there is a negligible **additional risk expected for the study**, no **serious** adverse events are expected through the course of the study.

X.3 - SERIOUS ADVERSE EVENT MANAGEMENT PROCEDURE

The adverse event reporting table will be included as an annex to the protocol. It will include:

- the list of SAEs which do not have to be reported to the sponsor: anything related to the usual natural course of knee osteoarthritis (worsening of the condition, elective and non-elective admission to monitor the condition); any serious adverse event which may be related to treatments prescribed as part of care during the study follow-up period; any other medical event apart from knee osteoarthritis, not related to the additional procedures required for this study;
- The list of AEs expected during the study;
- The reference to "none", in the column of serious adverse events expected during the study.

Only SAEs directly related to the additional procedures required for this study are to be reported to the sponsor <u>immediately, from the day on which the investigator becomes aware of them</u>.
In the event that the investigator becomes aware of an event that could affect the safety of any individual involved in the study (e.g., therapeutic error or protocol deviation), he or she will be required to report this to the sponsor using the form provided for this purpose and attached as an annex to the protocol.

The investigator will fill in the serious adverse event report form (from the study case report form) (see annex) and send it to the DRCI Vigilance Unit by fax to (+33) 01 44 84 17 99 (if

possible, after immediately calling (+33) 01 44 84 17 23 in the event of unexpected death or a life-threatening event).

For each serious adverse event, the investigator must issue an opinion regarding the causal relationship of the event with any additional procedures specifically required for the study.

Obtaining information regarding the description and evaluation of an adverse event may not be possible within the time limit for the initial reporting. Furthermore, the clinical course and results of any clinical work-ups and diagnostic and/or laboratory studies, or any other information enabling a proper analysis of the causal relationship, will be reported:
either with the initial SAE report if they are immediately available;
or at a later date, as soon as possible, by sending a new SAE report form by fax (specifying that this is a follow-up to a previously reported SAE and providing the follow-up number).

In the event of death reported for a subject taking part in the study, the investigator will provide the sponsor with all the additional information requested (hospital discharge summary, autopsy results, etc.).

Any new information occurring in the study or in the context of the study, from data in the literature or ongoing studies, must be reported to the sponsor.

- Declaration of serious adverse events to the Health Authorities
The sponsor continuously assesses the safety of the individuals taking part in the study for its whole duration.
It assesses the severity of all adverse events reported to it by investigators, the causal relationship with each of the additional procedures required by the study and the unexpected nature of the adverse events.
Any suspected unexpected serious adverse events due to one of the study procedures or to the study itself will be reported by the sponsor to the competent authorities and to the Ethics Committee within the deadlines set forth by law.

The sponsor will inform the investigators involved about any information which could have an unfavourable impact on the safety of the individuals taking part in the study.

The sponsor will prepare an annual safety report for the entire duration of the study, which will be sent to the competent authorities and to the Ethics Committee within the deadlines set forth by law.

X.4 - STUDY-SPECIFIC COMMITTEES

X.4.1 - STEERING COMMITTEE

The steering committee will be made up of the Principal Investigator, Prof. François Rannou, investigators from the various sites, Prof. Emmanuel Coudeyre, Prof. Pascal Richette, Prof. Philippe Anract, Prof. David Biau, Prof. Rémy Nizard, Prof. Johann Beaudreuil, Prof. Stéphane Boisgard, the methodologists in charge of the project, Prof. Isabelle Boutron, Prof. Philippe Ravaud, Prof. Isabelle Durand-Zaleski, one or more Project Advisers from the Clinical Research Department (DRCI), the heads of the CIC Cochin Necker Clinical Research Unit (URC), Prof. Jean-Marc Tréluyer, and one or more Project Advisers from the CIC Cochin Necker Clinical Research Unit (URC).

The roles of the steering committee are as follows:

- to define the general organisation and implementation of the study and coordinate the information;
- to initially define the methodology and decide on the measures to be taken throughout the course of the study if any unexpected events occur;
- to supervise the implementation of the study, particularly in terms of tolerance and adverse events.

X.4.2 - INDEPENDENT MONITORING COMMITTEE

The implementation of an independent monitoring committee is not planned for this study. The absence of a monitoring committee is justified by the fact that there is no expected risk.

XI - RIGHT TO ACCESS THE INFORMATION AND SOURCE DOCUMENTS

Individuals with direct access in accordance with the legislative and regulatory provisions in force, in particular Articles L.1121-3 and R.5121-13 of the French Public Health Code (e.g., researchers, individuals in charge of quality control, monitors, clinical research assistants, auditors and all individuals collaborating in clinical trials) will take all the necessary precautions in order to ensure the confidentiality of the information related to the investigational medicinal products, trials, individuals taking part in the study and, in particular,

any information involving their identity, as well as the results obtained. The data collected by these individuals through the course of quality controls or audits will then be made anonymous.

XII - LEGAL AND ETHICS ASPECTS

The sponsor is defined by Law 2004-806 of 9 August 2004. AP-HP is the sponsor of this study and the Delegation of Clinical Research and Innovation (DRCI) undertakes the regulatory tasks. Before beginning the study, each investigator must provide the sponsor's representative in the study with a signed and dated copy of their curriculum vitae, which must include their French National Medical Council registration number.

XII.1 - REQUEST FOR AUTHORISATION FROM THE ANSM

Before beginning the study, AP-HP, as the sponsor, must submit an authorisation request file to the competent authority (the ANSM). The competent authority, as defined in Article L.1123-12, makes decisions related to the safety of individuals taking part in a biomedical research study, taking into account the safety and quality of the products used during the study in accordance with the regulations in force, where applicable, their condition of use and the safety of individuals with regard to procedures carried out and the methods used, as well as the planned methods of patient follow-up.

XII.2 - REQUEST FOR OPINION FROM THE ETHICS COMMITTEE

In accordance with Article L.1123-6 of the French Public Health Code, the sponsor must submit the study protocol to an Ethics Committee. The committee's opinion will be reported to the competent authority by the sponsor before the study begins.

XII.3 - AMENDMENTS

The DRCI must be informed of any planned changes to the protocol by the principal investigator.

Amendments must be classified as substantial or non-substantial. A substantial amendment is an amendment which may, in one way or another, change the guarantees given to the individuals taking part in the biomedical research (change in inclusion criteria, extension of enrolment period, participation of new sites, etc.).

Once the study has started, any substantial amendments proposed by the sponsor must receive a favourable opinion from the ethics committee and authorisation from the competent authority prior to being implemented. In this case, where necessary, the committee will ensure that a new consent form is duly collected from individuals taking part in the study.

Moreover, any extension to the study (radical change in the treatment regimen or populations included, extension of treatments and/or therapeutic procedures not originally foreseen in the protocol) must be considered as a new study.
Any substantial amendment must be submitted **by the sponsor** for authorisation from the ANSM and/or for the ethics committee's opinion.

XII.4 - CNIL DECLARATION

The law sets forth that the declaration of the electronic file with the personal data collected for the study must be prepared before the effective start of the study.
A reference methodology specific to the processing of personal data carried out in the context of biomedical research studies, defined by Law 2004-806 of 9 August 2004 as falling within the scope of Articles L.1121-1 et seq. of the French Public Health Code, was established by the CNIL in January 2006. This methodology allows a simplified declaration procedure when the nature of the data collected in the study is consistent with the list provided by the CNIL in its reference document. When the protocol undergoes a quality control of the data by a CRA representing the sponsor and falls within the scope of the simplified CNIL procedure, the DRCI as the sponsor will ask the person in charge of the electronic file to undertake in writing to comply with the simplified MR06001 reference methodology.

XII.5 - INFORMATION SHEET AND INFORMED CONSENT FORM

Written consent must be collected from any individual taking part in the study before any procedures related to the biomedical research are performed.
Within the context of this study, the patients will be enrolled at the visit with the orthopaedist, the rheumatologist or the rehabilitation physician (enrolment visit). During this visit, the patient will be informed of all of the study-related information. At the end of the enrolment visit, if the patient meets all the eligibility criteria and agrees to participate in the study, he or she will provide the physician with the signed consent form.

XII.6 - FINAL STUDY REPORT

The final study report will be prepared by the principal investigator in collaboration with the biostatistician for this study. This report will be submitted to each of the investigators for their opinion. Once a consensus has been reached, the final version must be approved with the signature of each of the investigators and sent to the sponsor as soon as possible after the effective end of the study. A report prepared in accordance with the competent authority reference plan must be sent to the competent authority and to the ethics committee within one

year after the end of the study, with the end of the study understood to be the last follow-up visit of the last subject enrolled. This period is set at 90 days if the study is terminated early.

XIII - DATA PROCESSING AND STORAGE OF STUDY-RELATED DOCUMENTS AND DATA

The documents from a study falling under the scope of the law on biomedical research must be archived by all the parties for a period of 15 years after the end of the study (see GCP, chapter 8: essential documents).

This indexed archive consists of:

- Copies of the ANSM authorisation letter and the mandatory opinion from the Ethics Committee;
- Subsequent versions of the protocol (identified by the version number and date);
- Letters of correspondence with the sponsor;
- The informed consent forms signed by the subjects in a sealed envelope (in the case of minor subjects, these are signed by their parents or guardians) with the corresponding list or enrolment register;
- The complete and validated case report form for each subject enrolled;
- Any specific annexes to the study;
- The final study report from the statistical analysis and the quality control of the study (sent in duplicate to the sponsor);
- Certificates from any audits performed during the course of the study.

The database used for the statistical analysis must also be archived by the head analyst (hard copy or electronic copy).

XIV - INSURANCE AND SCIENTIFIC COMMITMENT

XIV.1 - INSURANCE

Assistance Publique - Hôpitaux de Paris is the sponsor of this study. In accordance with the law on biomedical research studies, it has taken out an insurance policy with the company HDI - GERLING for the full duration of the study, guaranteeing its own civil liability as well as that of any intervening parties (physicians or staff involved in conducting the study) (Law no. 2004-806, Art. L.1121-10 of the French Public Health Code).

Assistance Publique - Hôpitaux de Paris reserves the right to interrupt the study at any given time for medical or administrative reasons. If this occurs, the investigator will be notified.

XIV.2 - PRINCIPAL INVESTIGATOR'S COMMITMENT

Each investigator undertakes to comply with the obligations of the law and to conduct the study in accordance with the GCP guidelines, complying with the principles set forth in the Declaration of Helsinki in force. To this end, a copy of the scientific commitment (DRCI document), dated and signed by the principal investigator of each clinical department of a participating site, will be provided to the sponsor's representative.
A task delegation form will be filled in, dated and signed by all study collaborators.

XV - RULES REGARDING PUBLICATION

AP-HP owns the data and it may not be used or transferred to third parties without AP-HP's prior agreement.
The individuals who actively participated in preparing and implementing the protocol, as well as writing up the results, will be named first in the publications.
Assistance Publique-Hôpitaux de Paris must be mentioned as the sponsor of the biomedical research study and as a provider of funding, where applicable. "Assistance Publique-Hôpitaux de Paris" must appear in the address of the authors.

XVI - LIST OF ANNEXES

Annex 1: Literature references

Annex 2: Investigators and Associate Teams

Annex 3: WOMAC Questionnaire Functional Scale

Annex 4: MOS SF-12 Quality of life questionnaire

Annex 5: Quality of life questionnaire: EQ-5D-3L

Annex 6: RAPT (Risk Assessment and Prediction Tool)

Annex 7: Multidisciplinary programme procedure: Therapeutic education

Annex 8: Multidisciplinary programme procedure: Exercise sessions

Annex 9: Pre-TKA self-exercise programme

Annex 10: Pedometer use

Annex 11: Knee arthroplasty guide

Annex 12: Table for Classification of adverse events for a biomedical research study not involving a medicinal product, assimilated product, medical device or genetics

Annex 13: Serious adverse event reporting form

ANNEX 1 – LITERATURE REFERENCES

1. Ackerman IN, Benell KL. Does pre-operative physiotherapy improve outcomes from lower limb joint replacement surgery, A systematic review. Aust J Phys 2004; 50: 25-30.

2. Altman DG, Schulz KF, Moher D, Egger M, Davidoff F, Elbourne D, Gotzsche P, Lang T. The revised CONSORT statement for reporting randomized trials: explanation and elaboration. Ann Intern Med 2001; 134: 663-694.

3. Baecke JAH, Burema J, Frijters JER. A short questionnaire for the measurement of habitual physical activity in epidemiological studies. Am J Clin Nutr, 1982; 36: 936-42.

4. Beaupre L, Lier D, Davies DM, Johnston DBC. The effect of a preoperative exercise and education program on functional recovery, health related quality of life, and health service utilization following primary total knee arthroplasty. J Rheumatol 2004; 31: 1166-73.

5. Boutron I, Moher D, Altman DG, Schulz KF, Ravaud P for the CONSORT Group. Methods and processes of the CONSORT Group: Example of an extension for trials assessing nonpharmacologic treatments. Ann Inter Med 2008; 148: 295-309.

6. Brooks R, the EuroQol Group. EuroQol: the current state of play. Health Policy; 1996; 37: 53-72.

7. Coudeyre E, Jardin C, Givron P, Ribinik P, Revel M, Rannou F. Quel est l'intérêt d'une rééducation avant la pose d'une prothèse totale de hanche ou de genou ? Elaborations de recommandations françaises pour la pratique clinique. Ann Readapt Med Phys; 2007; 50: 189-97.

8. Craig P, Dieppe P, Macintyre S, Mitchie S, Nazareth I, Petticrew M. Developing and evaluating complex interventions: the new Medical Research Council guidance. BMJ 2008; 337: 979-983.

9. Crowe J, Henderson J. Pre-arthroplasty rehabilitation is effective in reducing hospital stay. Can J Occup Ther 2003; 70: 88-96.

10. Dowsey MM, Kilgour ML, Santamaria NM, Choong PF. Clinical pathways in hip and knee arthroplasty: a prospective controlled study. Med J Aust; 1999; 170(2): 59-62

11. D'Lima DD, Colwell CW, Morris BA, Hardwick ME, Kozin F. The Effect of Preoperative Exercise on Total Knee Replacement Outcomes. Clin Orthop Relat Res; 1996; 326: 174-182.

12. Fedmer. Critères de prise en charge en Médecine Physique et Réadaptation Sofmer Bordeaux 2001. Available at http://www.cpod.com/monoweb/fedmer/criteresPEC/index.htm

13. Fortin PR, Clarke AE, Joseph L et al. Outcomes of total hip and knee replacement: preoperative functional status predicts outcomes at six months after surgery. Arthritis Rheum. 1999 ; 42: 1722-8.

14. Fransen M, Edmonds J. Reliability and validity of the EuroQol in patients with osteoarthritis of the knee. Rheumathology; 1999; 38: 807-13.

15. Fransen M, McConnell S, Bell M. Exercise for osteoarthritis of the hip or knee. Cochrane Database Syst Rev. 2003; (3): CD004286.

16. Ganz SB, Wilson PD Jr, Cioppa-Mosca J, Petarson MG. The day of discharge after total hip arthroplasty and the achievement of rehabilitation functional milestones: 11-year trends. J Arthroplasty; 2003; 18(4):453-7.

17. Gilbey HJ, Ackland TR, Wang AW, Morton AR, Trouchet T, Tapper J. Exercise Improves Early Functional Recovery After Total Hip Arthroplasty. Clin Orthop Relat Res 2003; 408; 193-200.

18. HAS. HAS recommendations established by formal consensus, regarding surgical and orthopaedic procedures which do not generally require (for a patient qualifying for massage-physiotherapy treatment) admission for the purpose of providing follow-up care and rehabilitation, as mentioned in Article L.6111-2 of the French Public Health Code. 2006 Paris, France.

19. Hochberg MC, Altman RD, Brandt KD et al. Guidelines for the medical management of osteoarthritis. Part I. Osteoarthritis of the hip. American College of Rheumatology. Arthritis Rheum. 1995 ; 38: 1535-40.

20. Hochberg MC, Altman RD, Brandt KD, Clark BM et al. Guidelines for the medical management of osteoarthritis. Part II. Osteoarthritis of the knee. American College of Rheumatology. Arthritis Rheum. 1995 ; 38: 1541-6.

21. Canadian Institute for Health Information. 2006 report. Hip and knee arthroplasties in Canada. Canadian Joint Replacement Registry (CJRR). Canadian Institute for Health Information, Ontario, 2006, pp 12, 25.

22. Kraaimaat FW, Evers AWM. Pain-coping strategies in chronic pain patients: psychometric characteristics of the Pain-Coping Inventory (PCI). Int J Behav Med 2003; 10: 343-63.

23. Kurtz SM, Ong KL, Lau E, Mowat F, Halpern M. Projections of primary and revision hip and knee arthroplasty in the United States from 2005 to 2030. J Bone Joint Surg Am. 2007;89(4): 780-5

24. Kwoh CK, Petrick MA, Munin MC. Inter-rater reliability for function and strength measurements in the acute care hospital after elective hip and knee arthroplasty. Arthritis Care Res; 1997; 10(2); 128-34.

25. Mathias S, Nayak US, Isaacs B. Balance in elderly patients: the “get up and go” test. Arch Phys Med Rehabil; 1986; 67: 387-89.

26. McDonald S, Green SE, Hetrick S. Pre-operative education for hip or knee replacement. The Cochrane Database of Systematic Reviews 2004, Issue 1. Art. No.: CD003526.

27. Mitchell C, Walker J, Walters S, Morgan AB, Binns T, Mathers N. Costs and effectiveness of pre- and post-operative home physiotherapy for total knee replacement: randomized controlled trial. J Eval Clin Pract. 2005; 11: 283-92.

28. Mouthon L, Rannou F, Bérezné A, Pagnoux C, Guilpain P, Goldwasser F, Revel M, Guillevin L, Fermanian J, Poiraudeau S. Patient preference disability questionnaire in systemic sclerosis: a cross-sectional survey. Arthritis Rheum 2008; 59: 968-73.

29. Munin MC, Rudy TE, Glynn NW, Crossett LS, Rubash HE. Predicting discharge outcome after elective hip and knee arthroplasty. Am J Phys Med Rehabil; 1995; 74(4): 294-301

30. Munin MC, Rudy TE, Glynn NW, Crossett LS, Rubash HE. Early inpatient rehabilitation after elective hip and knee arthroplasty. Jama; 1998; 279(11):847-52

31. Munjanja SP Lindmark G, Nyström L Randomized controlled trial of a reduced-visits programme of antenatal care in Harare, Zimbabwe, Lancet 1996, 348:364-369

32. Pendleton A, Arden N, Dougados M, Doherty M et al. EULAR recommendations for the management of knee osteoarthritis: report of a task force of the Standing Committee for International Clinical Studies Including Therapeutic Trials (ESCISIT). Ann Rheum Dis; 2000; 59: 936-44.

33. Pereira MA, FitzerGerald SJ, Gregg EW, Joswiak ML, Suminski RR, Utter AC, Zmuda JM. A collection of physical activity questionnaires for health-related research. Med Sci Sports Exerc. 1997; 29 (6 suppl): S1-205.

34. Perrot S, Poiraudeau S, Kabir M, Bertin P, Sichere P, Serrie A, Rannou F. Active or passive pain coping strategies in hip and knee osteoarthritis? Results of a national survey of 4,719 patients in a primary care setting. Arthritis Rheum; 2008; 59: 1555-62.

35. Philadelphia Panel. Philadelphia Panel evidence-based clinical practice guidelines on selected rehabilitation interventions for knee pain. Phys Ther; 2001; 81: 1675-1700.

36. Philippaerts RM, Westerterp KR, Lefevre J. Doubly labelled water validation of three physical activity questionnaires. Int J Sports Med; 1999; 20: 284-89.

37. Podsiadlo D, Richardson S. The timed “Up & Go”: a test of basic functional mobility for frail elderly persons. J Am Geriatr Soc; 1991; 39: 142-48.

38. Ravaud P, Flipo R-M, Boutron I, Roy C, Mahmoudi A, Giraudeau B, Pham T. The ARTIST (Osteoarthritis Intervention Standardized) study: A pragmatic randomised controlled trial comparing standardized consultation to usual care for patients with knee osteoarthritis under primary care. BMJ 2008 in Press.

39. Roddy E, Zhang W, Doherty M et al. Evidence-based recommendations for the role of exercise in the management of osteoarthritis of the hip or knee - the MOVE consensus. Rheumatology (Oxford) 2005; 44: 67-73.

40. Seror R, Tubach F, Baron G, Falissard B, Logeart I, Dougados M, Ravaud P. Individualising the Western Ontario and McMaster Universities osteoarthritis index (WOMAC) function subscale: incorporating patient priorities for improvement to measure functional impairment in hip or knee osteoarthritis. Ann Rheum Dis; 2008; 67: 494-99.

41. Tugwell P, Bombardier C, Buchanan WW, Goldsmith CH, Grace E, Hanna B. The MACTAR patient preference disability questionnaire – an individualized functional priority approach for assessing improvement in physical disability in clinical trials in rheumatoid arthritis. J Rheumatol 1987; 14: 446-51.

42. Verhoeven AC, Boers M, Van Der Liden S. Validity of the MACTAR questionnaire as a functional index in a rheumatoid arthritis clinical trial. The McMaster Toronto Arthritis. J Rheumatol 2000; 27: 2801-9.

43. Viton JM, Atlani L, Mesure S, Franceschi JP, Massion J, Delarque A, Bardot A. Reorganization of equilibrium and movement control strategies in patients with knee arthritis. Scand J Rehabil Med. 1999; 31: 43-8.

44. Walker WC, Keyser-Marcus LA, Cifu DX, Chaudhri M. Inpatient interdisciplinary rehabilitation after total hip arthroplasty surgery: a comparison of revision and primary total hip arthroplasty. Arch Phys Med Rehabil; 2001; 82(1): 129-33.

45. Wang AW, Gilbey HJ, Ackland TR. Perioperative exercise programs improve early return of ambulatory function after total hip arthroplasty: A randomized, controlled trial. Am J Phys Med Rehabil 2002; 81: 801-806.

46. Weidenheim L, Mattsson E, Brostrom LA, Wersallbrobertsson E. Effect of pre-operative physiotherapy in unicompartmental prosthetic knee replacement. Scand J Rehabil Med 1993; 25: 33-39.

47. Zavadak KH, Gibson KR, Whitley DM, Britz P, Kwoh CK. Variability in the attainment of functional milestones during the acute care admission after total joint replacement. J Rheumatol; 1995; 22(3): 482-7.

48. Zhang W, Doherty M, Arden N et al. EULAR evidence based recommendations for the management of hip osteoarthritis: report of a task force of the EULAR Standing Committee for International Clinical Studies Including Therapeutics (ESCISIT). Ann Rheum Dis. 2005 ; 64: 669-81.

49. Zhang W, Doherty M. EULAR recommendations for knee and hip osteoarthritis: a critique of the methodology. Br J Sports Med 2006; 40: 664-9.

ANNEX 2 – INVESTIGATORS AND ASSOCIATE TEAMS

Site no.	Hospital and Department	Principal Investigators
01	**Cochin Hospital**<br>27 rue du Faubourg St Jacques<br>75679 Paris Cedex 14<br><br>**Department of Physical Medicine and Rehabilitation of the Musculoskeletal System and Spinal Disorders (PMR)**	**Prof. François Rannou**
02	**Clermont-Ferrand University Hospital**<br>Bd Leon Malfreyt<br>63058 Clermont-Ferrand France<br><br>**Rehabilitation Department**	**Prof. Emmanuel Coudeyre**<br>(Rehabilitation Specialist)
03	**Lariboisière Hospital**<br>2 rue Ambroise Paré<br>75010 Paris<br><br>**Rheumatology Department**	**Prof. Pascal Richette**<br>(Rheumatologist)

ANNEX 3 – WOMAC QUESTIONNAIRE FUNCTIONAL SCALE

Function Section

For each of the following activities, please specify the difficulties experienced due to the affected joint, over the past 48 hours.

**How significant is the difficulty you experienced when:**	**None**<br>**0**	**Minimal**<br>**1**	**Moderate**<br>**2**	**Severe**<br>**3**	**Very severe**<br>**4**
1. Going down stairs	❑	❑	❑	❑	❑
2. Going up stairs	❑	❑	❑	❑	❑
3. Standing up from the seated position	❑	❑	❑	❑	❑
4. Standing	❑	❑	❑	❑	❑
5. Leaning forward	❑	❑	❑	❑	❑
6. Walking on level ground	❑	❑	❑	❑	❑
7. Getting in and out of the car	❑	❑	❑	❑	❑
8. Doing the shopping	❑	❑	❑	❑	❑
9. Putting on tights or socks	❑	❑	❑	❑	❑
10. Getting out of bed	❑	❑	❑	❑	❑
11. Taking off tights or socks	❑	❑	❑	❑	❑
12. Lying down in bed	❑	❑	❑	❑	❑
13. Getting in or out of the bath	❑	❑	❑	❑	❑
14. Sitting down	❑	❑	❑	❑	❑
15. Sitting down on and standing up from the toilet	❑	❑	❑	❑	❑
16. Cleaning the house	❑	❑	❑	❑	❑
17. Carrying out daily housework	❑	❑	❑	❑	❑

Calculation = (sum/17) x 25

Total 0-100 = |_|_|_|_|_|_|

Personalised WOMAC Section

Please choose (from the 17 items in the previous list) the 5 most important items for which you would like to see an improvement.

1.- ……………………………………………………………
2.- ……………………………………………………………
3.- ……………………………………………………………
4.- ……………………………………………………………
5.- ……………………………………………………………

Calculation = (sum/5) x 25

Total 0-100 = |_|_|_|_|_| |_|_|

ANNEX 4 – MOS SF-12 QUALITY OF LIFE QUESTIONNAIRE

The following questions ask for your views about your health. Your answers will help monitor your health condition and to know how well you are able to carry out your usual activities.
Answer all of the following questions by following the instructions you have been given. If you are unsure, please give the best answer you can.

1. **In general, would you say your health is:** *(mark one answer only)*

- Excellent.. ☐
- Very good .... ☐
- Good.. ☐
- Fair .... ☐
- Poor .... ☐

The following is a list of activities you might do during a typical day. For each of these, indicate whether your current health condition limits you in these activities.

(mark one answer only per line)

	Very limited	Somewhat limited	Not at all limited
2. **Moderate physical activities** such as moving a table, pushing a vacuum cleaner, bowling	☐	☐	☐
3. Climbing **several flights** of stairs	☐	☐	☐

During the <u>past 4 weeks,</u> due to your <u>physical condition</u>,

(mark one answer only per line)

	YES	NO
4. Have you **accomplished less** than you would have liked?	☐	☐
5. Have you had any **difficulties** in doing your job or any other activities (e.g. they have required an additional effort)?	☐	☐

During the <u>past 4 weeks</u>, due to your <u>emotional state</u> (such as feeling sad, nervous or depressed),

(mark one answer only per line)

	YES	NO
6. Have you **accomplished less** than you would have liked?	☐	☐
7. Have you had **difficulties** in doing what you had to do **with as much care and attention as usual?**	☐	☐

8. **During the past 4 weeks, how much did your physical pain interfere with your work or housework?**

(mark one answer only)

- -Not at all .... ☐
- -A little bit .... ☐
- -Moderately .... ☐
- -A lot .... ☐
- -Extremely ……………….................….. ☐

The following questions are related to how you have felt <u>during the past 4 weeks.</u> For each question, please indicate the response you feel is most appropriate.

<u>During the past 4 weeks,</u> have there been times when:-

(mark one answer only per line)

	All of the time	Most of the time	A good bit of the time	Some of the time	A little of the time	None of the time
9. You have felt calm and relaxed?	☐	☐	☐	☐	☐	☐
10. You have felt full of energy?	☐	☐	☐	☐	☐	☐
11. You have felt sad and discouraged?	☐	☐	☐	☐	☐	☐

12. **<u>During the past 4 weeks</u>, how much of the time has your <u>physical health or emotional problems</u> interfered with your social activities and relationships with others (family, friends, etc.)?**

(mark one answer only)

- -All of the time... ☐
- -Most of the time .... ☐
- -From time to time .... ☐
- -Rarely .... ☐
- -Never .... ☐

ANNEX 5 – QUALITY OF LIFE QUESTIONNAIRE: EQ-5D-3L

Please indicate, for each of the following sections, which statement best describes your health today, by ticking the corresponding box.

Mobility

I have no problems in walking about ☐

I have some problems in walking about ☐

I am confined to bed ☐

Self-care

I have no problems with self-care ☐

I have some problems washing or dressing myself ☐

I am unable to wash or dress myself ☐

Usual activities (*e.g. work, study, housework, family or leisure activities)*

I have no problems with performing my usual activities ☐

I have some problems with performing my usual activities ☐

I am unable to perform my usual activities ☐

Pain/Discomfort

I have no pain or discomfort ☐

I have moderate pain or discomfort ☐

I have extreme pain or discomfort ☐

Anxiety/Depression

I am not anxious or depressed ☐

I moderately anxious or depressed ☐

I am extremely anxious or depressed ☐

To help you indicate to what extent a health condition is good or bad, we have created a graduated scale (like a thermometer) on which 100 corresponds to the best health condition you can imagine and 0 corresponds to the worst health condition you can imagine.

Please indicate on this scale where you consider your health condition to be today. To do this, please draw a line from the box below to the point on the scale which corresponds to your health condition today.

Your health condition today

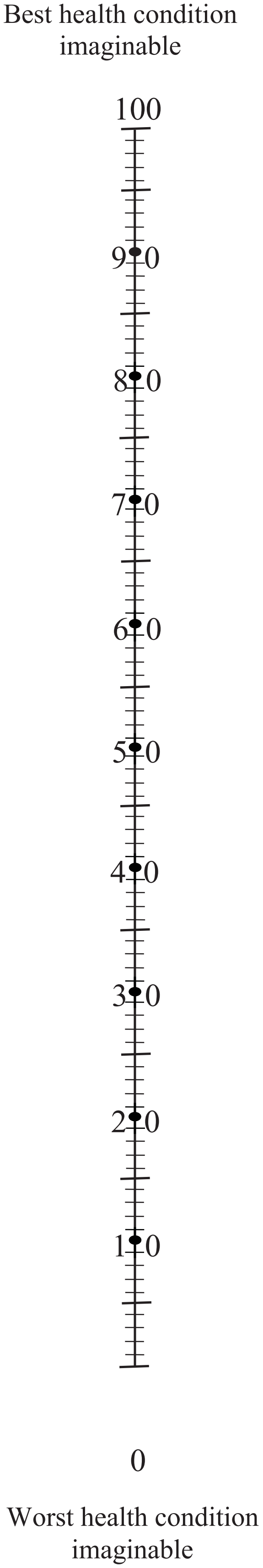

ANNEX 6 – RAPT (RISK ASSESSMENT AND PREDICTION TOOL)

RAPT index

	Data	Score
1. How old are you?	50-65 years old<br>66-75 years old<br>> 75 years old	*=2*<br>*=1*<br>*=0*
2. What is your sex?	Male<br>Female	*=2*<br>*=1*
3. How far on average can you walk?	More than 400 metres (with or without rest)<br>200 to 400 metres<br>Housebound (most of the time)	*=2*<br>*=1*<br>*=0*
4. Do you use a walking aid (more often than not)?	None<br>Single-point stick<br>Crutches or a frame	*=2*<br>*=1*<br>*=0*
5. Do you have external help?<br>(home help, meals on wheels, district nursing)	Never or once per week<br>Two or more times per week	*=1*<br>*=0*
6. Will you live with someone who can help you after your operation?	Yes<br>No	*=3*<br>*=0*
Total score out of 12		=

ANALYSIS

Destination at discharge from surgical department according to score.
Score < 6: Transfer to a Rehabilitation Care Facility
Score 6–9: Direct return home subject to additional perioperative care.
Score > 9: Direct return home

The patient's preference regarding orientation is also a crucial factor.
The orientation predicted by the score is discussed with the patient to decide on a discharge plan.

Patient preference	Predictive score	Final decision
…………………………..	……………………………..	……………………………..

ANNEX 12

Grille de Notification des Evénements Indésirables pour une Recherche Biomédicale
ne portant pas sur un produit de santé (Art. R. 1123-54 du Code de la Santé publique)

ASSISTANCE PUBLIQUE HÔPITAUX DE PARIS

EDEX Codes projet : P100122 – IDRCB 2012-A-00208-35 Risque de la Recherche : **A** CSI / DSMB : Oui ☐ **Non** ☒

« Evaluation d'un programme d'éducation associé à un programme d'exercices (EDEX) avant mise en place d'une prothèse totale de genou »

**A NE PAS NOTIFIER** AU PROMOTEUR<br>**Evénements recensés dans le protocole comme ne devant pas être notifiés mais qui pourront être recueillis dans le cahier d'observation (CRF)**		**A NOTIFIER SANS DELAI** AU PROMOTEUR<br>**Envoi du formulaire de notification d'EIG par fax au 01 44 84 17 99 et à recueillir dans le CRF**	
Événements pouvant être graves **mais non liés aux actes et procédures ajoutés spécifiquement par cette recherche**	**Effets Indésirables (EI) Non Graves ATTENDUS**<br>Connus pour être liés aux actes et procédures ajoutés spécifiquement par cette recherche	**Effets Indésirables Graves (EIG) ATTENDUS**	**Effets Indésirables Graves (EIG) INATTENDUS (SUSARs)**
<u>Description</u> :<br>➢ Tout ce qui est en rapport avec l'évolution naturelle et habituelle de la pathologie de la gonarthrose :<br>• aggravation de la maladie<br>• hospitalisation programmée ou non pour suivi de la pathologie<br>➢ Tout effet indésirable grave susceptible d'être lié aux traitements prescrits dans le cadre du soin pendant le suivi de la recherche<br>➢ Tout autre évènement médical en dehors de la pathologie de la gonarthrose, non lié aux actes et procédures ajoutés spécifiquement par cette recherche	<u>Description</u> :<br>➢ Liés au programme d'éducation thérapeutique :<br>• pas d'éléments connus ou rapportés<br>➢ Liés au programme d'exercices :<br>• augmentation des douleurs<br>• lésions musculaires (crampes, élongations), ligamentaires (entorses) et articulaires (épanchement) en rapport avec les exercices<br>Il s'agit de lésions bénignes, non évolutives avec cicatrisation rapide, car l'intensité de l'exercice sera adaptée à la pathologie et âge des patients, sous la supervision d'un kinésithérapeute. Devant la présence de ces évènements, les patients seront évalués par le médecin responsable de l'étude pour leur prise en charge.	**Aucun effet indésirable grave n'est attendu dans le cadre de cette recherche.**<br><br>**NEANT**	**Seuls les EIG directement liés aux actes et procédures ajoutés spécifiquement par cette recherche sont à notifier au promoteur, <u>sans délai à compter du jour où l'investigateur en a connaissance</u>.**

Nom, prénom et signature de l'investigateur coordonnateur :	Nom, prénom et signature du responsable de l'URC :	Nom, prénom et signature du référent projet :	Nom, prénom et signature du responsable Vigilance :	Nom, prénom et signature du coordonnateur médical :
Date :	Date :	Date :	Date :	Date :

10SPU-Edex_grille-vigilance_v2-0_20140331_DRCD

FORMULAIRE DE DECLARATION D'UN EVENEMENT INDESIRABLE GRAVE (EIG) SURVENANT AU COURS D'UNE RECHERCHE BIOMEDICALE NE PORTANT PAS SUR UN PRODUIT MENTIONNE A L'ARTICLE L.5311-1 DU CODE DE LA SANTE PUBLIQUE

ASSISTANCE PUBLIQUE HÔPITAUX DE PARIS

PARTIE RESERVEE AU PROMOTEUR : NE PAS REMPLIR
|_|_|_|-|_|_|_|- *DRCD* -|_|_|_|_|_|-|_|_|_|_|_|

Cette fiche doit être retournée dûment complétée (2 pages) au DRCD par fax : +33 (0)1 44 84 17 99
A l'attention de « *Moufida Dabbech* » et de « *Karine Goude* »

Date de notification : |_|_| jj |_|_| mm |_|_|_|_| aaaa

Code de la Recherche : P100122

N°IDRCB : 2012-A-00208-35 **Déclaration initiale** ☐ **Suivi d'EIG déclaré** ☐ **N°du suivi** |_|

Titre de la Recherche Biomédicale : *« Evaluation d'un programme d'éducation associé à un programme d'exercices (EDEX) avant mise en place d'une prothèse totale de genou »*

1) Nom et adresse du centre : ____________________

Centre n° : |_|_| Investigateur (Qualité - Nom - Prénom) : ____________________

2) Identification du patient :

Nom : |_| Prénom : |_|

Patient n° : |_|_|_|_|

Sexe : Masculin ☐ Féminin ☐

Date de naissance : |_|_| |_|_| |_|_|_|_|

Age : |_|_| ans

Poids : |_|_|_| kg

Taille : |_|_|_| cm

Date d'inclusion : |_|_| |_|_| |_|_|_|_|

Date de randomisation : |_|_| |_|_| |_|_|_|_|

Bras ____________ ☐ Bras ____________ ☐

3) Evénement indésirable grave :

Décès ☐

Mise en jeu du pronostic vital ☐

Nécessite ou prolonge l'hospitalisation : ☐
Du |_|_||_|_||_|_| *au* |_|_||_|_||_|_| ☐ *en cours*

Incapacité ou invalidité ☐

Anomalie congénitale ☐

Autre(s) critère(s) médicalement significatif(s) (préciser) : ☐

____

____

Antécédents (allergie, insuffisance rénale ...) : ____________________

____

4) Description complète de l'événement indésirable (diagnostic retenu, localisation anatomique, critères permettant de considérer l'événement comme grave) :

Intensité : Légère ☐ Modérée ☐ Sévère ☐

Date de survenue : |_|_| jj |_|_| mm |_|_|_|_| aaaa et heure de survenue : |_|_| hh |_|_| min

____

____

Délai de survenue : ____________________

5) Médicament(s) concomitant(s) à l'exclusion de ceux utilisés pour traiter l'événement indésirable :

Nom commercial (de préférence) ou Dénomination Commune Internationale	Voie	Dose/ 24h	Date de début	En cours	Date de fin	Indication	Causalité * (1,2,3 ou 4)
			|_|_||_|_||_|_|	☐	|_|_||_|_||_|_|		
			|_|_||_|_||_|_|	☐	|_|_||_|_||_|_|		
			|_|_||_|_||_|_|	☐	|_|_||_|_||_|_|		
			|_|_||_|_||_|_|	☐	|_|_||_|_||_|_|		

** 1 = Probable 2 = Possible 3 = Non liée 4 = Inconnue*

Identification du patient : Nom |__| Prénom : |__| Centre : |__|__| Code de la Recherche : P100122

6) Description du dispositif médical (DM) concomitant :

Nature	Modèle /type/référence	N°de série ou de lot	Nom du fournisseur	Nom du fabricant	Si DM stérile : Date de stérilisation et Date de péremption	Causalité* (1,2,3 ou 4)
					|__|__| |__|__| |__|__| et |__|__| |__|__| |__|__|	

** 1 = Probable 2 = Possible 3 = Non liée 4 = Inconnue*

7) Evolution (indiquez si des mesures symptomatiques ont été prises : non ☐ oui ☐ *Si oui, préciser*) :

8) Date de disparition : |__|__| jj |__|__| mm |__|__|__|__| aaaa et heure de disparition : |__|__| hh |__|__| min

9) Autre(s) étiologie(s) envisagée(s) : non ☐ oui ☐ *Si oui, préciser :*

10) Examen(s) complémentaire(s) réalisé(s) : non ☐ oui ☐ *Si oui, préciser date, nature et résultats :*

11) Selon l'investigateur, l'événement indésirable grave semble plutôt lié :

☐ à un dispositif médical posé
☐ à un (ou plusieurs) médicament(s) administré(s) : le(s)quel(s) : ____________
☐ aux procédures de la recherche biomédicale
☐ à une maladie intercurrente
☐ à la progression de la maladie
☐ autre : ____________

Date : |__|__| |__|__| |__|__| **Tampon du service :** **Nom de l'investigateur:** ____________ **Signature :**

Nom et fonction du Notificateur: ____________ **Téléphone** ____________ **Signature :**

PARTIE RESERVEE AU PROMOTEUR : NE PAS REMPLIR

***Numéro d'identification de l'événement :* EV |__|__|__|**

Date de réception par le promoteur : |__|__| |__|__| |__|__|__|__|

Date de ce rapport : |__|__| |__|__| |__|__|__|__| ☐ initial ☐ suivi n°|__|__|

Selon le promoteur, l'événement indésirable semble plutôt lié :

☐ à un dispositif médical posé
☐ à un (ou plusieurs) médicament(s) administré(s) : le(s)quel(s) :
☐ aux procédures de la recherche biomédicale
☐ à une maladie intercurrente
☐ à la progression de la maladie
☐ autre :

Si selon le promoteur, l'événement semble plutôt lié à la recherche biomédicale :

☐ L'événement indésirable grave est attendu ☐ L'événement indésirable grave est inattendu

Commentaires du promoteur : ____________

Nom et qualité du représentant du promoteur : **Signature :**

Clinical Epidemiology Centre

U1153 - METHODS Team

Statistical analysis plan, version no. 1.0

EDEX: Evaluation of an Educational Program Associated With Exercises (EDEX) Before Total Knee Arthroplasty

Registration number
NCT01671917

Principal Investigator
Prof. François Rannou
Cochin Hospital, Department of Physical Medicine and Rehabilitation of the Musculoskeletal System and Spinal Disorders, Paris

Drafted by Elodie Perrodeau
Prof. Isabelle Boutron
Prof. Philippe Ravaud
22 March 2018

Table of contents

1 Protocol summary **2**

2 Analysis population **4**
2.1 Patient flow description 4
2.2 Analysis populations definition 4
2.3 Calculation of the required number of subjects 5

3 Principles of analysis **5**
3.1 General principles for the endpoint analysis 5
3.2 Baseline patient characteristics 5
3.3 Compliance analysis 5
3.4 Management of missing data 5

4 Primary endpoint analysis **6**
4.1 Ability at discharge 6
5.1.1 Definition 6
5.1.2 Primary analysis 6
5.1.3 Sensitivity analyses 7
5.2 Speed of functional recovery 7
5.2.1 Definition 7
5.2.2 Primary analysis 7
5.2.3 Sensitivity analyses 7

6 Secondary endpoint analysis **7**
6.1 Knee pain over the past 48 hours 7
6.1.1 Definition 7
6.1.2 Analysis 7
6.2 Function 7
6.2.1 Definition 7
6.2.2 Analysis 8
6.3 Quality of life 8
6.3.1 Definition 8
6.3.2 Analysis 8
6.4 Number of steps 8
6.4.1 Definition 8
6.4.2 Analysis 8
6.5 Treatment satisfaction 8
6.5.1 Definition 8
6.5.2 Analysis 9

7 Tolerance analysis **9**
7.1 Adverse events 9
7.1.1 Definition 9
7.1.2 Analysis 9

1 Protocol summary

This document was written on the basis of the information contained in protocol version no. 5.0 dated 07/04/2017.

SUMMARY

**Title**	Evaluation of an Educational Program Associated With Exercises (EDEX) Before Total Knee Arthroplasty
**Principal Investigator**	Prof. François Rannou (Cochin Hospital)
**Research sites**	- Cochin Hospital<br>- Lariboisière Hospital<br>- Clermont-Ferrand University Hospital
**Study duration**	60 months (45-month enrolment period)
**Patient follow-up**	Between 14 and 15 months, depending on the time between the enrolment visit and the intervention for the educational programme associated with an exercise programme
**Study aim**	Primary objective:<br>To assess the effectiveness of a standardised education and exercise programme proposed before a total knee arthroplasty for knee osteoarthritis, in terms of:<br>- ability, at discharge from the surgical department measured at D4, plus or minus 1 day, to carry out lying-sitting and sitting-standing transfers, walk 30 metres and go up and down one flight of stairs;<br>- speed of functional recovery within the first 6 months (area under the curve of the function subscale of the WOMAC index).<br><br>Secondary objectives:<br>- To assess the effectiveness of the intervention in terms of pain, function (personalised WOMAC index), quality of life, number of steps and treatment satisfaction at months 6 and 12;<br>- To estimate and compare the cost of the strategies and, where appropriate, carry out a cost-utility analysis.
**Methodology**	A multi-centre, randomised, controlled study
**Number of sites**	3 sites
**Number of patients**	**300 patients will be enrolled:**<br>150 patients in the experimental arm<br>150 patients in the control arm (usual information and advice given by the surgeon)
**Selection criteria**	Inclusion criteria:<br>- Men or women aged from 50 to 85 years;<br>- Patients with knee osteoarthritis according to the ACR criteria for which a total knee arthroplasty has been scheduled by the surgeon;<br>- Preliminary medical examination, the results of which will be communicated to the patient;<br>- Patients having provided their written informed consent to take part in the study;<br>- Patients affiliated with a social security scheme (beneficiary or entitled party).<br><br>Exclusion criteria:<br>- Institutionalised patients;<br>- Patients having already undergone ipsilateral total knee arthroplasty;<br>- Patients with chronic inflammatory arthritis;<br>- Cognitive or behavioural disorders making the assessment impossible;<br>- Difficulties in understanding and communicating in French;<br>- TKA for an indication other than knee osteoarthritis.
**Early withdrawal criteria**	- Failure to meet the eligibility criteria<br>- The onset during the study of a condition listed in the exclusion criteria

**Study treatment**	4 group sessions consisting of a therapeutic education session and a physical exercise programme
**Study outline**	- Enrolment visit (V0);<br>- Intervention (education programme associated with an exercise programme);<br>- Preoperative visit;<br>- Surgery department discharge visit (medical record information);<br>- Monthly follow-up by post or email in terms of function (and personalised) subscale of the WOMAC index and pain in the past 48 hours, 6 weeks after surgery, 4 and 5 months after surgery, and 6 months after surgery with a self-questionnaire.<br>Postoperative self-questionnaires at months 3 and 12, according to the patient's preference:<br>- Self-questionnaires given at a consultation; or<br>- Self-questionnaires will be sent by post (prepaid envelope); or<br>- Self-questionnaire information can be collected by a clinical study technician by phone.
**Primary and secondary endpoints**	Primary endpoints<br>- Ability at discharge: Assessed at D4, plus or minus 1 day. The endpoints to be assessed will be the ability to carry out lying-sitting and sitting-standing transfers, walk 30 metres and go up and down one flight of stairs.<br>- Speed of postoperative functional recovery at 6 months (area under the curve of the function subscale of the WOMAC index).<br><br>Secondary endpoints<br>- Pain assessment (numeric scale), function (personalised WOMAC index), quality of life (MOS SF-12, EQ-5D), number of steps and treatment satisfaction.<br>- Cost comparison between the two strategies from the Health Insurance perspective and from the care system perspective, as a cost-utility ratio.
**Statistical analysis**	An intention-to-treat analysis will be carried out with the data.<br>The statistical analysis will be carried out at Prof. P. Ravaud's Clinical Ethics Centre at Hôtel Dieu Hospital, under the responsibility of Dr I. Boutron and G. Baron. SAS software will be used.

2 Analysis population

2.1 Patient flow description

A patient flow diagram (flow chart) will be created in line with the CONSORT 2010 recommendations. This will contain:

— The number of eligible, randomised patients and the number of patients having actually followed the intervention assigned by randomisation;
— Early withdrawals from the intervention and the reasons, and patients lost to follow-up;
— The number of patients excluded from the analysis.

The number of patients randomised but ineligible, if any, will also be reported as well as the reason for ineligibility.

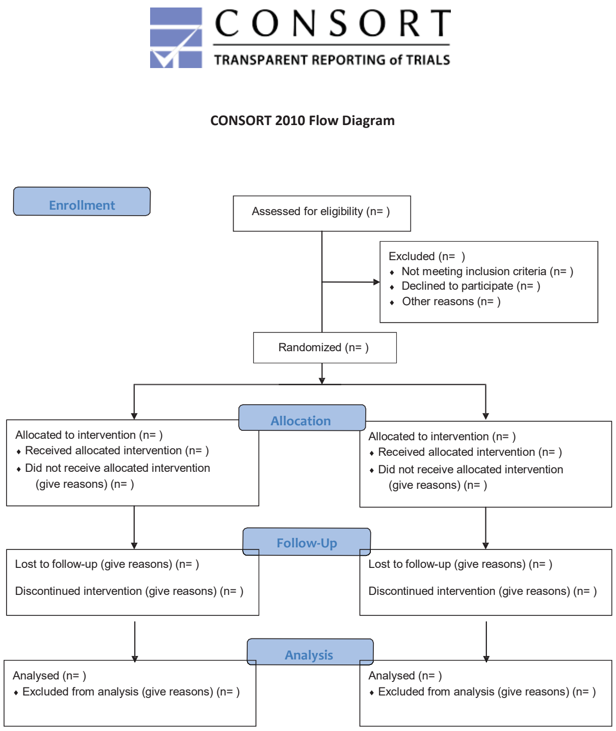

2.2 Analysis populations definition

Intention-to-treat (ITT): this population includes all patients randomised into the trial. The patients are analysed in the group to which they were randomised, regardless of the treatment received and regardless of any protocol deviations.

No data will be analysed for patients for whom a signed informed consent form was not collected or for patients who withdrew their consent to use their data during the course of the study. The data concerning these patients, if any have been collected, will be destroyed. The presence of any such patients will be documented in the study flow chart.

2.3 Calculation of the required number of subjects

The calculation of the required number of subjects is detailed in protocol version no. 5.0 dated 07/04/2017.

3 Principles of analysis

3.1 General principles for the endpoint analysis

The results will be reported in line with the CONSORT 2010 recommendations.

The endpoint analysis will be carried out in the ITT population.

The statistical analysis will be carried out with blinding in terms of the randomisation group.

No interim analysis is planned for this trial.

All the endpoints will be tested for superiority.

As the analyses are based on modelling or other numerical approaches, they require verification of the underlying hypotheses as well as convergence of their algorithms. If these conditions are not verified, an alternative method will be used.

The analyses will be carried out using R software version 3.4.4 or later (The R Foundation for Statistical Computing, Vienna, Austria).

The tests used will be bilateral with a significance threshold of 2.5% for the two primary endpoints and 5% for the secondary endpoints. The 2.5% threshold was chosen to take into account the double primary endpoint.

3.2 Baseline patient characteristics

The baseline patient characteristics collected at enrolment will be described globally and by group in terms of means, standard deviations, medians, interquartile ranges, minimum and maximum for quantitative variables and by their sample sizes and percentages by modality for qualitative variables. The number of missing pieces of data will also be recorded for each variable. No statistical tests comparing the groups will be performed.

3.3 Compliance analysis

Compliance will be described for patients in the intervention group. The number and percentage of patients having taken part in all sessions as well as details of participation per session will be reported. The compliance analysis will be carried out in a second phase, after the primary and secondary endpoint analysis, as it requires unblinding.

3.4 Management of missing data

In the event of missing data regarding the primary endpoints, multivariate imputation by chained equations (MICE) will be carried out.

Imputations will be carried out separately for the patients in the two groups. The baseline characteristics and the primary endpoints (ability at discharge and AUC of the function subscale of the WOMAC index) will be used in the prediction model. Predictive mean matching imputation will be used for the continuous data, logistic regression will be used for the binary data and multinomial regression will be used for the categorical data.

For the MICE procedure, it is common practice to use 10 iterations (Raghunathan TE, Solenberger PW, Van Hoewyk J. IVEware: Imputation and Variance Estimation Software. Ann Arbor, MI: Survey Methodology Program, Survey Research Center, Institute for Social Research, University of Michigan; 2007.

http://www.isr.umich.edu/src/smp/ive/). This value will therefore be used. For the number of imputations, it has been suggested as a general rule to use the same number of imputations as the percentage of observations with missing data (White IR *et al.*, Stat Med, 2010, 30: 377-399). This recommendation will be followed with rounding up to the next unit.

Convergence of the multiple imputation algorithm and the imputation results will be verified (van Buuren S & Groothuis-Oudshoorn K, J Stat Soft, 2011, 45: 1-97)

On the other hand, it has been demonstrated that using multiple imputation does not improve the results in mixed models for longitudinal data and that mixed models on imputed data can give unstable results (Twisk J *et al.*, J Clin Epidemiol, 2013, 66 :1022-1028). The secondary endpoints, analysed using longitudinal mixed models, will therefore not be imputed.

4 Primary endpoint analysis

4.1 Ability at discharge

5.1.1 Definition

The first primary endpoint will be the ability at the time of discharge from the surgical department measured at D4 plus or minus 1 day; if the endpoint is present at D5, the endpoint at D5 will be analysed; if it is present at D4 but not at D5, the endpoint at D4 will be analysed; and if it is only present at D3, the endpoint at D3 will be analysed.

We will assess the 4 tests described by Zavadak KH *et al.*, from D1 to D5 post-surgery:
— transferring from the lying position to the seated position;
— transferring from the seated position to the standing position;
— walking 30 metres;
— going up and down one flight of stairs.

These tests can be performed at 4 levels of independence:
— level 0: the test cannot be performed
— level 1: the test is performed with physical assistance from a third party
— level 2: the test is performed with verbal assistance from a third party
— level 3: the test is performed without any assistance from a third party

These 4 tests for measuring the level of dependence in the early postoperative period are the most widely used [Dowsey 1999, Ganz 2003, Kwoh 1997, Munin 1995, Munin 1998, Walker 2001, Zavadak 1995]. The use of sticks or a walker is allowed at all levels. Only intervention from a physical person, whether verbal or mechanical, is considered to be assistance. A patient will be considered to be independent if he or she performs the 4 tests at level 3. The level of dependency will be assessed every day from D1 to D5 during the patient's hospital stay, by an independent clinical study technician, who is specifically trained and is unaware of the assigned intervention (blinded).

5.1.2 Primary analysis

The proportion of patients who are independent at the time of discharge from the surgical department in each of the arms, the difference between these proportions and their ratio (rate ratio) will be estimated as well as their standard errors. The estimation of the difference in the rate of independence and the rate ratio will be adjusted to the centre. The adjusted analysis of the rate ratio will use a GEE Poisson model with an exchangeable correlation structure assuming a correlation for patients operated on by the same surgeon. The centre will be used as a fixed effect. The adjusted difference in proportions will be derived from the model used for the rate ratio. The analyses will be carried out on each of the imputed databases then pooled according to Rubin's Rules.

5.1.3 Sensitivity analyses

Two sensitivity analyses will be presented: the analysis without adjustment and the analysis without imputation of missing data.

5.2 Speed of functional recovery

5.2.1 Definition

The second primary endpoint is the speed of postoperative functional recovery at month 6. This is assessed using the area under the curve of the function subscale of the WOMAC index. The area under the curve will be defined by the trapezoidal rule.

5.2.2 Primary analysis

The average area under the curve of the function subscale of the WOMAC index will be estimated in each of the groups. These averages will be compared using a linear mixed regression model with the randomisation arm and the centre (adjustment variable) as explanatory variables and the area under the curve of the function subscale of the WOMAC index as the response variable. The analysis will also be adjusted for the surgeon effect (random effect). The adjusted mean difference will be derived from this model. The analyses will be carried out on each of the imputed databases then pooled according to Rubin's Rules.

5.2.3 Sensitivity analyses

Two sensitivity analyses will be presented: the analysis without adjustment and the analysis without imputation of missing data.

6 Secondary endpoint analysis

6.1 Knee pain over the past 48 hours

6.1.1 Definition

Knee pain over the past 48 hours is assessed using a numeric pain scale from 0 to 100 at the enrolment visit, preoperative visit, week 6, month 3, month 4, month 5, month 6 and month 12.

6.1.2 Analysis

The average pain assessment will be estimated in each of the groups. These averages will be compared using a longitudinal analysis. A linear mixed regression model will be used with assessment of pain at each time point (enrolment visit, preoperative visit, week 6, month 3, month 4, month 5, month 6 and month 12) as an explanatory variable with constrained longitudinal data analysis. The randomisation arm, the visit, the arm interaction per visit and the centre (adjustment variable) will be used as fixed effects and the patient (intercept + visit) and the surgeon (intercept + group) will be used as random effects. The adjusted mean differences will be derived from this model. The statistical tests will be performed at months 6 and 12.

6.2 Function

6.2.1 Definition

Function is assessed using the personalised WOMAC scale at the enrolment visit, preoperative visit, week 6, month 3, month 4, month 5, month 6 and month 12.

6.2.2 Analysis

The average personalised WOMAC score will be estimated in each of the groups. These averages will be compared using a longitudinal analysis. A linear mixed regression model will be used with the score at each time point (enrolment visit, preoperative visit, week 6, month 3, month 4, month 5, month 6 and month 12) as an explanatory variable with constrained longitudinal data analysis. The randomisation arm, the visit, the arm interaction per visit and the centre (adjustment variable) will be used as fixed effects and the patient (intercept + visit) and the surgeon (intercept + group) will be used as random effects. The adjusted mean differences will be derived from this model. The statistical tests will be performed at months 6 and 12.

6.3 Quality of life

6.3.1 Definition

Quality of life is assessed using the MOS SF-12 and EQ-5D scales.

For the SF-12, the physical (PCS) and mental (MCS) health scores will be calculated at the enrolment visit, preoperative visit, month 3, month 6 and month 12.

The EQ-5D is collected at the enrolment visit and at months 6 and 12.

6.3.2 Analysis

The average PCS, MCS and EQ5D scores will be estimated in each of the groups. These averages will be compared using longitudinal analyses. Linear mixed regression models will be used with the score at each time point as an explanatory variable with constrained longitudinal data analysis. The randomisation arm, the visit, the arm interaction per visit and the centre (adjustment variable) will be used as fixed effects and the patient (intercept + visit) and the surgeon (intercept + group) will be used as random effects. The adjusted mean differences for each score will be derived from these models. The statistical tests will be performed at months 6 and 12.

6.4 Number of steps

6.4.1 Definition

The number of steps is assessed every day of the week following the enrolment visit and every day of the week before the other visits (preoperative visit, week 6, month 3, month 6 and month 12). The average daily number of steps over the 7 days will be calculated for each visit.

6.4.2 Analysis

The average number of steps will be estimated in each of the groups. These averages will be compared using a longitudinal analysis. A linear mixed regression model will be used with the score at each time point as a response variable with constrained longitudinal data analysis. The randomisation arm, the visit, the arm interaction per visit and the centre (adjustment variable) will be used as fixed effects and the patient (intercept + visit) and the surgeon (intercept + group) will be used as random effects. The adjusted mean differences will be derived from this model. The statistical tests will be performed at months 6 and 12.

6.5 Treatment satisfaction

6.5.1 Definition

Treatment satisfaction is assessed at month 6 and month 12 with a score ranging from 0 to 100.

6.5.2 Analysis

The average treatment satisfaction scores will be estimated in each of the groups. These averages will be compared using a longitudinal analysis. A linear mixed regression model will be used with the score at each time point (month 6 and month 12) as the response variable. The randomisation arm, the visit, the arm interaction per visit and the centre (adjustment variable) will be used as fixed effects and the patient (intercept + visit) and the surgeon (intercept + group) will be used as random effects. The adjusted mean differences will be derived from this model. The statistical tests will be performed at months 6 and 12.

7 Tolerance analysis

7.1 Adverse events

7.1.1 Definition

Any adverse events spontaneously reported by patients at telephone follow-up visits or recorded at a visit will be taken into account. The following information will be collected for each adverse event:

— its intensity (severity): mild, moderate or severe;
— its treatment, if any;
— its duration;
— its causality, as determined by the investigator (not assessable, unlikely, possible, likely, certain, or unrelated to the investigational treatment).

The adverse event rate will be analysed at month 12.

7.1.2 Analysis

Adverse events and their characteristics will be described in the form of sample sizes and percentages by treatment arm. Fisher's exact tests will be used to compare the two treatment arms in terms of the proportion of patients with at least one AE/SAE and the proportion of patients with at least one SAE. The total number of AEs/SAEs and SAEs will also be described for each arm.

Clinical Epidemiology Centre

U1153 - METHODS Team

Statistical analysis plan, version no. 2.0

EDEX:
Evaluation of an Educational Program Associated With Exercises (EDEX) Before Total Knee Arthroplasty

Registration number
NCT01671917

Principal Investigator

Prof. François Rannou
Cochin Hospital, Department of Physical Medicine and Rehabilitation of the Musculoskeletal System and Spinal Disorders, Paris

Drafted by Elodie Perrodeau
Prof. Isabelle Boutron
Prof. Philippe Ravaud
29 March 2018

Table of contents

1 Change log **2**

2 Protocol summary **3**

3 Analysis population **5**
3.1 Patient flow description 5
3.2 Analysis populations definition 5
3.3 Calculation of the required number of subjects 6

4 Principles of analysis **6**
4.1 General principles for the endpoint analysis 6
4.2 Baseline patient characteristics 6
4.3 Compliance analysis 6
4.4 Management of missing data 6

5 Primary endpoint analysis **7**
5.1 Ability at discharge 7
5.1.1 Definition 7
5.1.2 Primary analysis 7
5.1.3 Sensitivity analyses 8
5.2 Speed of functional recovery 8
5.2.1 Definition 8
5.2.2 Primary analysis 8
5.2.3 Sensitivity analyses 8

6 Secondary endpoint analysis **8**
6.1 Knee pain over the past 48 hours 8
6.1.1 Definition 8
6.1.2 Analysis 8
6.2 Function 8
6.2.1 Definition 8
6.2.2 Analysis 9
6.3 Quality of life 9
6.3.1 Definition 9
6.3.2 Analysis 9
6.4 Number of steps 9
6.4.1 Definition 9
6.4.2 Analysis 9
6.5 Treatment satisfaction 9
6.5.1 Definition 9
6.5.2 Analysis 10

7 Tolerance analysis **10**
7.1 Adverse events 10
7.1.1 Definition 10
7.1.2 Analysis 10

8 Health economics analysis **10**

1 Change log

Protocol version	New SAP version	Number of section changed	Description and reasons for the change	Date
5.0	1.1	6.3 and 8	Correction for EQ-5D analysis	26/03/2018
5.0	2.0	No changes	Version validated	29/03/2018

2 Protocol summary

This document was written on the basis of the information contained in protocol version no. 5.0 dated 07/04/2017.

SUMMARY

**Title**	Evaluation of an Educational Program Associated With Exercises (EDEX) Before Total Knee Arthroplasty
**Principal Investigator**	Prof. François Rannou (Cochin Hospital)
**Research sites**	- Cochin Hospital<br>- Lariboisière Hospital<br>- Clermont-Ferrand University Hospital
**Study duration**	60 months (45-month enrolment period)
**Patient follow-up**	Between 14 and 15 months, depending on the time between the enrolment visit and the intervention for the educational programme associated with an exercise programme
**Study aim**	<u>Primary objective</u>:<br>To assess the effectiveness of a standardised education and exercise programme proposed before a total knee arthroplasty for knee osteoarthritis, in terms of:<br>- ability, at discharge from the surgical department measured at D4, plus or minus 1 day, to carry out lying-sitting and sitting-standing transfers, walk 30 metres and go up and down one flight of stairs;<br>- speed of functional recovery within the first 6 months (area under the curve of the function subscale of the WOMAC index).<br><br><u>Secondary objectives:</u><br>- To assess the effectiveness of the intervention in terms of pain, function (personalised WOMAC index), quality of life, number of steps and treatment satisfaction at months 6 and 12;<br>- To estimate and compare the cost of the strategies and, where appropriate, carry out a cost-utility analysis.
**Methodology**	A multi-centre, randomised, controlled study
**Number of sites**	3 sites
**Number of patients**	**300 patients will be enrolled:**<br>150 patients in the experimental arm<br>150 patients in the control arm (usual information and advice given by the surgeon)
**Selection criteria**	<u>Inclusion criteria:</u><br>- Men or women aged from 50 to 85 years;<br>- Patients with knee osteoarthritis according to the ACR criteria for which a total knee arthroplasty has been scheduled by the surgeon;<br>- Preliminary medical examination, the results of which will be communicated to the patient;<br>- Patients having provided their written informed consent to take part in the study;<br>- Patients affiliated with a social security scheme (beneficiary or entitled party).<br><br><u>Exclusion criteria:</u><br>- Institutionalised patients;<br>- Patients having already undergone ipsilateral total knee arthroplasty;<br>- Patients with chronic inflammatory arthritis;<br>- Cognitive or behavioural disorders making the assessment impossible;<br>- Difficulties in understanding and communicating in French;<br>- TKA for an indication other than knee osteoarthritis.
**Early withdrawal criteria**	- Failure to meet the eligibility criteria<br>- The onset during the study of a condition listed in the exclusion criteria

**Study treatment**	4 group sessions consisting of a therapeutic education session and a physical exercise programme
**Study outline**	- Enrolment visit (V0);<br>- Intervention (education programme associated with an exercise programme);<br>- Preoperative visit;<br>- Surgery department discharge visit (medical record information);<br>- Monthly follow-up by post or email in terms of function (and personalised) subscale of the WOMAC index and pain in the past 48 hours, 6 weeks after surgery, 4 and 5 months after surgery, and 6 months after surgery with a self-questionnaire.<br>Postoperative self-questionnaires at months 3 and 12, according to the patient's preference:<br>- Self-questionnaires given at a consultation; or<br>- Self-questionnaires will be sent by post (prepaid envelope); or<br>- Self-questionnaire information can be collected by a clinical study technician by phone.
**Primary and secondary endpoints**	Primary endpoints<br>- Ability at discharge: Assessed at D4, plus or minus 1 day. The endpoints to be assessed will be the ability to carry out lying-sitting and sitting-standing transfers, walk 30 metres and go up and down one flight of stairs.<br>- Speed of postoperative functional recovery at 6 months (area under the curve of the function subscale of the WOMAC index).<br><br>Secondary endpoints<br>- Pain assessment (numeric scale), function (personalised WOMAC index), quality of life (MOS SF-12, EQ-5D), number of steps and treatment satisfaction.<br>- Cost comparison between the two strategies from the Health Insurance perspective and from the care system perspective, as a cost-utility ratio.
**Statistical analysis**	An intention-to-treat analysis will be carried out with the data.<br>The statistical analysis will be carried out at Prof. P. Ravaud's Clinical Ethics Centre at Hôtel Dieu Hospital, under the responsibility of Dr I. Boutron and G. Baron. SAS software will be used.

3 Analysis population

3.1 Patient flow description

A patient flow diagram (flow chart) will be created in line with the CONSORT 2010 recommendations. This will contain:

— The number of eligible, randomised patients and the number of patients having actually followed the intervention assigned by randomisation;
— Early withdrawals from the intervention and the reasons, and patients lost to follow-up;
— The number of patients excluded from the analysis.

The number of patients randomised but ineligible, if any, will also be reported as well as the reason for ineligibility.

CONSORT
TRANSPARENT REPORTING of TRIALS

CONSORT 2010 Flow Diagram

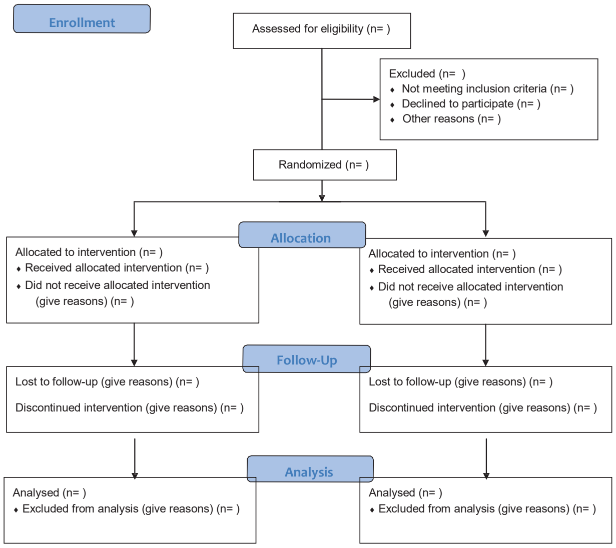

3.2 Analysis populations definition

Intention-to-treat (ITT): this population includes all patients randomised into the trial. The patients are analysed in the group to which they were randomised, regardless of the treatment received and regardless of any protocol deviations.

No data will be analysed for patients for whom a signed informed consent form was not collected or for patients who withdrew their consent to use their data during the course of the study. The data concerning these patients, if any have been collected, will be destroyed. The presence of any such patients will be documented in the study flow chart.

3.3 Calculation of the required number of subjects

The calculation of the required number of subjects is detailed in protocol version no. 5.0 dated 07/04/2017.

4 Principles of analysis

4.1 General principles for the endpoint analysis

The results will be reported in line with the CONSORT 2010 recommendations.

The endpoint analysis will be carried out in the ITT population.

The statistical analysis will be carried out with blinding in terms of the randomisation group.

No interim analysis is planned for this trial.

All the endpoints will be tested for superiority.

As the analyses are based on modelling or other numerical approaches, they require verification of the underlying hypotheses as well as convergence of their algorithms. If these conditions are not verified, an alternative method will be used.

The analyses will be carried out using R software version 3.4.4 or later (The R Foundation for Statistical Computing, Vienna, Austria).

The tests used will be bilateral with a significance threshold of 2.5% for the two primary endpoints and 5% for the secondary endpoints. The 2.5% threshold was chosen to take into account the double primary endpoint.

4.2 Baseline patient characteristics

The baseline patient characteristics collected at enrolment will be described globally and by group in terms of means, standard deviations, medians, interquartile ranges, minimum and maximum for quantitative variables and by their sample sizes and percentages by modality for qualitative variables. The number of missing pieces of data will also be recorded for each variable. No statistical tests comparing the groups will be performed.

4.3 Compliance analysis

Compliance will be described for patients in the intervention group. The number and percentage of patients having taken part in all sessions as well as details of participation per session will be reported. The compliance analysis will be carried out in a second phase, after the primary and secondary endpoint analysis, as it requires unblinding.

4.4 Management of missing data

In the event of missing data regarding the primary endpoints, multivariate imputation by chained equations (MICE) will be carried out.

Imputations will be carried out separately for the patients in the two groups. The baseline characteristics and the primary endpoints (ability at discharge and AUC of the function subscale of the WOMAC index) will be used in the prediction model. Predictive mean matching imputation will be used for the continuous data, logistic regression will be used for the binary data and multinomial regression will be used for the categorical data.

For the MICE procedure, it is common practice to use 10 iterations (Raghunathan TE, Solenberger PW, Van Hoewyk J. IVEware: Imputation and Variance Estimation Software. Ann Arbor, MI: Survey Methodology Program, Survey Research Center, Institute for Social Research, University of Michigan; 2007.

http://www.isr.umich.edu/src/smp/ive/). This value will therefore be used. For the number of imputations, it has been suggested as a general rule to use the same number of imputations as the percentage of observations with missing data (White IR *et al.*, Stat Med, 2010, 30: 377-399). This recommendation will be followed with rounding up to the next unit.

Convergence of the multiple imputation algorithm and the imputation results will be verified (van Buuren S & Groothuis-Oudshoorn K, J Stat Soft, 2011, 45: 1-97)

On the other hand, it has been demonstrated that using multiple imputation does not improve the results in mixed models for longitudinal data and that mixed models on imputed data can give unstable results (Twisk J *et al.*, J Clin Epidemiol, 2013, 66 :1022-1028). The secondary endpoints, analysed using longitudinal mixed models, will therefore not be imputed.

5 Primary endpoint analysis

5.1 Ability at discharge

5.1.1 Definition

The first primary endpoint will be the ability at the time of discharge from the surgical department measured at D4 plus or minus 1 day; if the endpoint is present at D5, the endpoint at D5 will be analysed; if it is present at D4 but not at D5, the endpoint at D4 will be analysed; and if it is only present at D3, the endpoint at D3 will be analysed.

We will assess the 4 tests described by Zavadak KH *et al.*, from D1 to D5 post-surgery:
- transferring from the lying position to the seated position;
- transferring from the seated position to the standing position;
- walking 30 metres;
- going up and down one flight of stairs.

These tests can be performed at 4 levels of independence:
- level 0: the test cannot be performed
- level 1: the test is performed with physical assistance from a third party
- level 2: the test is performed with verbal assistance from a third party
- level 3: the test is performed without any assistance from a third party

These 4 tests for measuring the level of dependence in the early postoperative period are the most widely used [Dowsey 1999, Ganz 2003, Kwoh 1997, Munin 1995, Munin 1998, Walker 2001, Zavadak 1995]. The use of sticks or a walker is allowed at all levels. Only intervention from a physical person, whether verbal or mechanical, is considered to be assistance. A patient will be considered to be independent if he or she performs the 4 tests at level 3. The level of dependency will be assessed every day from D1 to D5 during the patient's hospital stay, by an independent clinical study technician, who is specifically trained and is unaware of the assigned intervention (blinded).

5.1.2 Primary analysis

The proportion of patients who are independent at the time of discharge from the surgical department in each of the arms, the difference between these proportions and their ratio (rate ratio) will be estimated as well as their standard errors. The estimation of the difference in the rate of independence and the rate ratio will be adjusted to the centre. The adjusted analysis of the rate ratio will use a GEE Poisson model with an exchangeable correlation structure assuming a correlation for patients operated on by the same surgeon. The centre will be used as a fixed effect. The adjusted difference in proportions will be derived from the model used for the rate ratio. The analyses will be carried out on each of the imputed databases then pooled according to Rubin's Rules.

5.1.3 Sensitivity analyses

Two sensitivity analyses will be presented: the analysis without adjustment and the analysis without imputation of missing data.

5.2 Speed of functional recovery

5.2.1 Definition

The second primary endpoint is the speed of postoperative functional recovery at month 6. This is assessed using the area under the curve of the function subscale of the WOMAC index. The area under the curve will be defined by the trapezoidal rule.

5.2.2 Primary analysis

The average area under the curve of the function subscale of the WOMAC index will be estimated in each of the groups. These averages will be compared using a linear mixed regression model with the randomisation arm and the centre (adjustment variable) as explanatory variables and the area under the curve of the function subscale of the WOMAC index as the response variable. The analysis will also be adjusted for the surgeon effect (random effect). The adjusted mean difference will be derived from this model. The analyses will be carried out on each of the imputed databases then pooled according to Rubin's Rules.

5.2.3 Sensitivity analyses

Two sensitivity analyses will be presented: the analysis without adjustment and the analysis without imputation of missing data.

6 Secondary endpoint analysis

6.1 Knee pain over the past 48 hours

6.1.1 Definition

Knee pain over the past 48 hours is assessed using a numeric pain scale from 0 to 100 at the enrolment visit, preoperative visit, week 6, month 3, month 4, month 5, month 6 and month 12.

6.1.2 Analysis

The average pain assessment will be estimated in each of the groups. These averages will be compared using a longitudinal analysis. A linear mixed regression model will be used with assessment of pain at each time point (enrolment visit, preoperative visit, week 6, month 3, month 4, month 5, month 6 and month 12) as an explanatory variable with constrained longitudinal data analysis. The randomisation arm, the visit, the arm interaction per visit and the centre (adjustment variable) will be used as fixed effects and the patient (intercept + visit) and the surgeon (intercept + group) will be used as random effects. The adjusted mean differences will be derived from this model. The statistical tests will be performed at months 6 and 12.

6.2 Function

6.2.1 Definition

Function is assessed using the personalised WOMAC scale at the enrolment visit, preoperative visit, week 6, month 3, month 4, month 5, month 6 and month 12.

6.2.2 Analysis

The average personalised WOMAC score will be estimated in each of the groups. These averages will be compared using a longitudinal analysis. A linear mixed regression model will be used with the score at each time point (enrolment visit, preoperative visit, week 6, month 3, month 4, month 5, month 6 and month 12) as an explanatory variable with constrained longitudinal data analysis. The randomisation arm, the visit, the arm interaction per visit and the centre (adjustment variable) will be used as fixed effects and the patient (intercept + visit) and the surgeon (intercept + group) will be used as random effects. The adjusted mean differences will be derived from this model. The statistical tests will be performed at months 6 and 12.

6.3 Quality of life

6.3.1 Definition

Quality of life is assessed using the MOS SF-12 and EQ-5D scales.

For the SF-12, the physical (PCS) and mental (MCS) health scores will be calculated at the enrolment visit, preoperative visit, month 3, month 6 and month 12.

The EQ-5D is collected at the enrolment visit and at months 6 and 12; it will be used for the health economics analysis.

6.3.2 Analysis

The average PCS and MCS scores will be estimated in each of the groups. These averages will be compared using longitudinal analyses. Linear mixed regression models will be used with the score at each time point as an explanatory variable with constrained longitudinal data analysis. The randomisation arm, the visit, the arm interaction per visit and the centre (adjustment variable) will be used as fixed effects and the patient (intercept + visit) and the surgeon (intercept + group) will be used as random effects. The adjusted mean differences for each score will be derived from these models. The statistical tests will be performed at months 6 and 12.

6.4 Number of steps

6.4.1 Definition

The number of steps is assessed every day of the week following the enrolment visit and every day of the week before the other visits (preoperative visit, week 6, month 3, month 6 and month 12). The average daily number of steps over the 7 days will be calculated for each visit.

6.4.2 Analysis

The average number of steps will be estimated in each of the groups. These averages will be compared using a longitudinal analysis. A linear mixed regression model will be used with the score at each time point as a response variable with constrained longitudinal data analysis. The randomisation arm, the visit, the arm interaction per visit and the centre (adjustment variable) will be used as fixed effects and the patient (intercept + visit) and the surgeon (intercept + group) will be used as random effects. The adjusted mean differences will be derived from this model. The statistical tests will be performed at months 6 and 12.

6.5 Treatment satisfaction

6.5.1 Definition

Treatment satisfaction is assessed at month 6 and month 12 with a score ranging from 0 to 100.

6.5.2 Analysis

The average treatment satisfaction scores will be estimated in each of the groups. These averages will be compared using a longitudinal analysis. A linear mixed regression model will be used with the score at each time point (month 6 and month 12) as the response variable. The randomisation arm, the visit, the arm interaction per visit and the centre (adjustment variable) will be used as fixed effects and the patient (intercept + visit) and the surgeon (intercept + group) will be used as random effects. The adjusted mean differences will be derived from this model. The statistical tests will be performed at months 6 and 12.

7 Tolerance analysis

7.1 Adverse events

7.1.1 Definition

Any adverse events spontaneously reported by patients at telephone follow-up visits or recorded at a visit will be taken into account. The following information will be collected for each adverse event:

- its intensity (severity): mild, moderate or severe;
- its treatment, if any;
- its duration;
- its causality, as determined by the investigator (not assessable, unlikely, possible, likely, certain, or unrelated to the investigational treatment).

The adverse event rate will be analysed at month 12.

7.1.2 Analysis

Adverse events and their characteristics will be described in the form of sample sizes and percentages by treatment arm. Fisher's exact tests will be used to compare the two treatment arms in terms of the proportion of patients with at least one AE/SAE and the proportion of patients with at least one SAE. The total number of AEs/SAEs and SAEs will also be described for each arm.

8 Health economics analysis

The EQ-5D scores will be converted into utilities based on French value tables. The utilities imputed for the periods corresponding to the interval between 2 collections will be multiplied by the corresponding durations to obtain QALYs. The differences in QALYs will be compared between the two groups.

The intervention will be valued on a flat-rate basis based on the time spent by the professionals as described in the protocol and by multiplying the estimated cost of each session by the proportion of patients having undergone the sessions.

Hospital admissions related to the pathology (imputed during the end-of-study meeting) will be valued based on 1) ENCC (French national hospital costs study database) costs and 2) rates. The costs will be estimated for each patient and the average costs in each arm will be compared using non-parametric tests.

The incremental cost-utility ratio will be calculated by comparing the difference in average costs with the difference in average QALYs, with a bootstrap sensitivity analysis.
