# Supplementary material for: Effect of Prehabilitation Before Total Knee Replacement for Knee Osteoarthritis on Functional Outcomes: A Randomized Clinical Trial
Source: JAMA Netw Open. 2022 Mar 9;5(3):e221462. doi: 10.1001/jamanetworkopen.2022.1462 (PMC8908069; doi:10.1001/jamanetworkopen.2022.1462)
Supplement: Supplement 2. — eAppendix 1. Substantial Changes to Methods After Trial Commencement and the Reasons for the Changes eAppendix 2. Original Version (French) of the Booklet “Vous allez bénéficier d’une prothèse de genou” eAppendix 3. English Translation of the Slideshow Used for the Educational Program on the Positive Effects of Exercise Therapy Before Total Knee Replacement Conducted by a Physiotherapist or an Instructor in Physical Activity eAppendix 4. English Translation of the Slideshow Used for the Educational Program on Work Rehabilitation and Social Support Conducted by a Social Worker eAppendix 5. English Translation of the Slideshow Used for the Educational Program on Diet and Weight Management Conducted by a Dietician eAppendix 6. English Translation of the Slideshow Used for the Educational Program on the Management of Stress and Anxiety in the Perioperative Period Conducted by a Psychologist eAppendix 7. English Translation of the Slideshow Used for the Educational Program on the Return Home Conducted by an Occupational Therapist eAppendix 8. Supervised Exercise Therapy Sessions eAppendix 9. Non-pharmacological and Pharmacological Co-interventions eAppendix 10. Twelve-Month Costs in the Intervention and Control Groups; Costs Are in Euros [file jamanetwopen-e221462-s002.pdf]

Supplementary Online Content

Nguyen C, Boutron I, Roren A, et al. Effect of prehabilitation before total knee replacement for knee osteoarthritis on functional outcomes: a randomized clinical trial. *JAMA Netw Open*. 2022;5(3):e221462. doi:10.1001/jamanetworkopen.2022.1462

eAppendix 1. Substantial Changes to Methods After Trial Commencement and the Reasons for the Changes

eAppendix 2. Original Version (French) of the Booklet "*Vous allez bénéficier d'une prothèse de genou*"

eAppendix 3. English Translation of the Slideshow Used for the Educational Program on the Positive Effects of Exercise Therapy Before Total Knee Replacement Conducted by a Physiotherapist or an Instructor in Physical Activity

eAppendix 4. English Translation of the Slideshow Used for the Educational Program on Work Rehabilitation and Social Support Conducted by a Social Worker

eAppendix 5. English Translation of the Slideshow Used for the Educational Program on Diet and Weight Management Conducted by a Dietician

eAppendix 6. English Translation of the Slideshow Used for the Educational Program on the Management of Stress and Anxiety in the Perioperative Period Conducted by a Psychologist

eAppendix 7. English Translation of the Slideshow Used for the Educational Program on the Return Home Conducted by an Occupational Therapist

eAppendix 8. Supervised Exercise Therapy Sessions

eAppendix 9. Non-pharmacological and Pharmacological Co-interventions

eAppendix 10. Twelve-Month Costs in the Intervention and Control Groups; Costs Are in Euros

This supplementary material has been provided by the authors to give readers additional information about their work.

© 2022 Nguyen C et al. *JAMA Network Open.*

eAppendix 1. Substantial changes to methods after trial commencement and the reasons for those changes.

**Amendment 1: 06/21/2012**<br>- Modification of the study outline in order to adapt to the procedures of all the participating centers.<br>- Visits at 3 and 12 months postoperatively.<br>- Contact by phone or by mail 6 weeks, 4, 5 and 6 months postoperatively.
**Amendment 2: 04/15/2014**<br>- Update of the reporting circuit for serious adverse events.<br>- Extension of the 12-month inclusion period in order to include the number of patients initially planned and necessary to assess this intervention and its medico-economic value.
**Amendment 3: 05/04/2015**<br>- Request for an extension of the duration of inclusions by 12 months.<br>- Modification of the study plan concerning the modalities of the visits at 3 and 12 months: concerning the follow-up at 3 and 12 months, and taking into account the data that it is planned to collect (self-assessment notebooks), the The choice will be left to the patient to come to the delivery of the self-questionnaires completed during a consultation, or to return the completed self-questionnaires by mail (T envelope) or even to complete the self-questionnaires assisted by a technician clinical studies during a phone call.
**Amendment 4: 04/18/2017**<br>- Modification of the investigator-coordinator, Prof. Francois RANNOU replaces Prof. Serge POIRAUDEAU (deceased)
**Amendment 5: 02/28/2018**<br>- Extension of the total duration of the study by 1 month to be able to follow up on the last patient included.
**Amendment 6: 03/26/2018**<br>- Added precision on the version of the EQ-5D quality of life questionnaire used: this is the EQ-5D-3L version used in the study.<br>- Addition of a clarification concerning the main endpoint (capacity at discharge from the surgical department): the percentage of patients achieving independence on the day of discharge from the orthopedic department will be the main outcome measure. This is a more precise description of the primary outcome.<br>- Addition of details on the secondary objectives and the efficacy and safety criteria: this is a more precise description of the treatment efficacy and safety criteria.

© 2022 Nguyen C et al. *JAMA Network Open.*

eAppendix 2. Original version (French) of the booklet "*le guide de la prothèse*".

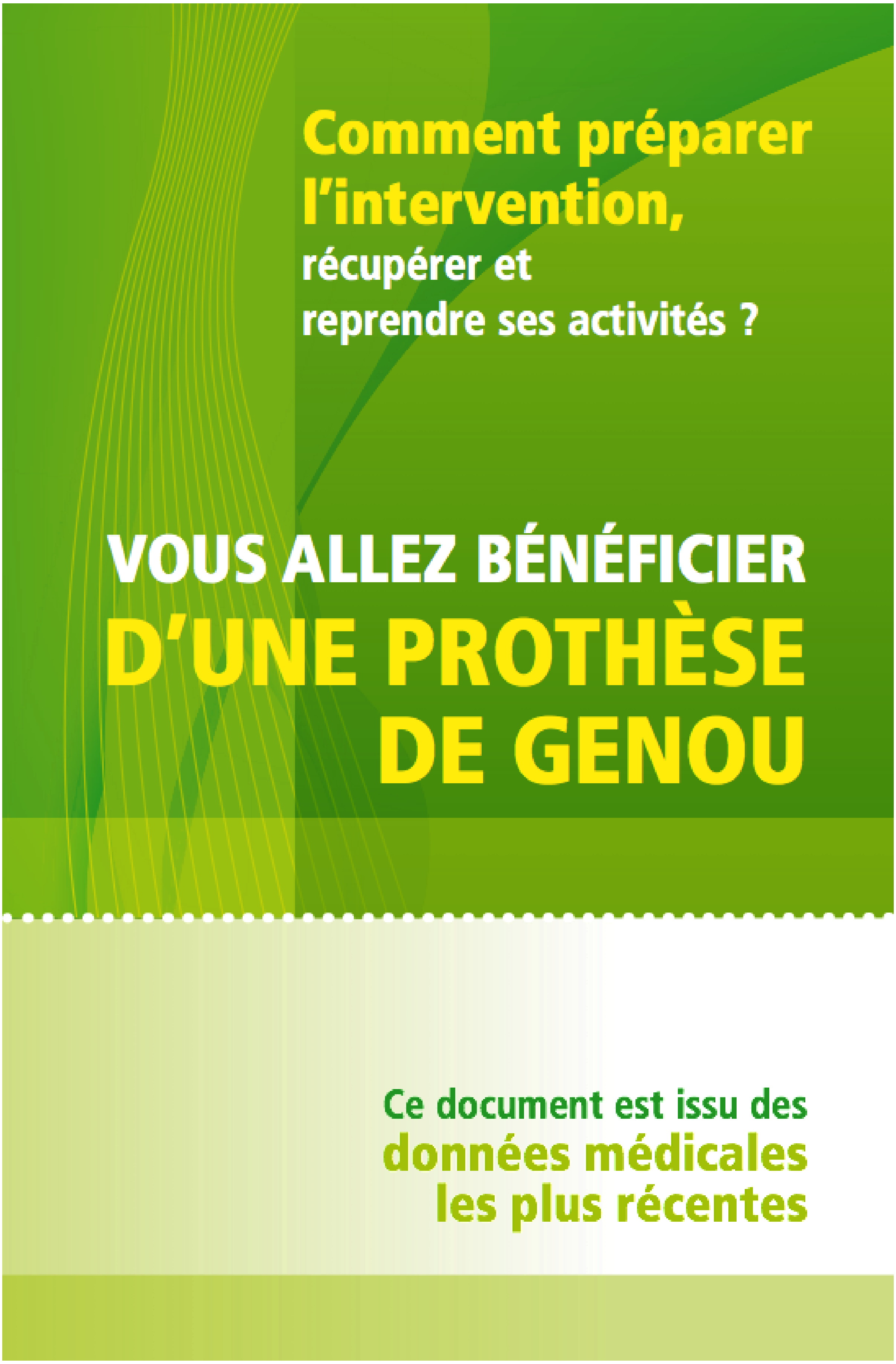

© 2022 Nguyen C et al. *JAMA Network Open.*

Introduction

L'éditeur décline toute responsabilité concernant
les informations contenues dans ce document.
Les conseils donnés par les auteurs
le sont à titre purement informatif
et ne doivent pas être utilisés sans avis médical.

Première édition 2010

Ce document a été établi par les Docteurs
B. Eschalier, D. Claus, S. Descamps, MM. Lefevre-Colau
sous la direction des Professeurs
S. Boisgard et E. Coudeyre.

Imprimerie des Dômes : [illegible]

Vous allez bénéficier d'une prothèse totale de genou,

ce document vous concerne,
Il vous est destiné !

Il a été conçu par une équipe médicale pluridisciplinaire médicale (Médecin de Médecine Physique et de Réadaptation, Médecin Généraliste, Rhumatologue, Chirurgien Orthopédiste) et paramédicale (Masseur-kinésithérapeute, Ergothérapeute, Assistante sociale, Diététicienne, Psychologue et Infirmière) tous acteurs dans la prise en charge de l'arthrose de genou.Ce document a également été approuvé par des patients.

L'ensemble des informations contenues dans ce document est issu d'études scientifiques et a été validé par plusieurs sociétés savantes.

Appropriez-vous ce livret
et devenez l'acteur principal de
la prise en charge de votre problème de santé !

Ce document a dû vous être remis par un médecin qui vous suit régulièrement ; si ce n'est pas le cas, assurez-vous que ce livret vous est bien destiné.

© 2022 Nguyen C et al. *JAMA Network Open.*

L'arthrose c'est quoi ?

L'arthrose est une maladie due à l'usure du cartilage présent au sein de vos articulations. Le cartilage est un tissu vivant qui recouvre les surfaces osseuses de toutes vos articulations.

Il a principalement deux rôles :

- assurer le glissement et donc la mobilité des articulations ;

- amortir les chocs entre les os de l'articulation.

Lorsqu'il n'y a plus suffisamment de cartilage les surfaces osseuses entrent en contact, ce qui est à l'origine de vos douleurs.

L'arthrose est une pathologie chronique mais bénigne qui évolue, dans la grande majorité des cas, lentement (sur plusieurs années) et par poussées.

Toutes les articulations peuvent être atteintes mais certaines le sont plus fréquemment comme le genou et la hanche.

L'objectif du traitement est d'améliorer votre qualité de vie.

Contexte :

l'arthrose touche plus souvent les femmes que les hommes, ainsi que les personnes ayant une surcharge pondérale ou des antécédents traumatiques.

Démarche diagnostique :

le diagnostic est retenu grâce aux symptômes que vous décrivez associés à l'examen clinique (douleur, gonflement, déformation du genou). L'analyse de vos radiographies permet de confirmer ce diagnostic.

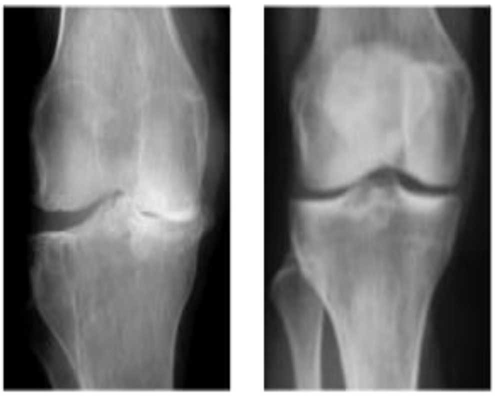

Arthrose évoluée du genou — *Genou normal*

© 2022 Nguyen C et al. *JAMA Network Open.*

Votre genou

Le genou est une articulation supportant l'ensemble du poids du corps ; il est constitué de trois surfaces osseuses :

- l'extrémité inférieure du fémur (os de la cuisse) ;
- l'extrémité supérieure du tibia (un des deux os de la jambe) ;
- la face articulaire de la rotule.

Ces surfaces osseuses, dites articulaires, sont recouvertes de cartilage. De plus, entre le fémur et le tibia s'interposent les ménisques nterne et externe. L'articulation est renforcée par les ligaments latéraux et les ligaments croisés.

Le genou permet essentiellement des mouvements de flexion-extension ;

Cette **mobilité** est assurée par deux principaux groupes musculaires :

- le quadriceps, en avant, qui permet de tendre la jambe ;
- les ischio-jambiers, en arrière, qui permettent de plier la jambe.

Ces deux groupes musculaires jouent également un rôle majeur dans la **stabilité** et le contrôle de votre genou. C'est pour cette raison que l'un des objectifs de la rééducation est de renforcer les muscles.

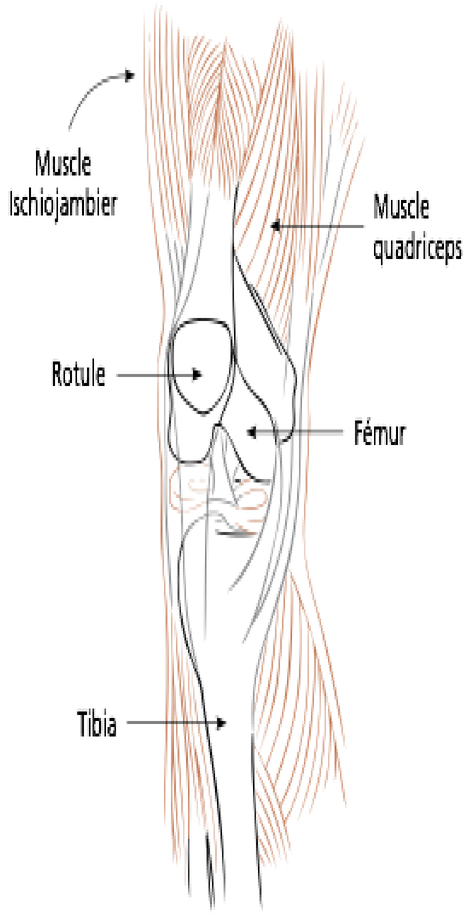

© 2022 Nguyen C et al. *JAMA Network Open.*

Comment traiter votre arthrose du genou ?

La prise en charge de l'arthrose doit associer différents traitements médicamenteux et non médicamenteux. Ces traitements ont fait l'objet de nombreuses études scientifiques et sont validés par la pratique, permettant ainsi de proposer les recommandations suivantes.

La prise en charge associe un traitement de fond et un traitement des poussées.

Traitement de fond :

Il vise à stabiliser la progression de l'usure du cartilage et par conséquent à retarder l'intervention chirurgicale.

Mesures non médicamenteuses :

- **La perte de poids** est essentielle. Elle a pour objectif d'obtenir un Indice de Masse Corporelle (IMC = Poids / Taille^2) inférieur à 25. Cela permet de diminuer la charge supportée par votre genou à chaque pas que vous effectuez. Pour cela vous pouvez vous faire aider par une diététicienne.
- **Le port d'une orthèse de genou** (genouillère) ou d'une orthèse plantaire (semelle) peut vous être préconisé afin d'améliorer la stabilité de votre genou et de décharger les zones douloureuses.
- **Le port d'une contention élastique adhésive** (taping) peut également être proposé, les objectifs étant similaires à celui d'une orthèse.
- **L'utilisation de cannes** (canne simple ou canne béquille) a un intérêt pour limiter les contraintes sur le genou en particulier au cours des périodes douloureuses. Vous devez tenir la canne du côté le moins douloureux.
- **La pratique d'une activité physique régulière** est capitale. Ses bienfaits sont largement démontrés pour l'arthrose comme pour de nombreuses maladies chroniques. Cette activité physique doit associer :
  - **des exercices spécifiques** et adaptés à chacun en fonction du type de déformations et de douleurs que vous présentez. Ils vous seront enseignés par un masseur-kinésithérapeute qui vous montrera des exercices qu'il faudra absolument poursuivre à domicile pour augmenter leur efficacité.
  - **des exercices d'endurance** qui peuvent être réalisés soit **en charge** comme la marche ou **en décharge** tels que le vélo d'appartement, l'aquagym ou la rééducation en piscine si les douleurs sont plus intenses.

Ces exercices peuvent également être réalisés dans le cadre d'une cure thermale.

> *La pratique d'une activité physique régulière contribue à améliorer votre état de santé et n'aggrave pas l'arthrose !*

© 2022 Nguyen C et al. *JAMA Network Open.*

Comment traiter votre arthrose du genou ? (suite)

Il est bien démontré que ces programmes d'exercices ont une efficacité comparable. Associer des exercices spécifiques à un travail d'endurance augmente leur effet.

Mesures médicamenteuses :

- On peut avoir recours à des traitements ayant pour objectif de **protéger le cartilage,** soit sous la forme de comprimés *(traitement anti-arthrosique d'action lente)*, soit sous la forme d'injection dans l'articulation *(acide hyaluronique)* : on parle alors de visco-supplémentation.

Traitement des poussées :

Mesures non médicamenteuses :

Le but est de limiter les contraintes sur votre genou en béquillant à l'aide de canne(s), sans mettre votre articulation au repos complet, ni l'immobiliser.

Vous pouvez également appliquer du froid régulièrement sur votre genou afin de diminuer la douleur et l'œdème. Vous pouvez par exemple utiliser un sac de petit pois congelés.

Mesures médicamenteuses :

- **médicaments contre la douleur** (*dits "antalgiques"*), ces traitements aident à contrôler la douleur, leur puissance sera adaptée à l'intensité de votre douleur; ils doivent être pris régulièrement toutes les 4 à 6 heures.
- **anti-inflammatoires,** ils sont utilisés en deuxième intention, soit en pommade à appliquer localement soit en comprimé.
- Enfin, en cas « d'épanchement » dans l'articulation on peut avoir recours à une ponction avec injection d'anti-inflammatoires à base de cortisone.

Pour un certain nombre de personnes ces divers traitements peuvent être **insuffisants** (variable selon les individus et leur mode de vie). Le temps est alors venu d'envisager la prothèse.

Le but de la chirurgie est de remplacer les structures (os et cartilage) usées par l'arthrose. Dans la majorité des cas, l'intervention permet d'améliorer la qualité de vie, mais il est possible que vous gardiez quelques douleurs, de temps à autre. Ces douleurs ne doivent pas entraver la reprise de vos activités personnelles ou professionnelles.

Bien traiter vos douleurs permet de garder une activité physique régulière !

© 2022 Nguyen C et al. *JAMA Network Open.*

Que pouvez-vous faire avant l'opération ?

Dès la chirurgie envisagée, vous devez vous préparer à l'intervention. En effet, des études scientifiques ont montré qu'une bonne condition physique pré-opératoire permet d'améliorer la récupération post-opératoire et de permettre un retour à domicile plus précoce. On sait par ailleurs qu'**un séjour en rééducation n'est pas une obligation après l'intervention chirurgicale.**

Rééducation et programmes d'exercices physiques :

Les exercices doivent être débutés sous la conduite d'un masseur-kinésithérapeute, seul(e) ou en groupe, et surtout poursuivis à domicile. Le **type d'exercices physiques** ainsi que **sa fréquence** et **son intensité** vous seront adaptés. Vous serez suivi(e) régulièrement par votre masseur-kinésithérapeute.

Il vous apprendre également à **béquiller**, sur **terrain plat** et dans les **escaliers** (montée et descente), ainsi qu'à réaliser vos **transferts** (lit-fauteuil et inversement).

L'apprentissage de ces exercices avant l'intervention vous sera d'une grande aide en post-opératoire et facilitera votre récupération.

l'objectif est d'arriver à l'intervention dans les meilleures conditions.

Ergothérapie :

Un avis auprès d'un ergothérapeute vous sera éventuellement proposé afin de vous préparer, ainsi que votre entourage, au retour à domicile. Ce type d'avis est réalisable en milieu hospitalier (hôpital ou centre de rééducation) mais souvent peu accessible en pratique libérale.

Si besoin, des aménagements de votre lieu de vie peuvent être discutés. Par exemple, faire poser une barre d'appui dans les sanitaires ou utiliser un réhausseur sur les toilettes, mettre un siège dans la douche ou une planche de transfert dans la baignoire. Si votre lit est un peu bas, vous pouvez également en réhausser les pieds. Il est préférable d'enlever provisoirement les tapis de votre domicile pour ne pas risquer de chuter.

Des aides techniques comme un chausse-pied à long manche ou un enfile bas peuvent également vous être utiles.

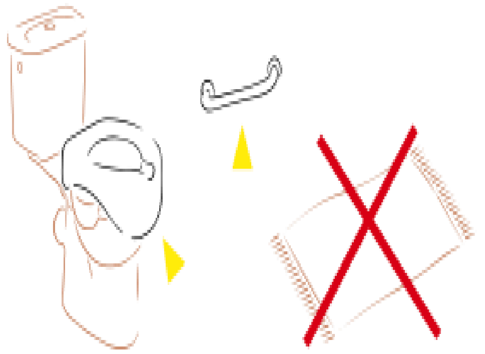

© 2022 Nguyen C et al. *JAMA Network Open.*

Que pouvez-vous faire avant l'opération ? (suite)

Cette période pré-opératoire est propice à la préparation de l'orientation après l'intervention chirurgicale : vous pouvez rentrer directement chez vous après l'intervention et réaliser votre rééducation en libéral ou être pris(e) en charge dans un service de rééducation.

Pour faciliter cette décision, on dispose actuellement de critères consensuels permettant une aide à l'orientation. Ces différents critères permettent d'évaluer vos capacités à retourner directement à domicile après la chirurgie.

Ces paramètres concernent :

- votre âge et votre sexe ;
- votre périmètre de marche, c'est-à-dire la plus longue distance que vous pouvez parcourir sans difficulté avant la chirurgie, avec ou sans canne(s) ;
- l'utilisation d'aides techniques pour la marche avant l'intervention (canne simple, canne anglaise, déambulateur) ;
- la présence ou la possibilité d'avoir des aides à domicile (infirmière, aide ménagère, portage des repas) ;
- votre mode de vie, votre habitat, votre isolement géographique.

La décision définitive sera prise après l'opération par l'équipe chirurgicale, en tenant compte de ces différents critères, des suites opératoires et de votre préférence.

Bas de contention :

La chirurgie est une période propice aux phlébites. Il s'agit de la formation de caillots de sang dans les veines de vos jambes consécutifs à un alitement prolongé et à la chirurgie. La migration de ces caillots dans les poumons peut être responsable d'une embolie pulmonaire.

Pour limiter ces risques le port de bas de contention est souhaitable. Il est important de les porter tous les jours dans le mois suivant l'intervention. Par ailleurs, un anticoagulant préventif vous sera systématiquement prescrit, sous forme d'injection ou de comprimé.

Préparez dès aujourd'hui votre retour à domicile !

© 2022 Nguyen C et al. *JAMA Network Open.*

Votre intervention chirurgicale

L'objectif de cette chirurgie du genou est de traiter une articulation usée par l'arthrose, en remplaçant les surfaces articulaires les plus atteintes (extrémité inférieure du fémur, extrémité supérieure du tibia, face articulaire de la rotule). **Il s'agit d'une intervention très courante puisque 50000 prothèses totales de genou sont posées chaque année en France.**

Indication :

Cette prothèse de genou vous sera proposée lorsque votre arthrose sera devenue trop douloureuse et invalidante et que les différents traitements auront atteints leurs limites. Dans tous les cas, il s'agit d'une intervention qui n'a pas de caractère urgent. Elle vise à améliorer votre confort et le fonctionnement de votre genou.

Bilan à réaliser avant l'intervention :

Vous verrez un anesthésiste dans les semaines précédents l'intervention. Au cours de cette consultation, il vous prescrira un bilan sanguin éventuellement associé à un bilan cardiologique selon vos antécédents. Certains de vos médicaments devront être interrompus quelques jours avant l'opération. C'est le cas, par exemple, des anticoagulants ou de l'aspirine.

De plus, avant la mise en place de la prothèse, il est impératif de vérifier que vous n'avez pas de problèmes infectieux pour limiter les risques d'infection au moment de la chirurgie. Vous devez donc réaliser une analyse d'urine une semaine avant l'intervention. De même, vous devez voir votre dentiste afin de vérifier l'absence de caries ou d'abcès dentaires. Vérifiez également l'absence de mycose aux pieds.

L'hospitalisation :

Vous serez admis dans le service de chirurgie orthopédique au plus tôt la veille de votre intervention. Pensez à amener l'ensemble des résultats des examens prescrits, votre carte de groupe sanguin, vos cannes béquilles et vos bas de contention.

La veille au soir et le matin de l'intervention, vous prendrez une douche avec un désinfectant.

L'hospitalisation en chirurgie durera environ quelques jours au cours desquels vous débuterez la rééducation. Les drains (redons) seront enlevés le deuxième ou le troisième jour et le pansement sera refait régulièrement par les infirmières.

Enfin, les fils ou les agrafes seront enlevés entre le quinzième et le vingt et unième jour post-opératoire par une infirmière.

© 2022 Nguyen C et al. *JAMA Network Open.*

Votre intervention chirurgicale (suite)

Modalités :

L'intervention se déroule au bloc opératoire, sous anesthésie générale ou loco-régionale (seule la partie inférieure du corps est anesthésiée), et dure une à deux heures. La fixation est solide et permet la mobilisation **immédiate** du genou et l'appui du côté opéré.

L'incision se fait au milieu du genou et la cicatrice mesure environ 10 à 20 cm de long.

Complications :

Elles sont rares et vous seront expliquées par votre chirurgien. Toutes les mesures possibles sont prises par le chirurgien et l'anesthésiste pour les éviter.

Un hématome du genou dans les jours suivant l'intervention est le plus souvent banal.

Il est important que vous connaissiez les signes qui doivent vous amenez à consulter rapidement :

- une douleur du mollet, une chaleur, un gonflement ou une sensation de fermeté d'un mollet, un essoufflement inhabituel ou une douleur dans la poitrine ;
- une douleur intense à l'appui et qui persiste ;
- un genou rouge, chaud, œdématié ou de la fièvre ;
- une douleur persistante et invalidante ;
- une récupération insuffisante se traduisant par des difficultés pour plier et tendre la jambe ;
- des douleurs en regard de la cicatrice ;
- une fatigue ou une pâleur liée à une anémie qui pourra nécessiter une supplémentation en fer voire une transfusion sanguine ;

En présence d'un de ces signes, consultez votre médecin traitant qui le cas échéant pourra vous orienter vers l'équipe chirurgicale qui vous a pris(e) en charge.

Rééducation post-opératoire :

Vous pourrez vous lever le jour même ou le lendemain de l'intervention. Le premier lever se fera sous surveillance à l'aide de vos deux cannes béquilles. Vous pourrez faire au minimum quelques pas en fonction de votre fatigue secondaire à l'intervention et de vos douleurs (sauf recommandations particulières).

Dès le lendemain de l'intervention, un masseur-kinésithérapeute vous fera travailler la flexion et l'extension du genou. Un appareillage motorisé (arthro-moteur) est parfois utilisé pour réaliser des mouvements de flexion-extension du genou.

© 2022 Nguyen C et al. *JAMA Network Open.*

Votre intervention chirurgicale (suite)

Vous ferez également des exercices, à poursuivre vous même, comme par exemple :

- écraser un coussin jambe tendue pour prévenir la fonte musculaire de la cuisse et améliorer la stabilité de votre genou. Le coussin doit être enlevé après l'exercice ;

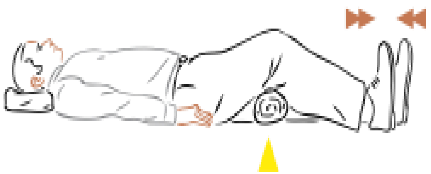

- des mouvements de flexion-extension de la cheville associés à un premier lever précoce afin de prévenir les risques de phlébite. Ce risque sera également prévenu par la prise quotidienne d'anti-coagulants (en injection ou en comprimé) à poursuivre pendant plusieurs semaines, associé au port de bas de contention.

Il faut éviter de laisser votre genou fléchi trop longtemps. En cas de douleurs, l'équipe soignante (médecins et infirmières) pourra adapter votre traitement.

Assis au fauteuil, il faut essayer de garder votre genou tendu tout en évitant le port-à-faux.

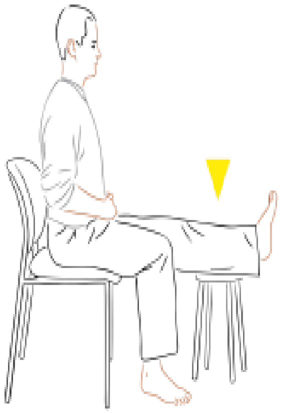

Des exercices de kinésithérapie respiratoire vous seront prodigués en cas d'encombrement bronchique.

Vous serez normalement capable de marcher seul(e) avec appui et avec une ou deux cannes béquilles une semaine environ après l'intervention.

Suivi orthopédique :

Vous reverrez votre chirurgien dans les semaines suivant l'intervention, plus tôt si nécessaire, puis environ une fois par an, avec une radiographie de contrôle.

© 2022 Nguyen C et al. *JAMA Network Open.*

Votre rééducation après l'intervention chirurgicale

Où effectuer votre rééducation ?

Comme nous l'avons vu précédemment, ce point devra être abordé avant l'intervention et sera réévalué une fois opéré(e) avec l'équipe chirurgicale. Plusieurs orientations sont possibles :

- **un retour à domicile** avec rééducation ambulatoire réalisée par un masseur-kinésithérapeute libéral idéalement au cabinet mais possiblement à domicile puis au cabinet.
- Dans certains cas une prise en charge en **hôpital de jour** dans un service de rééducation peut être proposée ;
- **une hospitalisation** en service de rééducation pour une courte durée afin d'acquérir une autonomie compatible avec votre retour à domicile.

L'orientation n'est pas liée uniquement à l'intervention ou à la rééducation mais prend en compte :

- votre état de santé avant et après intervention,
- votre âge et votre sexe,
- la présence d'aides humaines (infirmière, aide ménagère, portage des repas) et techniques (aménagement du domicile, cannes anglaises) à votre domicile,
- la présence d'une personne vivant avec vous à domicile et susceptible de prendre soin de vous à votre retour,
- vos capacités fonctionnelles (périmètre de marche, escaliers, transferts),
- l'accessibilité de votre domicile.

Quels en sont les objectifs ?

L'objectif initial est de **soulager la douleur** par différents moyens : massage, alternance chaud-froid, drainage, glaçage... L'ensemble de ces techniques que vous effectuerez avec votre masseur-kinésithérapeute permettent de lutter contre l'œdème de votre genou.

Après diminution de la douleur, vous allez récupérer progressivement la **souplesse** de votre articulation en travaillant la flexion et l'extension. L'étirement des muscles de la cuisse (quadriceps et ischio-jambiers) permet également d'assouplir votre genou.

Votre objectif final est de retrouver la **mobilité de l'articulation** compatible avec les activités de la vie quotidienne (il faut environ 90° de flexion pour la marche à plat et plus de 110° pour monter et descendre les escaliers sans gêne).

© 2022 Nguyen C et al. *JAMA Network Open.*

Il faut veiller à respecter votre douleur dans les premiers temps. Il ne faut pas forcer trop vite au risque d'augmenter vos douleurs et de retarder votre récupération. **Prenez votre temps, il n'y a pas d'urgence!** Vous avez attendu l'intervention, attendez également votre récupération complète avant de forcer et de reprendre des activités physiques intensives.

Un traitement antalgique, voire anti-inflammatoire, vous sera prescrit en post-opératoire. Il doit être évalué et éventuellement modifié au fil de la prise en charge.

Enfin, des exercices vous seront proposés afin de récupérer la **force musculaire** de votre cuisse. Un **reconditionnement à l'effort** sera associé pour retrouver une bonne condition physique.

Un travail de **stabilisation** du genou et de l'**équilibre** sera également réalisé.

Une rééducation en piscine (balnéothérapie) peut être proposée. Elle n'est pas obligatoire et n'est possible que lorsqu'une une cicatrisation complète est obtenue.

Combien de temps va durer votre rééducation ?

La durée des programmes de rééducation est variable. Elle dépend de l'état de votre genou avant l'intervention, de votre état général, de vos capacités de récupération, de la survenue éventuelle de complications et bien sûr de votre investissement !

Comment allez-vous être suivi(e) ?

Si la récupération de mobilité est insuffisante, votre médecin traitant pourra vous adresser en consultation auprès du chirurgien qui vous a opéré(e) voire auprès d'un médecin de Médecine Physique et de Réadaptation qui pourrons vous proposer soit :

- de poursuivre la prise en charge à domicile avec des consultations régulières de suivi ;
- une hospitalisation en service de rééducation.

De façon exceptionnelle, lorsque la récupération de mobilité n'est pas satisfaisante, une mobilisation de votre genou sous anesthésie générale peut être proposée.

Un séjour en centre de rééducation n'est pas obligatoire pour obtenir un bon résultat !

© 2022 Nguyen C et al. *JAMA Network Open.*

Votre retour à domicile

Préparez votre retour à domicile dès l'intervention envisagée. Après l'intervention vous serez centré(e) sur votre récupération et peut être encore fatigué(e), ce qui est normal ! Pour cela, renseignez-vous auprès de votre mairie ou au Centre Communal d'Action Sociale de votre domicile. Vous pouvez éventuellement solliciter votre assistante sociale de secteur qui coordonnera l'ensemble du projet. Elle peut vous aider à trouver des financements.

Renseignez-vous également auprès de votre mutuelle et de votre assureur (assurance habitation) car certains contrats allouent des aides temporaires dans les suites d'une intervention chirurgicale.

La majorité d'entre vous pourra retourner à domicile dès la sortie du service de chirurgie orthopédique. Afin que vous vous sentiez en sécurité et en confiance un certain nombre de mesures peuvent être prises :

- aménagement temporaire de votre domicile : barre et tapis antidérapant dans la baignoire, réhausseur de toilettes, chambre, sanitaires et cuisine au même étage...
- utilisation d'aides techniques : cannes béquilles, enfile chaussettes...
- aides humaines : présence provisoire d'un membre de votre famille à domicile, aide ménagère, infirmière et masseur-kinésithérapeute à domicile, portage des repas...

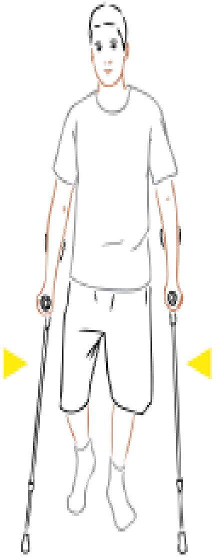

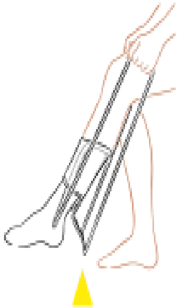

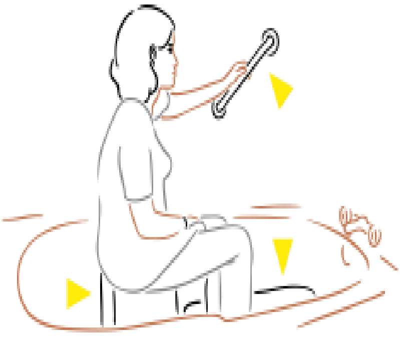

© 2022 Nguyen C et al. *JAMA Network Open.*

La reprise de vos activités

La reprise de vos activités quotidiennes :

La plupart des personnes opérées d'une prothèse de genou peuvent marcher environ un mois et demi après l'intervention, sur plus d'un kilomètre sans canne ni douleur. En revanche quelques personnes gardent une canne un peu plus longtemps en raison d'une faiblesse musculaire ou d'un instabilité sur terrain plat. Ce n'est pas grave, c'est simplement une question de temps. Si vous êtes dans ce cas, poursuivez vos efforts de renforcement musculaire avec votre masseur-kinésithérapeute !

De plus, vous pourrez reprendre rapidement votre voiture, en tant que passager dès la deuxième semaine puis au volant dès le deuxième mois. Certains d'entre vous se sentiront prêts avant, ne précipitez pas les choses. Il faut que votre genou soit assez souple pour conduire et surtout que vos muscles soient forts pour appuyer sur les pédales. A l'inverse, si vous manquez d'assurance alors que votre chirurgien ou votre médecin vous a donné le feu vert, débutez progressivement sur de petits trajets que vous connaissez bien, en vous faisant accompagner.

Il vous faudra environ 6 mois pour récupérer la quasi totalité de la fonction de votre genou.

La reprise de vos activités professionnelles :

Votre aptitude à la reprise de vos activités professionnelles sera évaluée par votre chirurgien orthopédiste, en collaboration avec un médecin de Médecine Physique et de Réadaptation et votre médecin traitant. Vous devez contacter votre médecin du travail avant votre reprise afin de discuter d'un éventuel aménagement de votre poste de travail. Dans certains cas, la reprise à mi-temps thérapeutique peut être discutée.

Vous pourrez reprendre votre activité professionnelle dès que vous vous en sentirez capable. En moyenne, la reprise se fait au troisième ou quatrième mois ; ce délai est évidemment fonction du type de profession que vous exercez.

La reprise de vos activités sportives :

Il est communément recommandé d'attendre entre trois et six mois avant la reprise de tout sport de façon intensive. En effet la pratique d'une activité physique augmente le risque de descellement et d'usure prématurée de la prothèse, et ce d'autant plus qu'il s'agit d'une activité pratiquée de façon intensive.

© 2022 Nguyen C et al. *JAMA Network Open.*

La reprise de vos activités

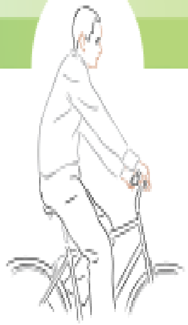

Si vous connaissiez et pratiquiez un sport avant la chirurgie, il vous sera d'autant plus facile de le reprendre.

On peut classer les sports en trois catégories selon leur nocivité pour une prothèse de genou :

- **activités recommandées :** activité d'endurance (marche, vélo d'appartement, natation), aquagym, danse, golf, tir.
- **activités recommandées avec une expérience préalable** : vélo sur route, randonnée, ski de fond, tennis en double, musculation, canoë, aviron.
- **activités non recommandées** (voire contre-indiquées pour les sports en "pivot"): football, basket-ball, handball, volley-ball, gymnastique, jogging, squash, escalade, tennis en simple, ski alpin

Si vous souhaitez reprendre un sport après la chirurgie, il est important de le signaler au chirurgien pour adapter le choix du matériel et le type de fixation.

De plus, il est recommandé de pratiquer des activités régulières en endurance, comme la marche, la natation et le vélo d'appartement, idéalement au moins une demi-heure par jour.

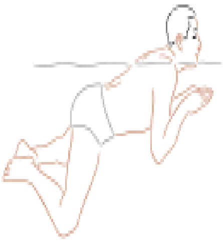

VOS ATTENTES :

Un certain nombre d'études scientifiques ont été réalisées afin de mieux comprendre les attentes des patients candidats à une prothèse totale de genou. Il en ressort principalement trois types d'attentes : une amélioration de la douleur, une amélioration des capacités fonctionnelles conduisant à une amélioration de la qualité de vie des patients et un retour au mode de vie antérieur.

Certains patients sont insatisfaits de l'opération lorsque le résultat ne répond pas totalement à ce qu'ils en attendent. Pour cela il est important de discuter avec votre médecin, avant l'opération, sur les bénéfices attendus de la prothèse.

Il faut rester réaliste sur les bénéfices apportés par la prothèse

© 2022 Nguyen C et al. *JAMA Network Open.*

Vous allez bénéficier d'une prothèse de genou est un livret destiné aux patients candidats à une prothèse totale de genou.

Ce document est issu des données scientifiques les plus récentes, validées par une équipe pluri-disciplinaire, afin de permettre aux patients de se préparer au mieux à l'intervention et donc de récupérer et de reprendre leurs activités au plus vite et dans de meilleurs conditions.

Ce document a été écrit par une équipe de spécialistes :

Chirurgie Orthopédique :

Pr Stéphane BOISGARD, Clermont-Ferrand.
Dr Stéphane DESCAMPS, Clermont-Ferrand.

Médecine Physique et de Réadaptation :

Pr Emmanuel COUDEYRE, Clermont-Ferrand.
Dr Marie-Martine LEFEVRE-COLAU, Paris.

Rhumatologie :

Dr Delphine CLAUS, Clermont-Ferrand.

Médecine Générale :

Dr Bénédicte ESCHALIER, Clermont-Ferrand.

© 2022 Nguyen C et al. *JAMA Network Open.*

eAppendix 3. English translation of the slideshow used for the educational program on the positive effects of exercise therapy before total knee replacement conducted by a physiotherapist or an instructor in physical activity.

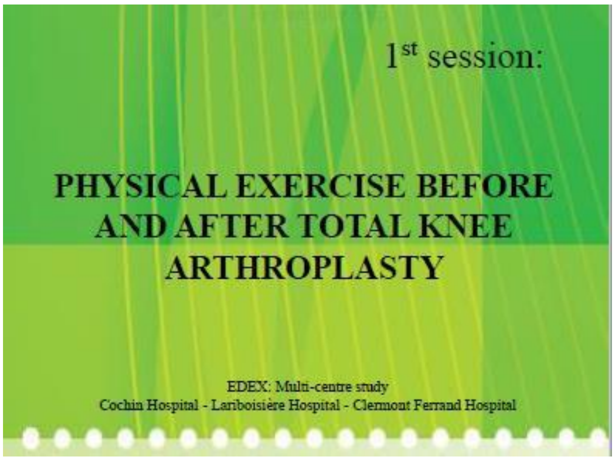

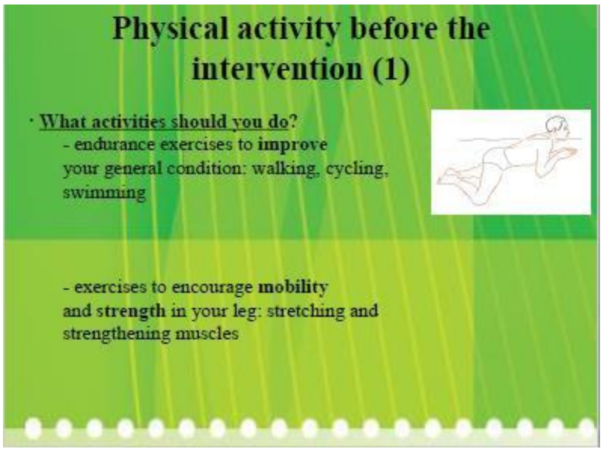

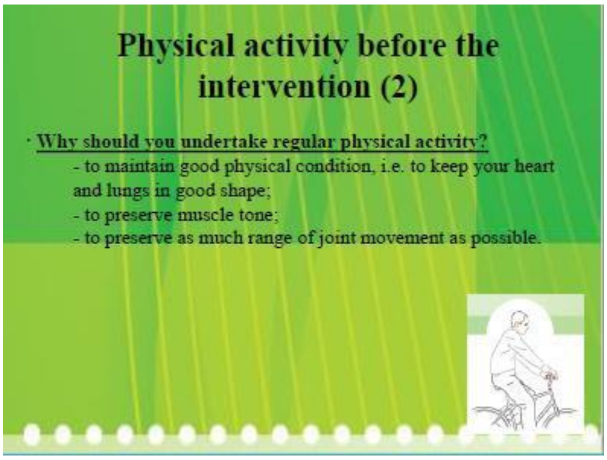

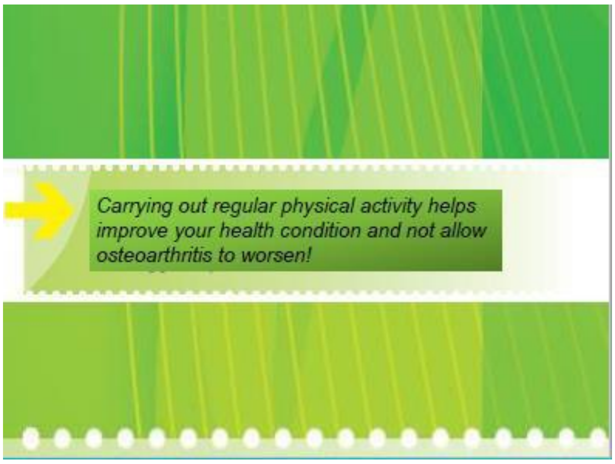

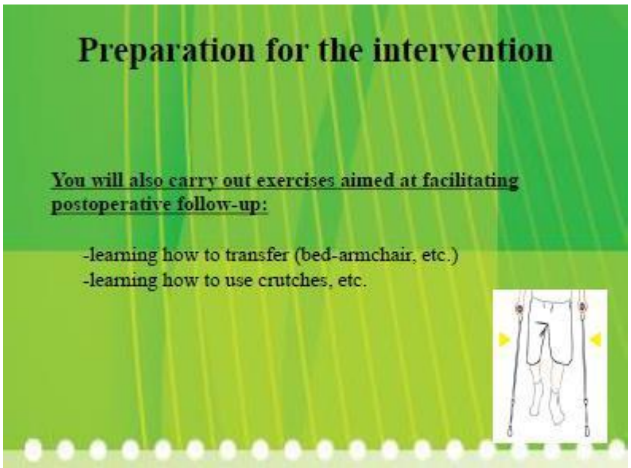

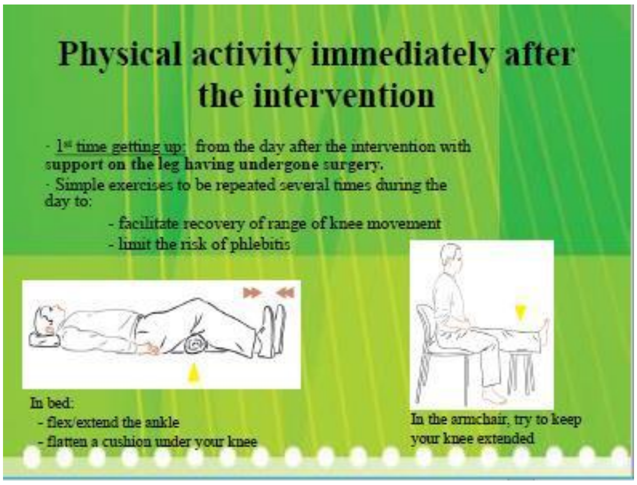

© 2022 Nguyen C et al. *JAMA Network Open.*

Rehabilitation after admission to the surgical department (1)

· **What exercises can you do? For what purpose?**

- to relieve the **pain**: massages, drainage, ice
- to regain **mobility** and **flexibility**: knee flexion-extension, stretching of the thigh muscles
- to recover **muscle strength**: contraction of thigh muscles
- working on **balance**

Rehabilitation in a swimming pool is not mandatory!!
Furthermore, this is only possible once the healing is complete.

Rehabilitation after admission to the surgical department (2)

· **Where is it done?**

- at **home** by following an exercise programme;
- at your **physiotherapist's practice**;
- at a rehabilitation department at the **day hospital**;
- at a rehabilitation department during **traditional hospital admission**.

Staying at a rehabilitation centre is not mandatory to achieve a good outcome!

Resuming your daily activities

- This must be done **gradually**:
  - being a car passenger → 2 weeks after the intervention
  - walking 1 km pain-free without a stick → 1 ½ months after the intervention
  - driving your car → 2 months after the intervention
  - resuming your professional activities → 3 to 4 months after the intervention (discuss with your occupational physician)
  - resuming your sporting activities → 3 to 6 months after the intervention

Resuming your sporting activities

- If you did sports before the intervention, it will be easier to resume this

Recommended activities:
walking, cycling, swimming, water aerobics, dancing, golf.

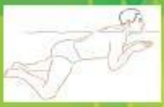

Activities recommended if you have previous experience:
road cycling, hiking, cross-country skiing, doubles tennis, weight training, canoeing, rowing.

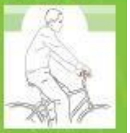

Activities not recommended
pivot sports, football, basketball, handball, volleyball, jogging, squash, singles tennis, rock-climbing, downhill skiing.

No movement is to be avoided after undergoing total knee arthroplasty

You will need around 6 months to recover almost full function of your knee.

Conclusions

- **Physical exercise before a TKA is essential to facilitate post-intervention recovery**
- **You can lean on your knee as of the day after your intervention**
- **You can resume your daily and sporting activities gradually**

© 2022 Nguyen C et al. *JAMA Network Open.*

eAppendix 4. English translation of the slideshow used for the educational program on work rehabilitation and social support conducted by a social worker.

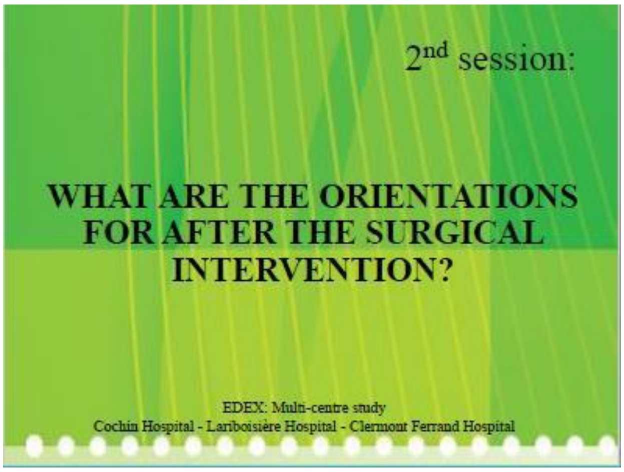

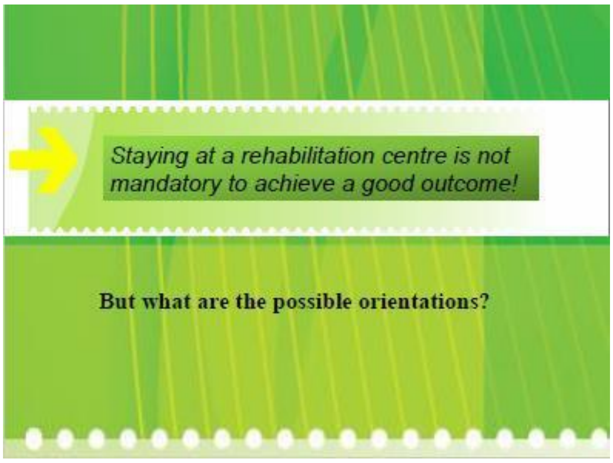

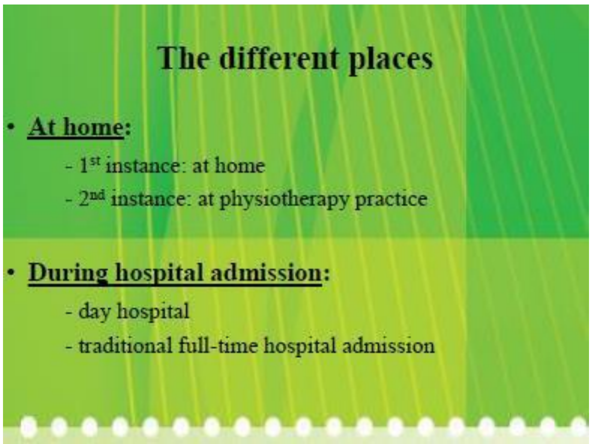

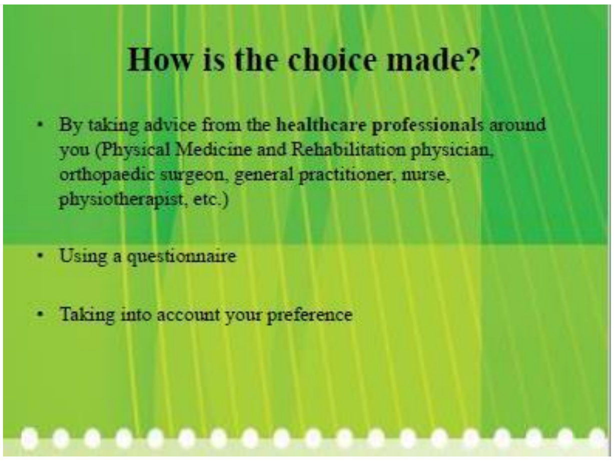

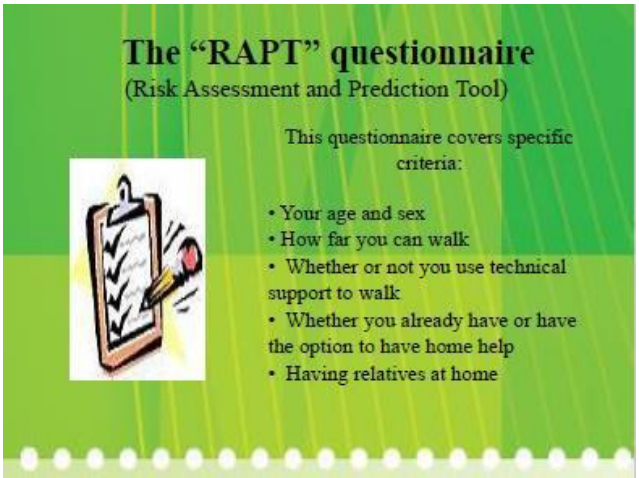

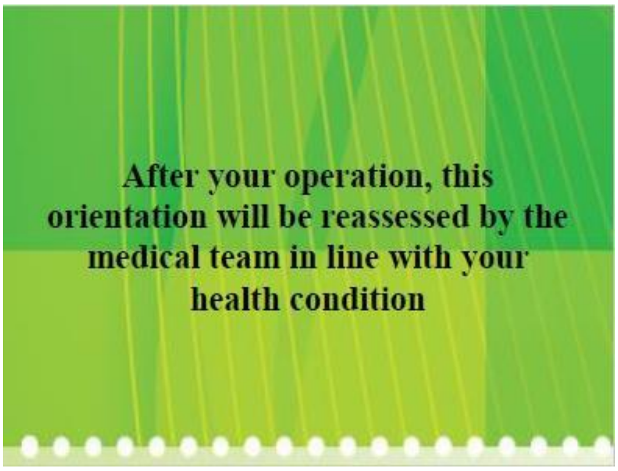

© 2022 Nguyen C et al. *JAMA Network Open.*

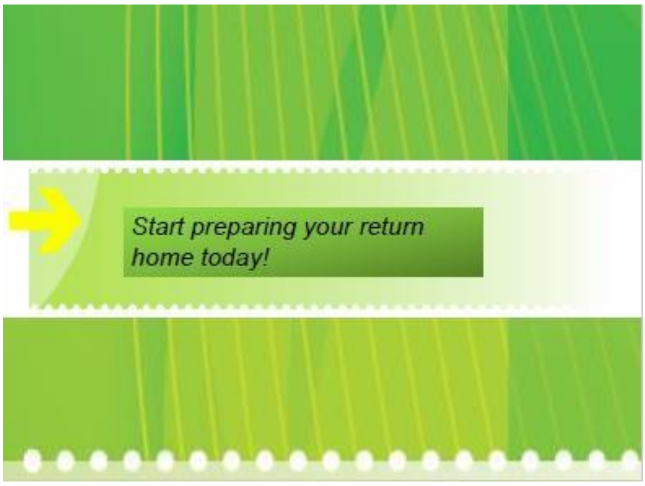

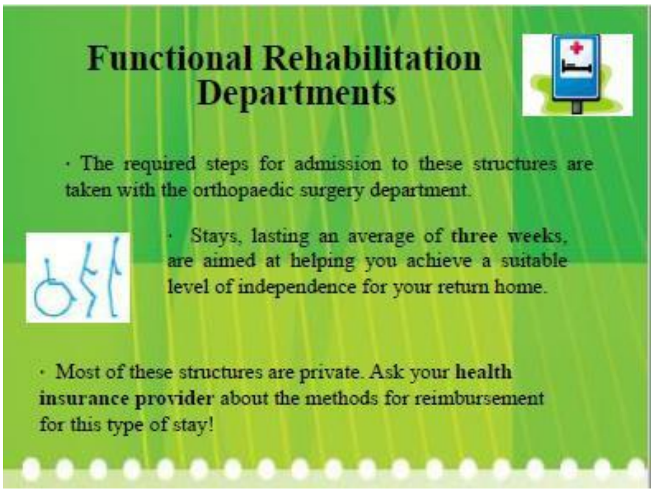

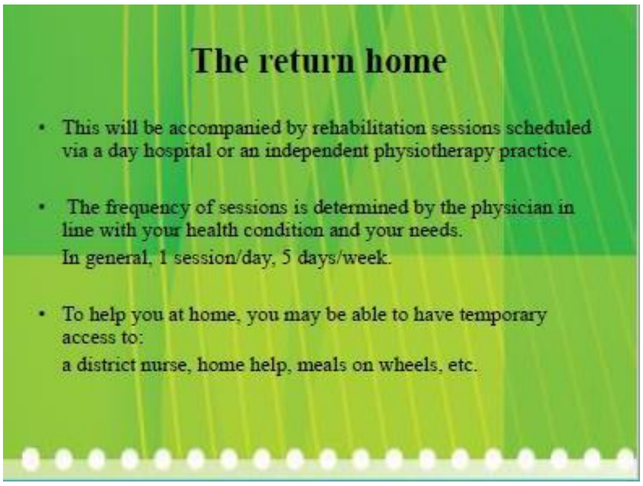

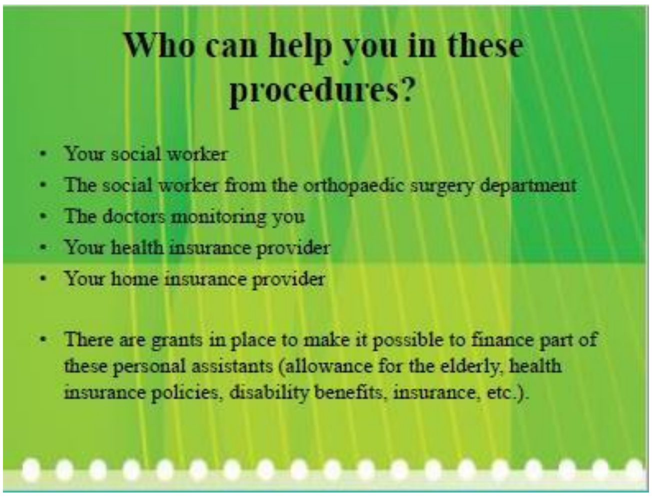

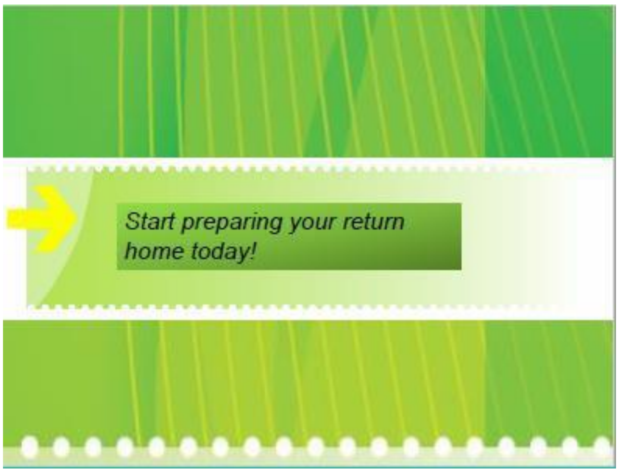

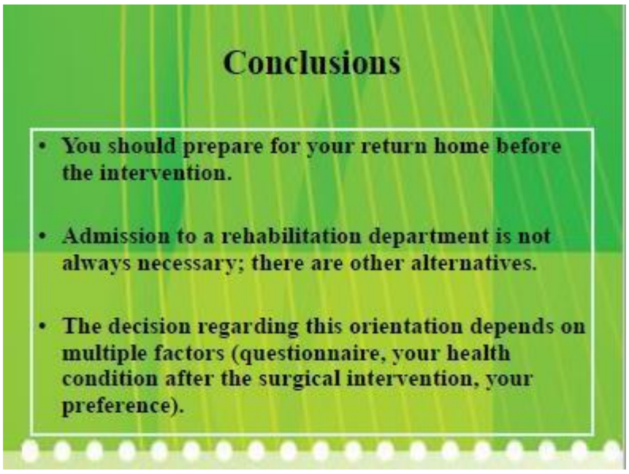

© 2022 Nguyen C et al. *JAMA Network Open.*

eAppendix 5. English translation of the slideshow used for the educational program on diet and weight management conducted by a dietician.

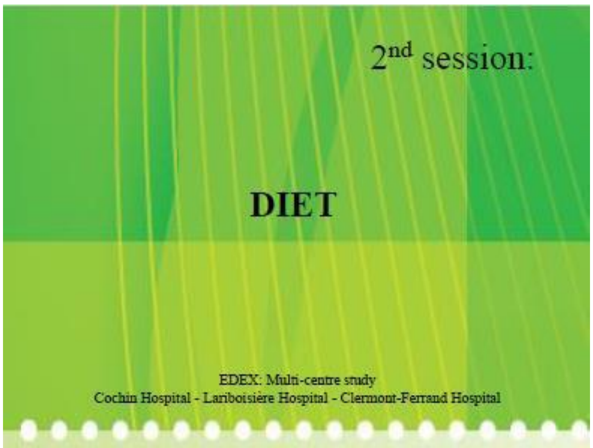

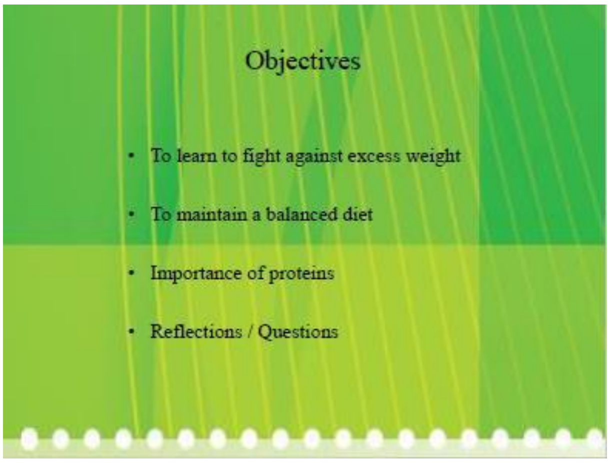

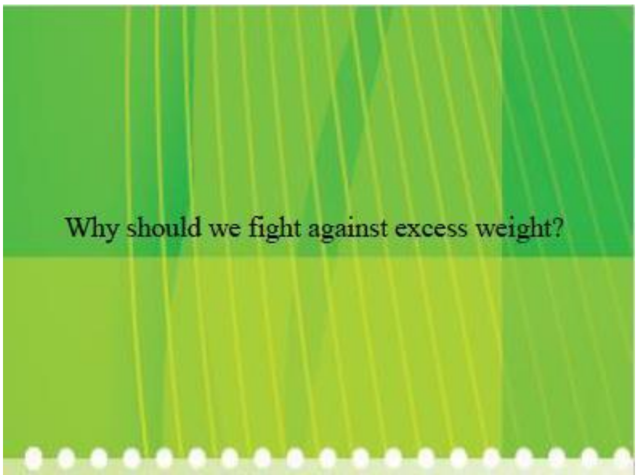

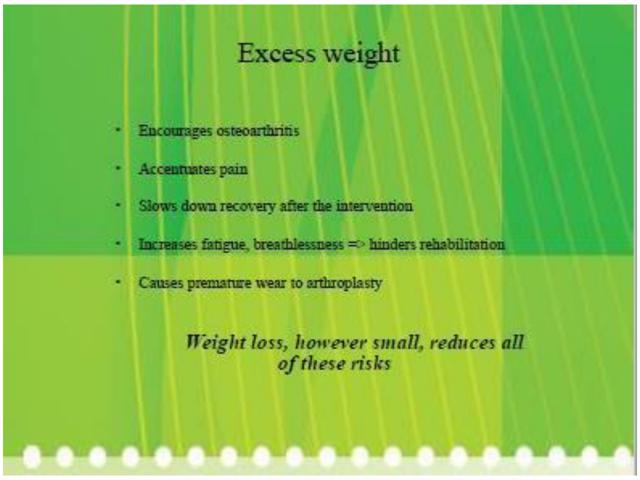

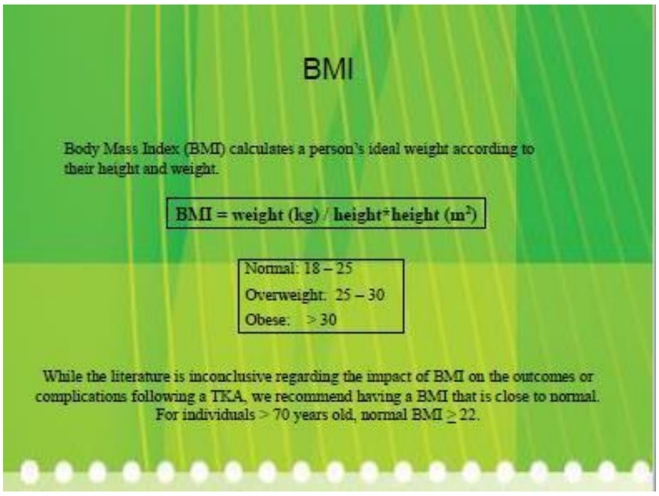

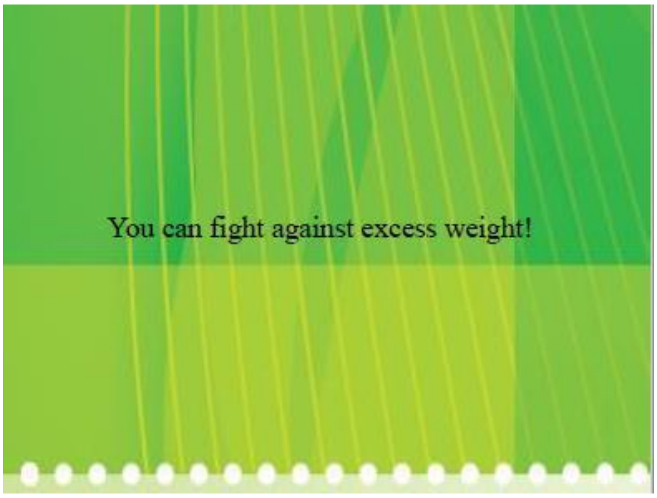

© 2022 Nguyen C et al. *JAMA Network Open.*

Following a diet does not mean stopping eating, but rather eating a balanced diet.

Balanced diet

- Eat 3 meals/day (± a snack)
- Eat plenty of fruit and vegetables
- Eat proteins
- Avoid snacking and eating excessively
- Avoid sugary drinks (alcohol, soda, etc.)
- Drink 1 to 1.5 litres of water/day

**DAIRY PRODUCTS:** Milk, dairy products, cheese, dessert creams	**Proteins ± lipids<br>Calcium<br>Vitamins A, D, B_{12}**
**MEAT, FISH, EGGS**	**Proteins, lipids<br>Iron, selenium, zinc<br>Vitamin B**
**FRUIT AND VEGETABLES**	**Carbohydrates<br>Vitamin C, K, B9 (folic acid)<br>Fibres**
**STARCHES / BREAD AND DERIVED PRODUCTS**	**Complex carbohydrates<br>Vegetable proteins<br>Vitamin B<br>Fibres**
**FATS:** Butter, oil, fresh cream, etc.	**Lipids<br>Vitamin A and E<br>Essential fatty acids**
**SUGAR PRODUCTS:** Sugar, confectionery, fizzy drinks, etc.	**Simple carbohydrates (optional, particularly in the event of excess weight)**

Eat a little from each food group every day

Why are proteins important?

- To preserve and regenerate muscle mass
- To encourage healing
- Good muscle mass facilitates rehabilitation and resuming walking.
- To fight inflammation and complications

Protein intake

- 1.2 g / kg / day
- E.g., for an individual weighing 60 kg: 70 g of protein/day
- E.g.:
  200 g of meat, fish, ham, eggs
  + 3 to 4 dairy products (yoghurt, white cheese, cheese, milk).

Conclusions

- Excess weight leads the knee arthroplasty to wear prematurely.
- You can fight against excess weight.
- You can learn to follow a balanced diet.
- Do not forget that proteins are important in your diet.

© 2022 Nguyen C et al. *JAMA Network Open.*

eAppendix 6. English translation of the slideshow used for the educational program on the management of stress and anxiety in the perioperative period conducted by a psychologist.

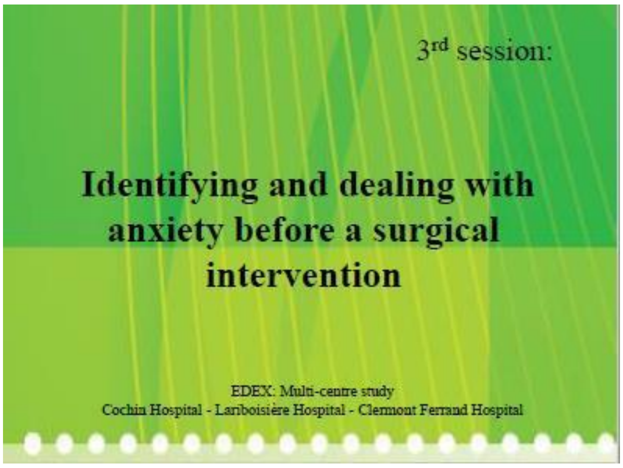

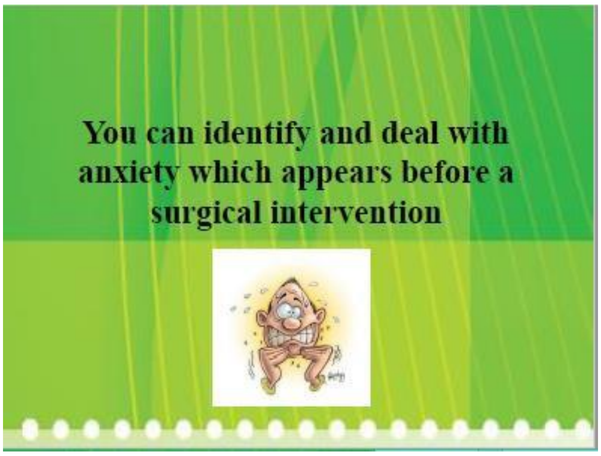

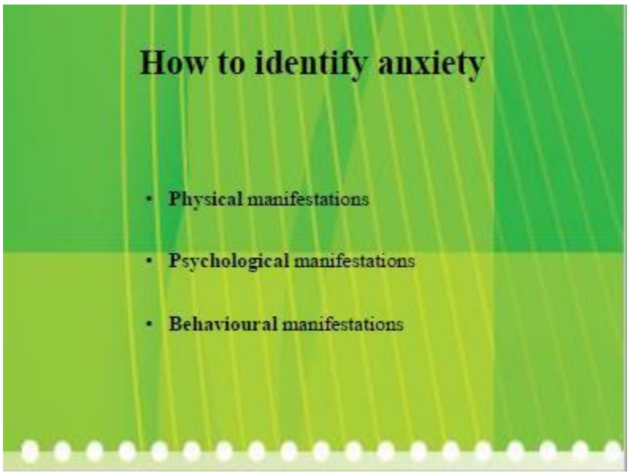

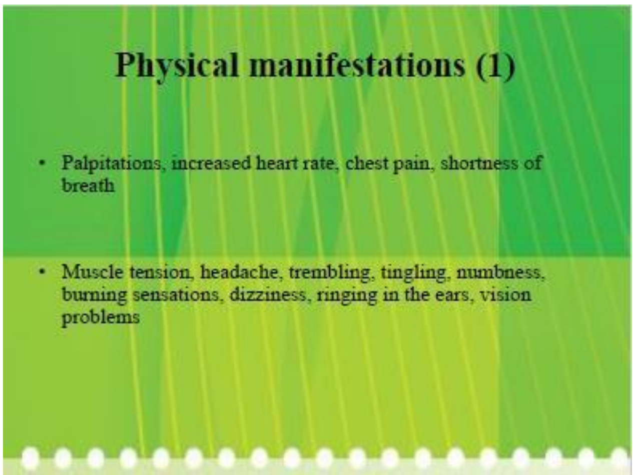

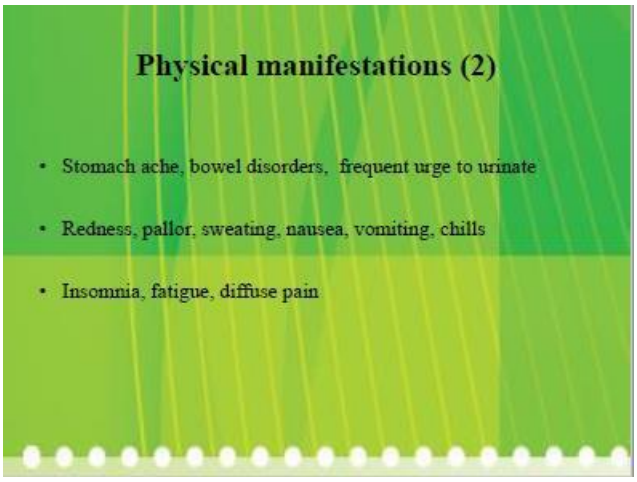

© 2022 Nguyen C et al. *JAMA Network Open.*

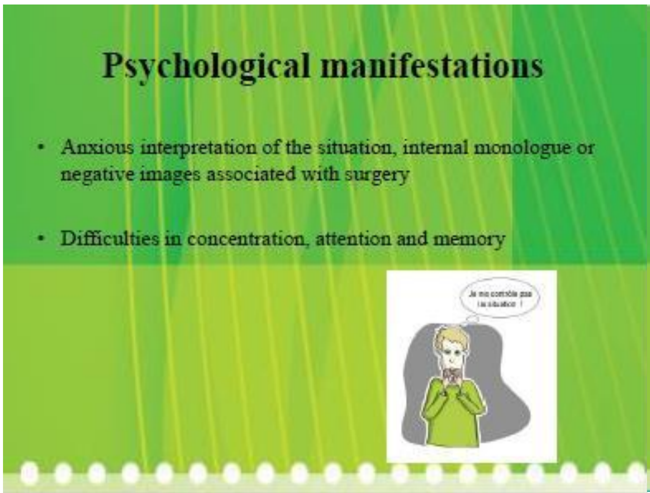

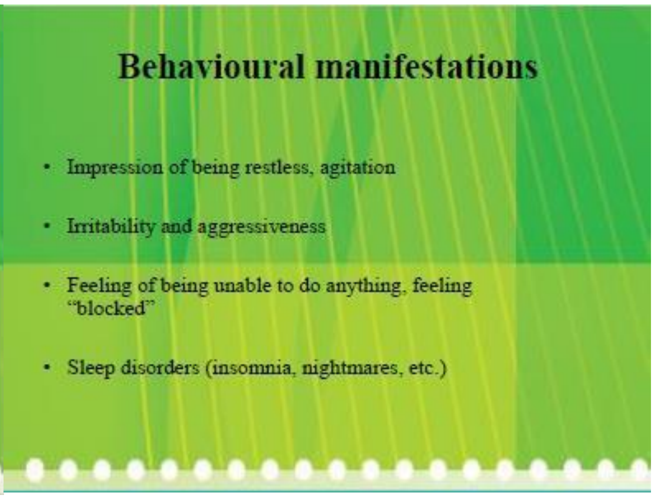

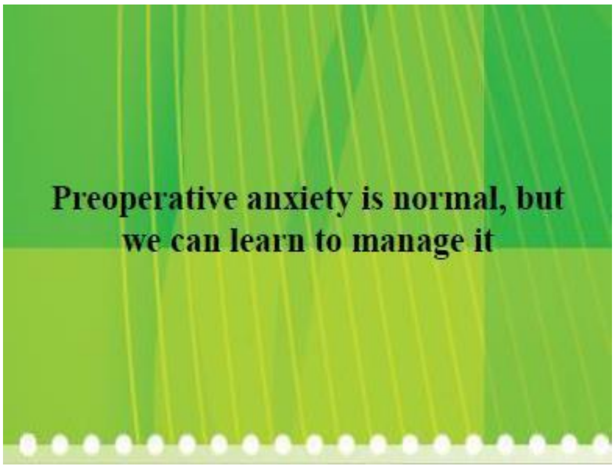

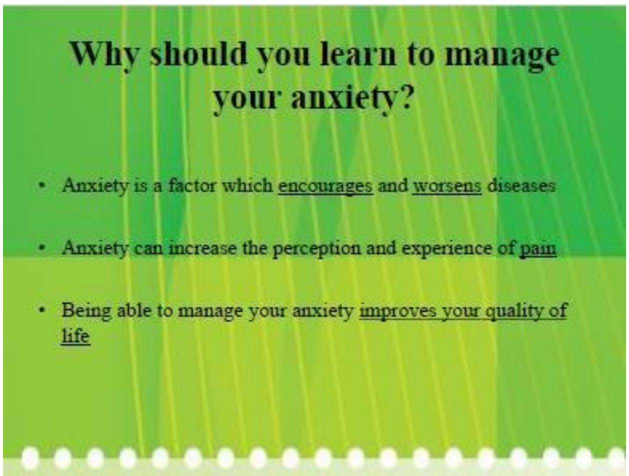

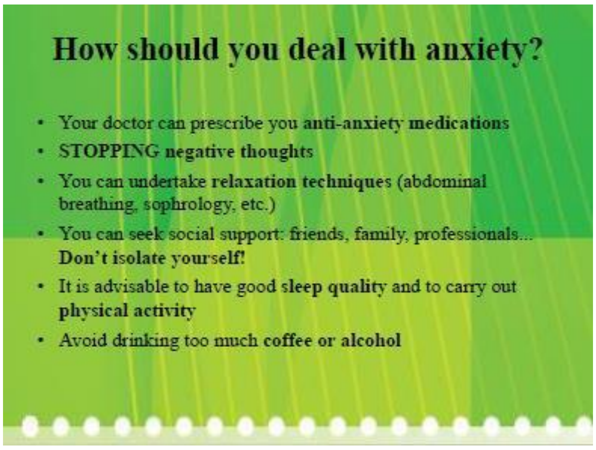

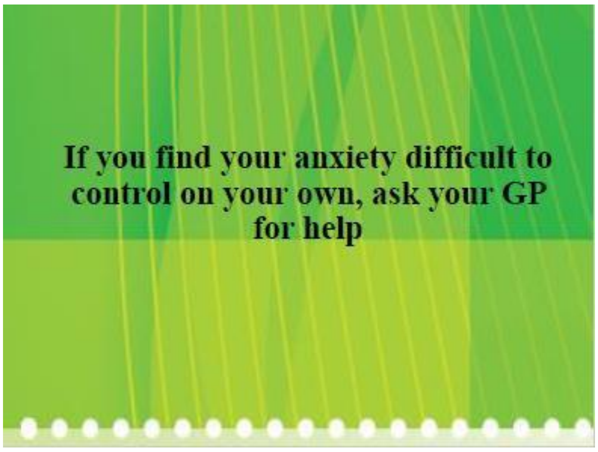

© 2022 Nguyen C et al. *JAMA Network Open.*

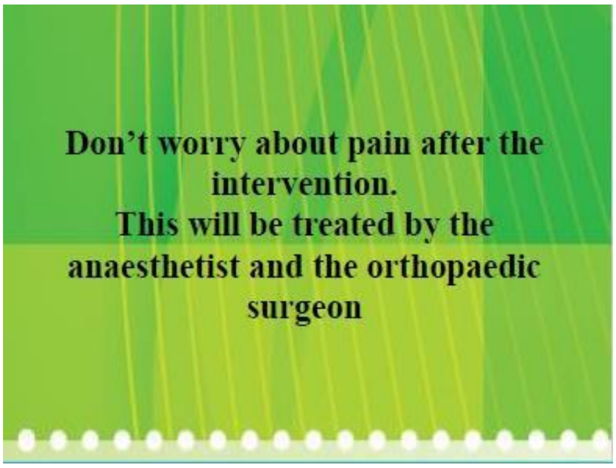

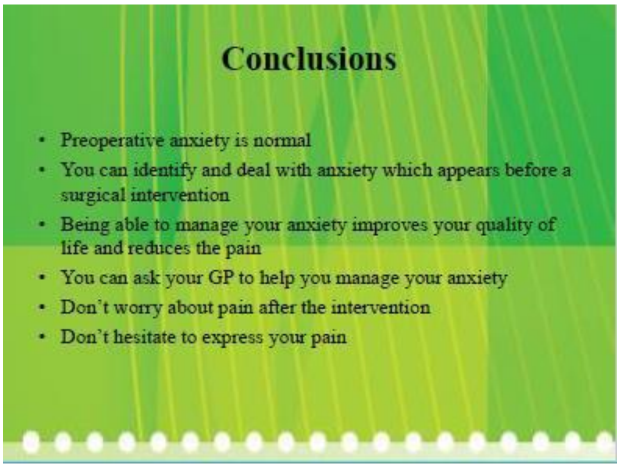

eAppendix 7. English translation of the slideshow used for the educational program on return to home conducted by an occupational therapist.

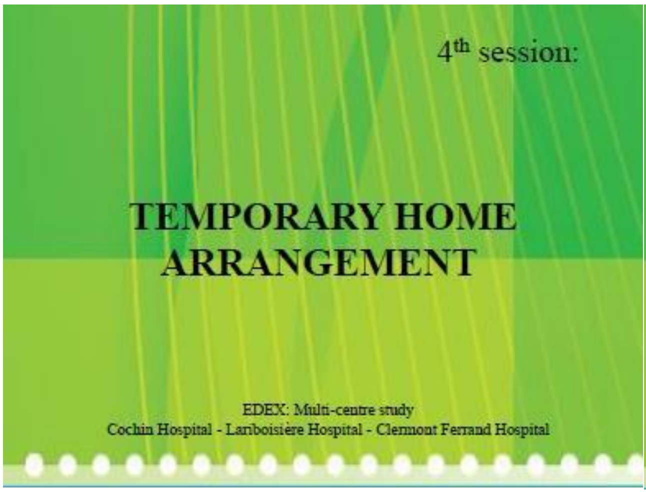

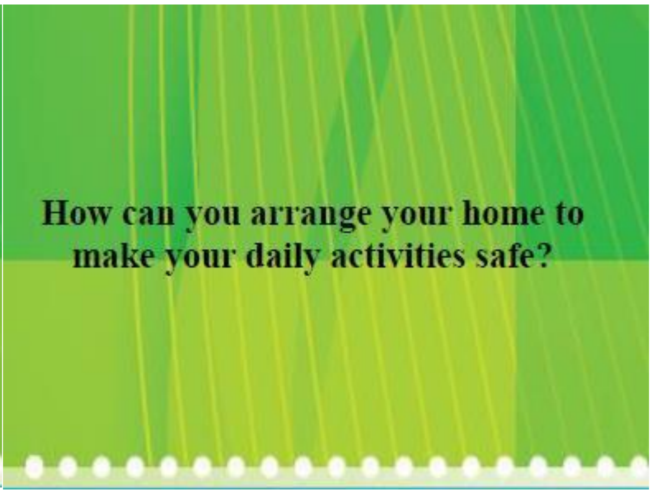

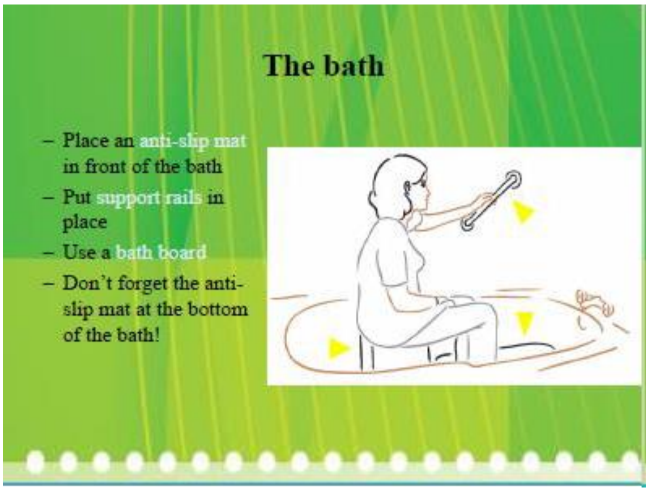

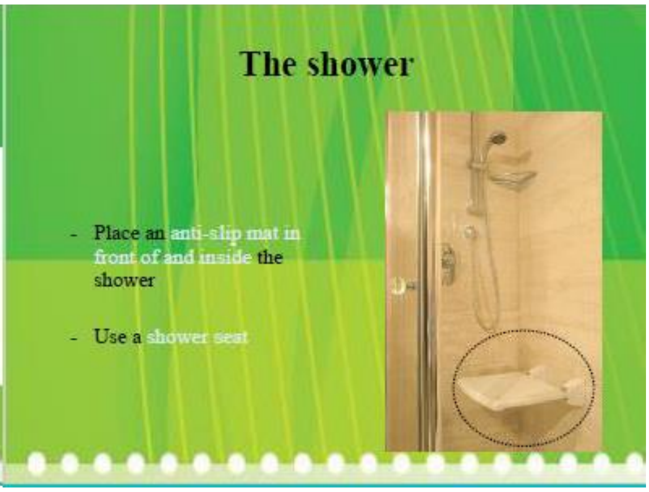

© 2022 Nguyen C et al. *JAMA Network Open.*

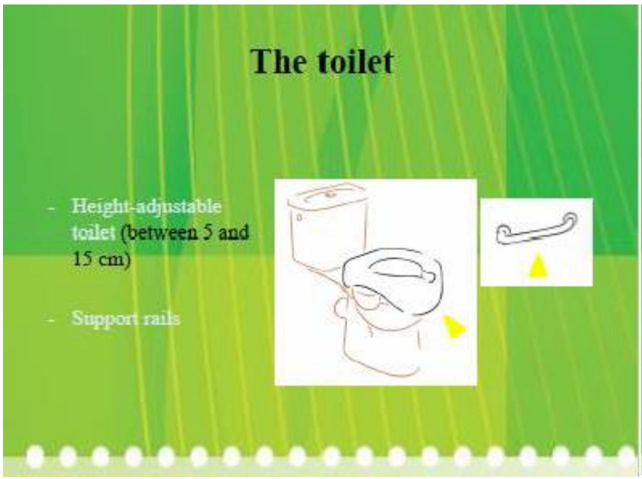

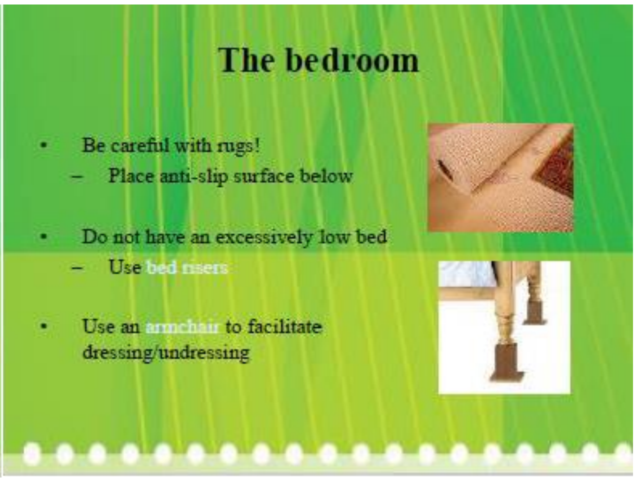

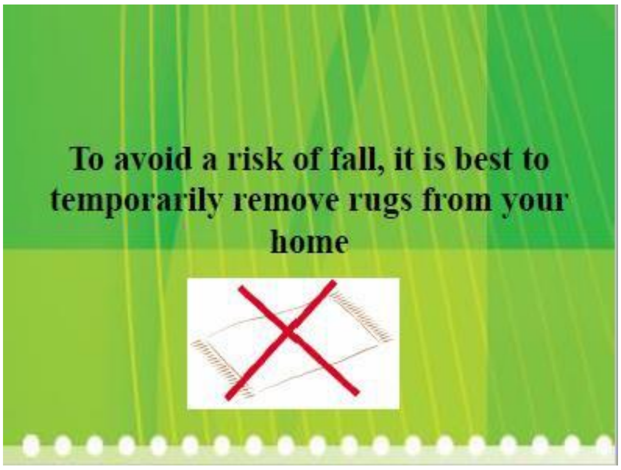

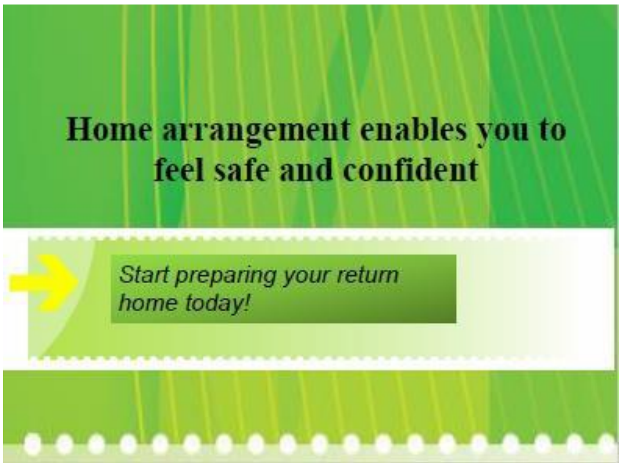

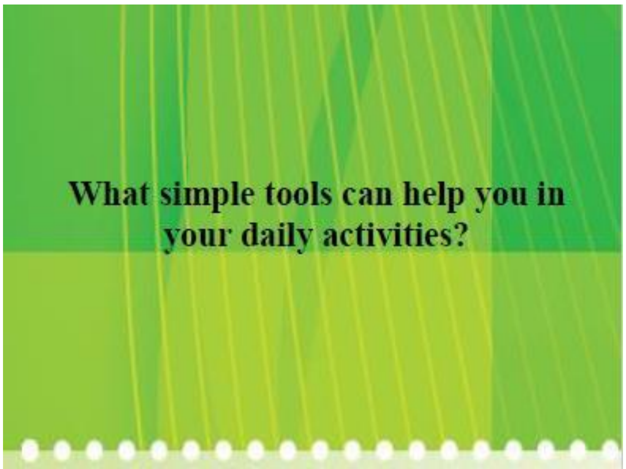

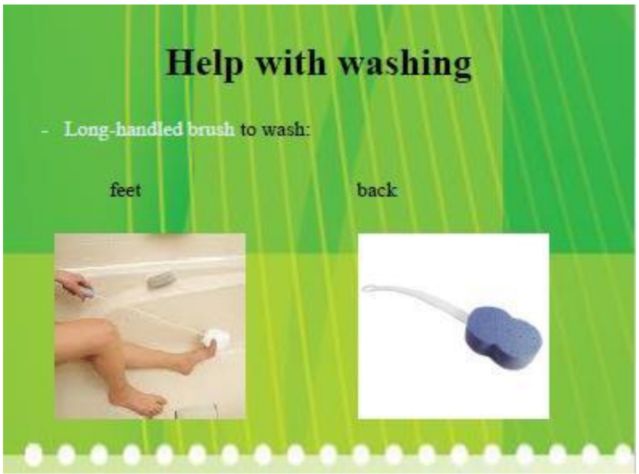

© 2022 Nguyen C et al. *JAMA Network Open.*

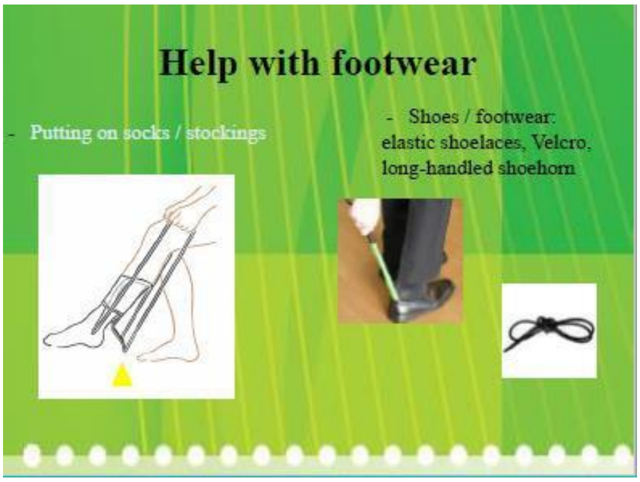

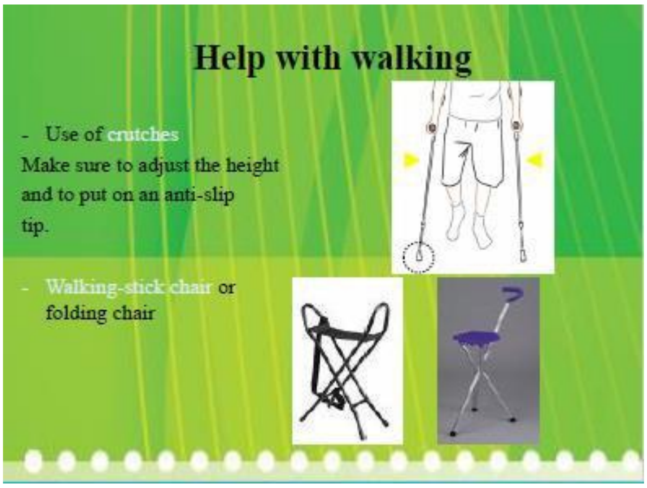

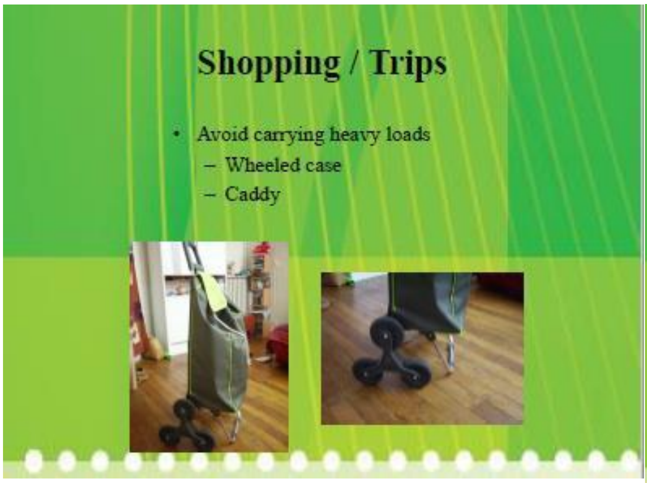

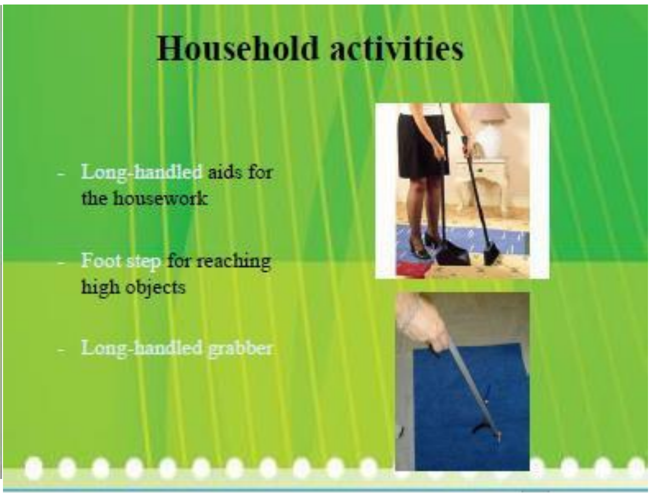

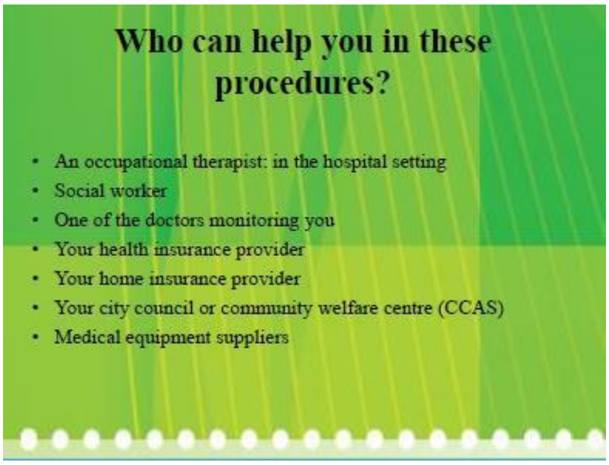

© 2022 Nguyen C et al. *JAMA Network Open.*

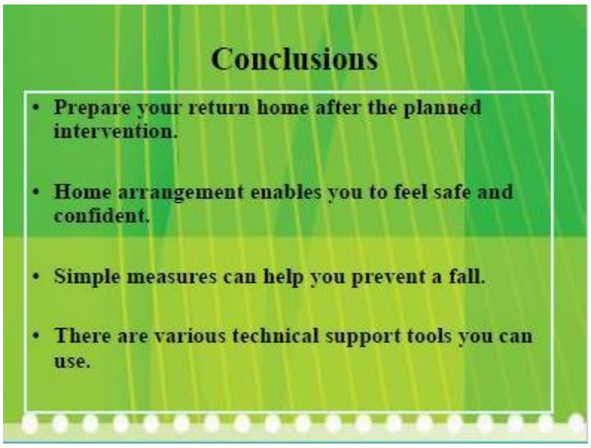

© 2022 Nguyen C et al. *JAMA Network Open.*

eAppendix 8. Supervised exercise therapy sessions.

During the **1st session**, patients were taught a home exercise program to do until knee surgery.

I. <u>Muscle strengthening</u>

- ***Quadriceps***
  - Sitting on a chair, back straight, the heel resting on a step.
  - Raise your foot and extend your knee, ankle lifted.
  - Hold the position for 6 seconds. Repeat 10 times.

- ***Lateral knee stabilisers***

  Sitting on a chair, back straight. Place a stool in front of you.

  - Place your feet on both sides of the legs of the stool. Use the medial border of the feet to tighten the legs, don’t move your knees.
  - Do the same exercise but with your feet inside of the stool. Use the lateral border of the feet to spread the legs of the stool, don’t move your knees.
  - Hold the position for 6 seconds. Repeat 10 times.

© 2022 Nguyen C et al. *JAMA Network Open.*

- ***Hamstrings***
  - Sitting on a chair, back straight, knees slightly flexed, legs crossed at the ankles level
  - Push the front leg back against the rear leg which resists to the movement.
  - Hold the position for 6 seconds. Repeat 10 times.

II. **Muscle stretching**:

- ***Rectus Femoris***
  - Standing on a foot, looking ahead, holding a piece of furniture for balance. Contract abdominal and buttock muscles to immobilize your pelvis. Grab your ankle with your hand (or using a scarf if you are unable to hold your ankle with the hand). Pull knee back without moving your pelvis and your lower back. Hold the position for 20 seconds, repeat 2 times, on each side (if needed).

- ***Hamstrings***
  - Lying on your back, knees bent, feet on the floor. Raise your leg (holding the thigh with both hands), extend the knee by pushing the heel towards the ceiling. Hold the position for 20 seconds, repeat 2 times, on each side (if needed).

- ***Hamstrings and Triceps Surae***
  - Standing, holding a piece of furniture for balance. Place one foot (lifted so that it leans on the heel) on a step, feet and pelvis are facing the same direction.
  - Lean forward to flex the trunk from the hip, keep your back straight, your knee extended and your ankle lifted. Hold 20 seconds, repeat 2 times, on each side (if needed).

© 2022 Nguyen C et al. *JAMA Network Open.*

III. Knee posture, balance and proprioception:

- Knee posture to prevent or correct a lack of knee extension
  - Sitting on a chair, back straight, one foot, leaning by the heel on a stool, knee in the air.
  - Hold the posture for 20 minutes trying to extend the knee as far as possible.
  - If you feel your knee hot and/or painful, you can put a cold pack (wrapped in a cloth) on the knee during the posture.

- Improving balance
  - Standing, in front of a piece of furniture (for sake of safety), one foot on a cushion, knee slightly bent.
  - Raise the other foot and try to hold your balance for a few seconds (without holding the piece of furniture).
  - NB: if you consider this exercise too difficult, do it without the cushion (i.e. foot on the floor)

- Knee mobility

  Material: a skateboard or a ball

  - Sitting on a stool, back straight, one foot on a ball.
  - Extend and flex your knee as far as possible by rolling the ball forward and backward under your foot (in knee extension, only your heel will be in contact with the ball).
  - Hold the position (maximal knee flexion/ extension) for 20 seconds, repeat 2 times, on each side (if needed).

© 2022 Nguyen C et al. *JAMA Network Open.*

2nd to 4th sessions: those sessions were dedicated to learning transfers technics, to use crutches to walk and to go up and down stairs. It was a preparation to the post-operative period.

I. Learning how to transfer

The physiotherapist shows each activity and then the patient practices it.

A. Supine to sitting transfer

The patient is in supine position in a medical bed.

The patient uses his contralateral leg to support his operated one.

- Using the over bed pole hoist,
- The patient moves as close as possible to the edge of the bed.
- Then with the help of both arms, rotates to sit down.

© 2022 Nguyen C et al. *JAMA Network Open.*

B. Sitting to standing transfer

1. Sitting on the bed, the patient slides the operated leg forward and bent the contralateral knee in order to be able to push one the contralateral foot to stand up.

2. The patient push on his contralateral leg and on his arms to get up.

C. Standing to sitting in an armchair transfer

1. The patient moves backward in order to feel the seat behind his contralateral leg. He holds the armrests and slips the operated leg forward

2. Using both arms and the contralateral leg, he slows down the sitting.

© 2022 Nguyen C et al. *JAMA Network Open.*

It is recommended to leave the operated leg extended on a stool (in order to prevent or treat a lack of knee extension)

II. Crutches:

A. Learning how to walk with crutches

Starting position: the two feet are parallel and the two crutches on each side of the body.

1. The patient moves the two crutches forward.

2. He moves the operated leg between the 2 crutches

3. Then he moves the contralateral foot forward, in front of the operated leg and of the crutches.

B. Learning how to go up and down the stairs with 2crutches

The crutches are used to reduce the pressure on the operated leg on analgesic purpose

- Going up:

The patient moves his contralateral leg first: foot on the first step, then leaning on this contralateral leg, he moves both crutches on the step, and finally pushing on his crutches and on the contralateral leg, he moves the operated leg on the same step.

- Going down:

© 2022 Nguyen C et al. *JAMA Network Open.*

First leaning on his contralateral leg, the patient moves the two crutches down on the first step, then he moves the operated leg and finally, pushing on his crutches, he moves the contralateral leg on the same step.

The patient practices until he is able to move without or with few instructions.

III. Revision the home exercises program

The last session was dedicated to the revision of the home exercises program. In addition regular aerobic activity (walking, cycling on training bike…) was recommended to patient.

The minimum recommended program consisted in 3 exercise sessions of 20 to 30 minutes and 1 period of 40 minutes of aerobic activity per week.

© 2022 Nguyen C et al. *JAMA Network Open.*

eAppendix 9. Non-pharmacological and pharmacological co-interventions.

	**Rehabilitation educationN = 131**	**Usual care N = 131**	**All N = 262**
**Between inclusion and 3-month follow-up**			
Analgesics	58/80 (73)	75/97 (77)	133/177 (75)
Non-steroidal anti-inflammatory drugs	21/81 (26)	20/99 (20)	41/180 (23)
Knee brace	6/79 (8)	7/99 (7)	13/180 (7)
Foot orthoses	6/81 (7)	9/99 (9)	15/180 (8)
Physical therapy	73/81 (90)	78/99 (79)	151/180 (84)
Exercise therapy	36/81 (44)	48/99 (49)	84/180 (47)
Walking aids	6/79 (8)	7/99 (7)	13/178 (7)
Weight management	6/81 (7)	9/99 (9)	15/180 (8)
Alternative medicines	73/81 (90)	78/99 (79)	151/180 (84)
**Between 3- and 6-month follow-up**			
Analgesics	35/60 (58)	46/65 (71)	81/125 (65)
Non-steroidal anti-inflammatory drugs	17 /59 (29)	22/66 (33)	39/125 (31)
Knee brace	5/57 (9)	9/65 (14)	14/122 (12)
Foot orthoses	10/59 (17)	12/66 (18)	22/125 (18)
Physical therapy	40/60 (67)	41/66 (62)	81/126 (64)
Exercise therapy	25/59 (42)	23/66 (35)	48/125 (39)
Walking aids	5/57 (9)	9/65 (14)	14 /122(12)
Weight management	10/59 (17)	12/66 (18)	22/125 (18)
Alternative medicines	40/60 (67)	41/66 (62)	81/126 (65)
**Between 6- and 12-month follow-up**			
Analgesics	37/59 (63)	34/60 (57)	71/119 (60)
Non-steroidal anti-inflammatory drugs	14/58 (24)	14/60 (23)	28/118 (24)
Knee brace	10/57 (18)	8/60 (13)	18/117 (15)
Foot orthoses	10/57 (18)	13/61 (21)	23/118 (20)
Physical therapy	28/58 (48)	22/61 (36)	50/119 (42)
Exercise therapy	23/58 (40)	16/61 (26)	39/119 (33)

© 2022 Nguyen C et al. *JAMA Network Open.*

Walking aids	10/57 (18)	8/60 (13)	18/117 (15)
Weight management	10/57 (18)	13/61 (21)	23/118 (20)
Alternative medicines	28/58 (48)	22/61 (36)	50/119 (42)
All results are n/N (%).			

© 2022 Nguyen C et al. *JAMA Network Open.*

eAppendix 10. Twelve-month costs in the intervention and control groups; costs are ineuros.

	Rehabilitation educatio nN =131	Usual care N = 131	Adjusted difference in means* (IC 95%)	P-value
**Cost of the program**	62	NA	62	NA
**Initial admission**	9168 (1568)	9065 (1230)	-103 (-559 ; 269)	0.57
**Rehabilitation**	6143 (7005)	6693 (5875)	550 (-1385 ; 2304)	0.51
**Adverse events**	200 (919)	229 (1223)	29 (-276 ; 385)	0.84
**Total**	15573 (7747)	15987 (6519)	414 (-1739 ; 2158)	0.64

© 2022 Nguyen C et al. *JAMA Network Open.*
