# Supplementary material for: Effect of Prehabilitation Before Total Knee Replacement for Knee Osteoarthritis on Functional Outcomes: A Randomized Clinical Trial
Source: JAMA Netw Open. 2022 Mar 9;5(3):e221462. doi: 10.1001/jamanetworkopen.2022.1462 (PMC8908069; doi:10.1001/jamanetworkopen.2022.1462)
Supplement: Supplement 3. — Data Sharing Statement [file jamanetwopen-e221462-s003.pdf]

Data Sharing Statement

Nguyen. Effect of Prehabilitation Before Total Knee Replacement for Knee Osteoarthritis on Functional Outcomes. *JAMA Netw Open*. Published March 09, 2022.
doi:10.1001/jamanetworkopen.2022.1462

Data
Data available: Yes
Data types: Deidentified participant data, Data dictionary
How to access data: The full original protocol and dataset can be accessed by academic researchers by contacting Professor Christelle Nguyen (christelle.nguyen2@aphp.fr) and statistical codes by contacting Mrs. Élodie Perrodeau (elodie.perrodeau@aphp.fr)
When available: With publication

Supporting Documents
Document types: Statistical/analytic code
How to access documents: Statistical codes can be accessed by academic researchers by contacting Mrs. Élodie Perrodeau (elodie.perrodeau@aphp.fr)
When available: With publication

Additional Information
Who can access the data: Researchers whose proposed use of the data has been approved
Types of analyses: For research purpose
Mechanisms of data availability: After approval of a proposal and with a signed data access agreement
